# Backbone‐Bridging Promotes Diversity in Heteroleptic Cages

**DOI:** 10.1002/anie.202012425

**Published:** 2020-12-16

**Authors:** Kai Wu, Bo Zhang, Christoph Drechsler, Julian J. Holstein, Guido H. Clever

**Affiliations:** ^1^ Faculty of Chemistry and Chemical Biology TU Dortmund University Otto-Hahn Straße 6 44227 Dortmund Germany

**Keywords:** coordination cages, host–guest chemistry, self-assembly, solvent effects, supramolecular chemistry

## Abstract

The combination of shape‐complementary bis‐monodentate ligands **L^A^** and **L^B^** with Pd^II^ cations yields heteroleptic cages cis‐[Pd_2_ **L^A^**_2_ **L^B^**_2_] by self‐sorting. Herein, we report how such assemblies can be diversified by introduction of covalent backbone bridges between two **L^A^** units. Together with solvent and guest effects, the flexibility of these linkers can modulate nuclearity, topology, and number of cavities in a family of four structurally diverse assemblies. Ligand **L^A1^**, with flexible linker, reacts in CH_3_CN with its **L^B^** counterpart to a tetranuclear dimer **D1**. In DMSO, however, a trinuclear pseudo‐tetrahedron **T1** is formed. The product of **L^A2^**, with rigid linker, looks similar to **D1**, but with a rotated ligand arrangement. In presence of an anionic guest, this dimer **D2** transforms and a hexanuclear prismatic barrel **P2** crystallizes. We demonstrate how controlling a ligand's coordination mode can trigger structural differentiation and increase complexity in metallo‐supramolecular assembly.

## Graphical abstract

Rich structural variety in shape‐complementary metallo‐supramolecular assemblies is achieved by bridging ligands via their backbones. A series of unprecedented heteroleptic assemblies ranging from dimeric cages over a prismatic structure to a pseudo‐tetrahedron were obtained. The influence of linker flexibility, solvent and presence of guest molecules was studied.

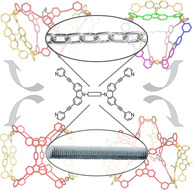

Multivalent interactions, involving the simultaneous binding of several contact sites of one larger molecule to another, for example, a signaling protein and its receptor, occur widely in nature and play significant roles in biological systems.^[1]^ Synthetic supramolecular chemistry studies and utilizes multivalent interactions to stabilize large self‐assembled structures. Many examples of such systems have been reported in the last couple of decades, and most of these architectures feature high symmetries, often based on Platonic or Archimedean bodies. While nature has produced structures of similar shape, assembled in modular fashion (e.g. virus capsids and iron storage proteins), most biological multivalent assemblies are of rather low symmetry and usually hetero‐multimeric. Mimicking such asymmetrical biological systems by artificial supramolecules poses challenges to the employed assembly strategies.^[2]^ The rational design and construction of metallo‐supramolecular assemblies with shapes of low symmetry have recently been elaborated in several research groups by combining sets of different but matching ligands, meant to connect in determined ways via suitable metal nodes. The difficulty to enable formation of a single, integratively self‐sorted species rather than statistic mixtures or narcissistic separation requires sophisticated assembly strategies.^[3]^

With respect to coordination cages, these approaches offer the potential to construct nanoconfinements with well‐defined shape, size, and functional group decoration, able to recognize and convert substrates in their interior in selective ways. The exclusive formation of coordination cages with an asymmetric cavity has been pioneered in a variety of studies. For example, Nitschke reported the employment of a ligand interaction strategy to assemble heteroleptic architectures by exploring the role of interligand π–stacking.^[4]^ Yoshizawa^[5]^ and Fujita^[6]^ demonstrated the exclusive formation of heteroleptic host–guest structures templated by fullerene derivatives and large planar aromatic systems utilizing specific host–guest π–π interactions.

Other work showed that the implementation of subtle donor‐site modifications can lead to the exclusive formation of heteroleptic assemblies with accessible cavities. We suggest to name these approaches “coordination‐sphere engineering”.^[7]^ For instance, Crowley took advantage of secondary interactions, that is, hydrogen bonding between amino groups of adjacent ligand moieties, to steer the assembly of heteroleptic cages.^[8]^ A charge separation approach was employed by Stang to obtain heteroleptic Pt^II^‐based systems.^[9]^ While Fujita pioneered the installation of sterically demanding methyl substituents on aryl ligands L of the widely used *cis*‐[Pd(ethylenediamine)L_2_] motif, we recently increased the scope of this strategy by grouping four picoline‐type ligands with alternating methyl group orientation around “naked” Pd^II^ cations to assemble *cis*‐[Pd_2_L_2_L′_2_] heteroleptic structures.^[10]^

As particularly feasible and versatile strategy, the so‐called “shape‐complementarity” strategy has evolved into a successful way for assembling heteroleptic cages, as demonstrated by Fujita,^[11]^ Mukherjee,^[3c]^ Zhou,^[12]^ Stang,^[13]^ Chand,^[14]^ and us.^[15]^ This strategy utilizes geometric complementarity in two‐component systems to generate heteroleptic supramolecular assemblies in an integrative self‐sorting way.

Most members of this new class of coordination cages, built from the heteroleptic assembly of at least two ligand types, possess a single cavity, often showing a rather low symmetry. Since nature shows us that many biological hosts, such as enzymes, contain multiple binding pockets (e.g. a substrate binding site and a receptor for an allosteric modulator), we became interested in the construction of self‐assembled multi‐cavity systems. So far, two major strategies can be distinguished.^[16]^ The first one makes use of multitopic ligands, for example, tris‐ or even tetrakis‐monodentate bridges, assembling with suitable metal nodes, for example, square‐planar Pd^II^ cations, to yield constructs comprising two or more linearly or circularly aligned cavities of the same shape and size. Several examples of this kind of multi‐cavity cages have been reported by Chand,^[17]^ Crowley,^[18]^ Yoshizawa,^[19]^ and us.^[20]^ The other strategy involves the catenation of single‐cavity cages to give interpenetrated dimers or even higher assemblies, which contain three or more cavities of usually rather small size.[^20b^, ^21^] Few examples of coordination cages have been reported which contain multiple cavities of different sizes.^[22]^ As such structures allow for differentiating guest species, for example, for the realization of cooperative heterotropic receptors, stimuli‐responsive catalysts and logic gates, there is growing interest in the field to allow their rational construction.^[23]^ Very recently, Chand reported a series of novel conjoined‐cages with multiple, non‐uniform cavities by the peripheral decoration of a trinuclear [Pd_3_L_6_] core with one, two, and three units of a [Pd_2_L_4_] entity.^[24]^

Herein, we report on the assembly of a family of heteroleptic multi‐cavity cages by combination of the shape‐complementarity strategy with covalent bridging of two ligands in a back‐to‐back fashion.^[25]^ First, we show that the combination of banana‐shaped carbazole‐based ligand **L^A^** with fluorene‐derived ligand **L^B^** forms a new heteroleptic, *cis*‐configured [Pd_2_ **L^A^**_2_ **L^B^**_2_] cage **C** (Supporting Information, Figures S5–S11). Furthermore, we designed two ligands **L^A1^** and **L^A2^** by bridging a pair of **L^A^** units via their backbone nitrogen positions with two different linker types. **L^A1^** was obtained by using a flexible hexylene chain and **L^A2^** comprises a rigid phenylene linker. Surprisingly, changing the linker's flexibility resulted in modulating the size, shape, and cavity‐count of the formed heteroleptic assemblies to a much greater extent than expected (Figure 1).

**Figure 1:**
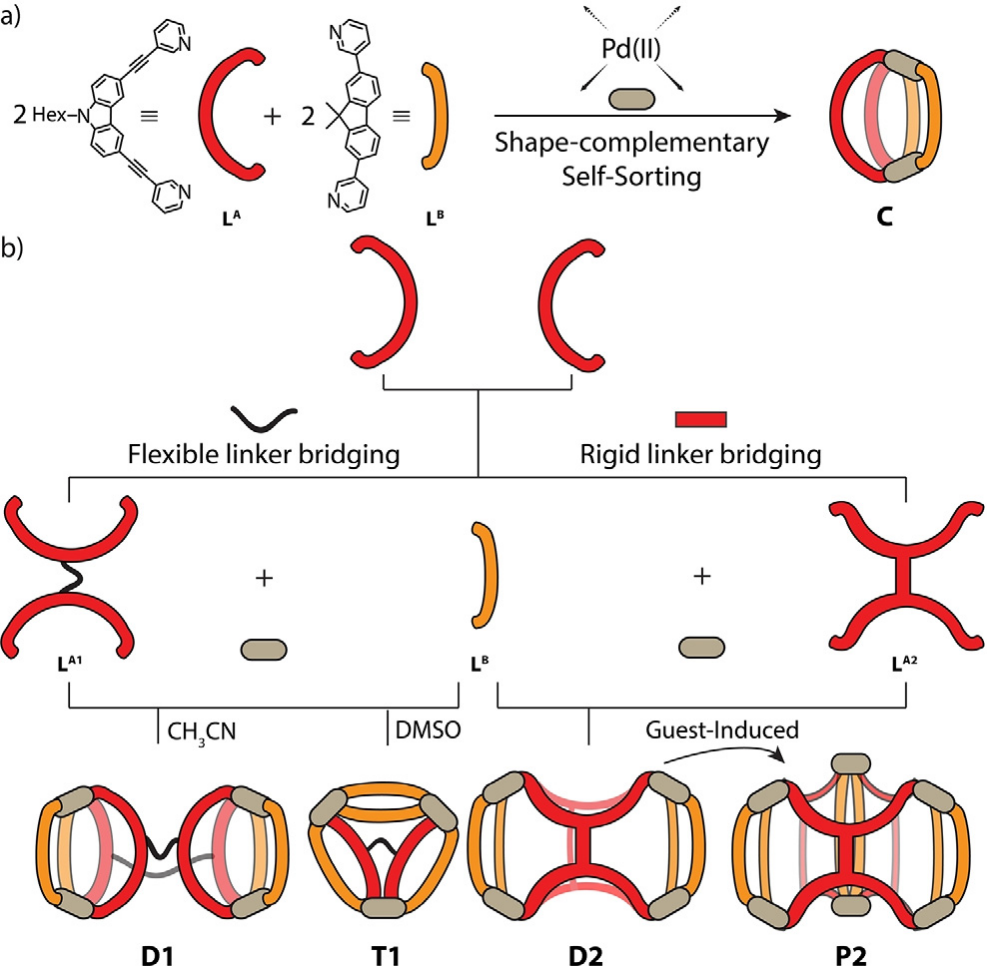
Heteroleptic cages featuring different levels of complexity. a) Shape‐complementary formation of *cis*‐configured [Pd_2_ **L^A^**_2_ **L^B^**_2_] cage **C**; b) introduction of backbone bridges, combined with shape‐complementarity, yields a variety of multi‐cavity assembly products, determined by an interplay of bridge flexibility, solvent, and anionic guests.

Ligands **L^A1^** and **L^A2^** were synthesized by fourfold Sonogashira cross‐coupling reactions starting from corresponding tetra‐iodinated precursors (Supporting Information, Figures S1–S4). First, **L^A1^** was tested for homoleptic cage assembly with [Pd(CH_3_CN)_4_](BF_4_)_2_ in DMSO[D_6_] at room temperature, resulting in the quantitative formation of one species, as indicated by ^1^H NMR and DOSY spectroscopy (Supporting Information, Figures S12–S15). HR‐ESI mass spectrometry revealed the formation of dinuclear cage [Pd_2_(**L^A1^**)_2_] (**C1**), most probably resembling its untethered predecessor [Pd_2_(**L^A^**)_4_]^[26]^ but with pairs of neighboring bis‐monodentate ligands connected via their backbones (Supporting Information, Figures S16, S46). Next, we examined **L^B^** for homoleptic assembly formation in DMSO[D_6_] and obtained a 1:1 mixture of a 3‐membered ring [Pd_3_(**L^B^**)_6_] (**R**) and a [Pd_4_(**L^B^**)_8_] tetrahedron (**T**),^[27]^ revealed by ^1^H NMR and HR‐ESI MS (Supporting Information, S17–S21). Then we combined tethered **L^A1^** with shape‐complementary ligand **L^B^** ^[28]^ to study formation of heteroleptic assemblies. Our first experiments in CD_3_CN resulted in a rather complicated NMR spectrum, indicating the formation of a mixture of several species. HR‐ESI MS clearly showed the existence of at least five components of different ligand composition ([Pd_3_(**L^B^**)_6_], [Pd_4_(**L^B^**)_8_], [Pd_2_(**L^A1^**)(**L^B^**)_2_], [Pd_4_(**L^A1^**)_2_(**L^B^**)_4_], and [Pd_3_(**L^A1^**)(**L^B^**)_4_]), most probably resulting from the high structural flexibility of **L^A1^** (Supporting Information, Figures S22, S23). Remarkably, we were able to crystallize a single species out of this mixture by vapor diffusion of ethyl ether into the CD_3_CN solution. The structure of this compound **D1**, obeying the formula [Pd_4_(**L^A1^**)_2_(**L^B^**)_4_], can be described as a “dimer” of heteroleptic cage monomers **C**, connected back‐to‐back by two rope‐like linkers.^[29]^ It crystallized in monoclinic space group *C*2/*c* and possesses *C*_2_ symmetry with only half of the dimer in the asymmetric unit. The Pd⋅⋅⋅Pd distance of 13.6 Å in each cage monomer is almost the same as in the parent heteroleptic cage **C** (Figure 4 a and Supporting Information, Figure S45). Besides the two cavities resembling **C**, occupied by acetonitrile molecules and BF_4_^−^ anions, the close connection of the two cage units via two tethers creates a further, wedge‐shaped central space flanked by the carbazole ligand moieties of both cage units. While this void is primarily defined by two coplanar aromatic ligand panels in about 6.8 Å distance, no guests were found to be bound in the solid or solution state (Supporting Information, Figure S51).

As solvent effects play significant roles in determining the outcome of supramolecular assembly,^[30]^ we were interested in finding out whether the combination of **L^A1^** with **L^B^** leads to a pure product when using DMSO[D_6_] as the solvent. ^1^H NMR analysis yielded a very complicated, yet well‐resolved spectrum with a total of 42 signals in the range of *δ*=6.9–10.4 ppm (Figure 2 b). HR‐ESI MS shows a series of peaks, which can be assigned to [Pd_3_(**L^A1^**)(**L^B^**)_4_+*n* BF_4_]^(6−*n*)+^ (*n*=0–4; Figure 3 a). DFT‐based molecular modeling suggests that a heteroleptic structure with the formula [Pd_3_(**L^A1^**)(**L^B^**)_4_] composed of this ligand combination may be one of two possible isomers, having entirely different topologies (Supporting Information, Figure S47). In the one case, both carbazole moieties of **L^A1^** would span the same edge of a 3‐membered ring **R1** (both other edges composed of a pair of ligands **L^B^**), while in the other case, a structure **T1** would result in which one edge would be connected by a pair of ligands **L^B^** and two edges feature a combination of one bridging **L^B^** and one bis‐monodentate carbazole moiety of **L^A1^**. Since the flexible hexylene loop in **L^A1^** occupies space on one face of the triangular base formed from the connected ligands, the whole structure **T1** adopts a pseudo‐tetrahedral shape. Careful analysis of NMR splitting patterns and indicative NOESY cross‐peaks allowed us to rule out the **R1** topology (Figures 2 b, 4 c). We managed to assign all ^1^H NMR peaks from 2D spectra (Supporting Information, Figures S26, S27). All proton signals originating from **L^B^** were split into four sets, while for **L^A1^** two sets of peaks resulted. The exclusive formation of **T1** was further verified by a ^1^H DOSY NMR spectrum, showing that all 42 peaks in the aromatic region correspond to the same diffusion coefficient, with the calculated hydrodynamic radius of 13.9 Å comparable to the dimensions of the modelled structure. Unfortunately, all attempts to obtain single crystals for **T1** failed. Its DFT‐derived model, however, proved suitable to explain the different splitting modes observed for **L^A1^** and **L^B^** in its NMR spectrum. The model reveals that for ligand **L^A1^**, the upper halves (brown color) and the lower halves (red color) have different chemical environments due to their arrangement along the base of the 3‐membered ring, resulting in two sets of NMR signals (Figures 2 b, 4 b). Ligands **L^B^**, combined with the **L^A1^** moieties on these edges, likewise split in two magnetically inequivalent halves (blue and purple color). In addition, the edge spanned by two counts of **L^B^** has distinguishable upper and lower faces (yellow and green). Taken together, four different chemical environments for **L^B^** can be anticipated, exactly as observed in the experimental NMR spectrum.

**Figure 2:**
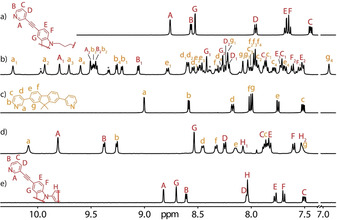
Partial ^1^H NMR (500 MHz/DMSO[D_6_], 298 K) spectra showing the assembly of heteroleptic pseudo‐tetrahedron [Pd_3_(**L^A1^**)(**L^B^**)_4_]^6+^ (**T1**) and heteroleptic cage dimer [Pd_4_(**L^A2^**)_2_(**L^B^**)_4_]^8+^ (**D2**). a) **L^A1^**; b) **T1** (*=[Pd_3_(**L^B^**)_6_]^6+^ ring **R**); c) **L^B^**; d) **D2** obtained by heating a 1:2:2 mixture of **L^A2^**, **L^B^**, and Pd^II^; e) **L^A2^**.

**Figure 3:**
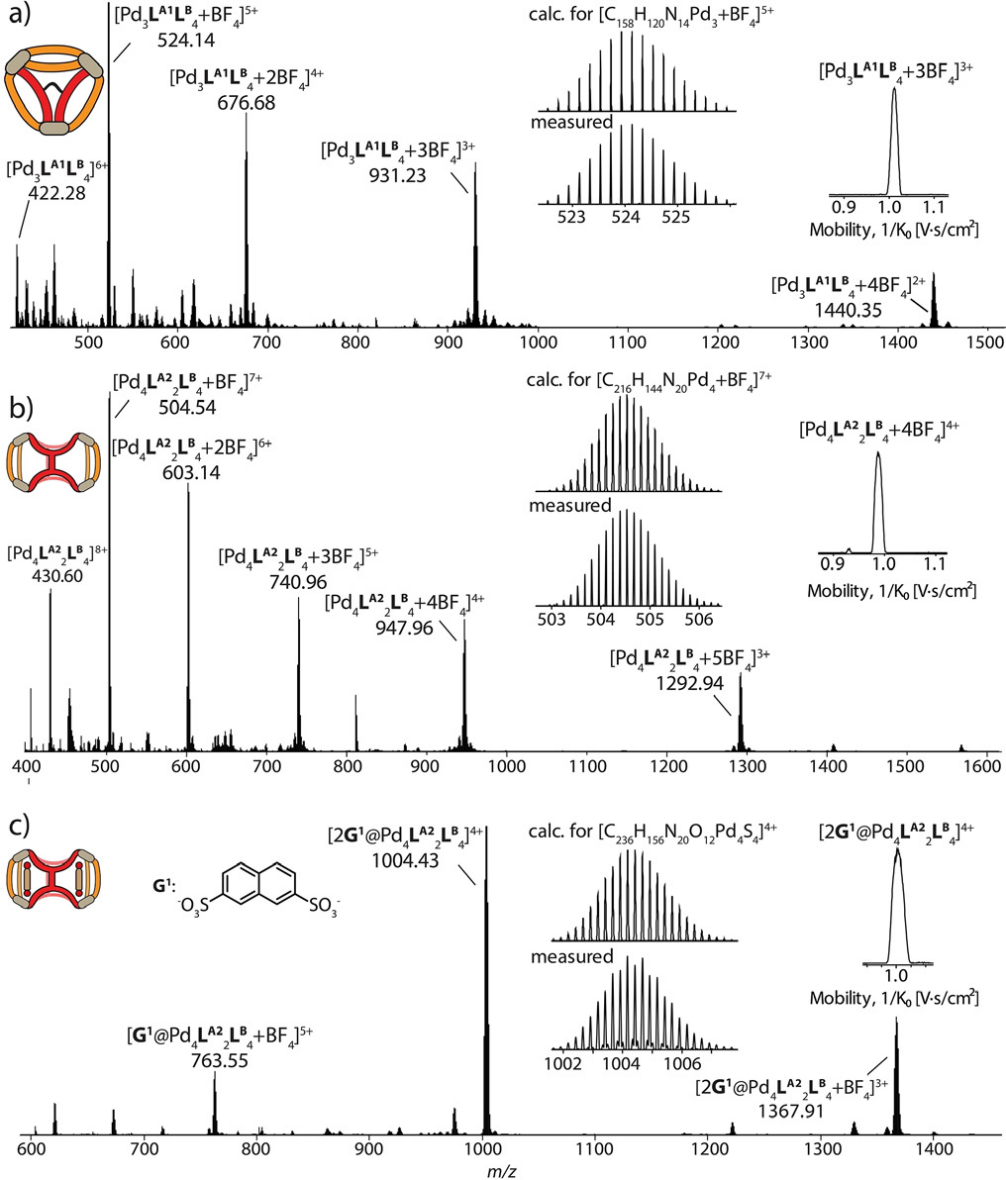
HR‐ESI mass spectra of a) pseudo‐tetrahedron **T1**; b) cage dimer **D2** and c) host–guest complex **2G^1^**@**D2**. Insets show ion mobility traces.

**Figure 4:**
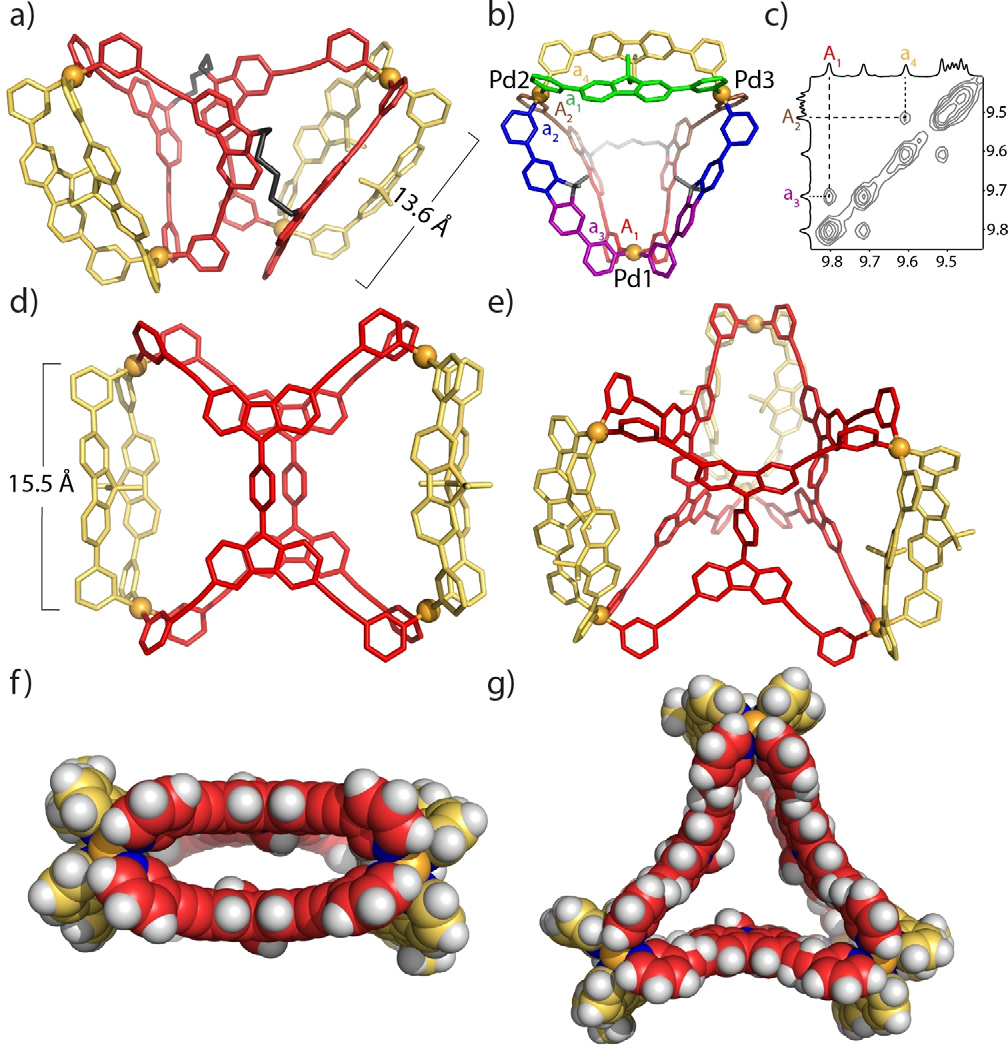
a) X‐ray structure of **D1**;^[34]^ b) DFT‐optimized model of **T1** (colors indicate different ligand environments); c) NOESY NMR details supporting the **T1** structure; d) side‐ and f) top‐view of X‐ray structures of **D2** ^[34]^ and e) and g) of **P2**;^[34]^ (**G^1^**, BF_4_^−^ anions and solvent molecules omitted for clarity).

In addition, we employed trapped ion mobility measurements, coupled with high‐resolution ESI‐TOF mass spectrometry (ESI‐TIMS‐TOF) to gain more insight into the gas‐phase dimensions of the structure.^[31]^ We found that indeed the experimentally determined collisional cross section values (eCCS) fit better to the calculated theoretical ones (tCCS) of the modelled **T1** structure than that of tentative ring **R1** (Supporting Information, Table S2). To the best of our knowledge, such a heteroleptic, pseudo‐tetrahedral structure has never been reported before.

Subsequently, we examined the behavior of ligand **L^A2^**, comprising a rigid phenylene linker, for cage assembly. First, the formation of homoleptic species was examined, resulting in extremely broadened NMR spectra in different solvents, thus indicating the formation of polymeric species. This is not surprising in light of the divergent nature of the rigid, tetra‐monodentate ligand. Subsequently, we reacted ligands **L^A2^** and **L^B^** with Pd^II^ in a 1:2:2 mixture in DMSO[D_6_] or DMF[D_7_] at 80 °C for 8 h, giving only one set of clear signals with all proton signals assignable to the coordinated forms of **L^A2^** and **L^B^** (Figure 2 d). The pyridyl proton signals next to nitrogen donors on both ligands (H_A_, H_B_, and H_a_, H_b_) have moved significantly to the downfield region compared to the free ligands, indicative of Pd^II^ coordination. ^1^H DOSY confirmed that all proton signals belonged to the same diffusion coefficient, with a hydrodynamic radius of 17.2 Å (Supporting Information, Figure S34). In the NOESY spectrum, clear cross‐peaks were observed between protons H_a_ and H_A_, as well as between H_b_ and H_B_, further showing that both ligand **L^A2^** and **L^B^** are integral components of the same assembly **D2** (Supporting Information, Figure S33). NMR integration of proton signals for H_A_, H_B_, H_a_, and H_b_ shows a ratio of 1:1:1:1. Interestingly, proton signals for the phenylene linker (H_H1_ and H_H2_) are no longer equivalent.

HR‐ESI MS revealed a formula [Pd_4_(**L^A2^**)_2_(**L^B^**)_4_+*n* BF_4_]^(8−*n*)+^ (*n*=1, 2) via prominent peaks at *m*/*z* 504.54 and 603.14 (Figure 3 b). Further, we were able to obtain single crystals suitable for X‐ray structure analysis by slow vapor diffusion of isopropyl ether into a solution of the assembly in DMF. Compound **D2** crystallizes in monoclinic space group *C*2/*m* with the asymmetric unit containing only one fourth of the discrete metallo‐supramolecular assembly. While the initial visual inspection of the structure may suggest a high similarity of **D2** to **D1**, upon closer look, there is a striking difference: while ligand **L^A1^** in **D1** binds the **L^B^** counterparts in the same mode as observed in parental cage **C**, ligand **L^A2^** has formally been rotated by 90° in its major plane, now using one pyridine donor arm each of both back‐to‐back joined parts of ligand **L^A2^** (Figure 4 d). The Pd⋅⋅⋅Pd distance for cations binding to the same **L^B^** ligands is 15.5 Å for **D2** as compared to 13.6 Å for **D1**, owing to the different shape‐complementary assembly modes. Both units of **L^A2^** are oriented in a coplanar fashion and their central phenylene groups slightly rotate relative to the carbazole planes to minimize steric hindrance. Of each phenylene group, one edge is pointing into the central void and one points outwards (Figure 4 f), most probably leading to the mentioned NMR signal splitting. For each of the two outer parts of the structure, the geminal dimethyl group on one ligand **L^B^** points outside the cavity, while the dimethyl group of the other points to the fluorene backbone of the first **L^B^** to allow CH⋅⋅⋅π interactions (ca. 2.9–3.3 Å).

Interestingly, four DMF solvent molecules are encapsulated in the outer two cavities and interact with the Pd‐complex vertices via CH⋅⋅⋅O hydrogen bonding between the carbonyl group of DMF and the pyridine C−H groups of **D2** (Supporting Information, Figure S55). This inspired us to study the host–guest properties of **D2** by choosing guests with the ability to act as a hydrogen‐bond acceptor. In line with our previous studies,^[15a]^ 2,7‐naphthalene disulfonate (**G^1^**), resembling the shape of the outer cavities, was assumed to be a potential guest. Indeed, NMR titration revealed that with increasing amounts of guest, inward pointing proton signals H_A_ and H_a_ shift downfield significantly, implying encapsulation of **G^1^** inside both cavities of **D2** (Supporting Information, Figure S41). Signal shift ceased after the addition of 2 equiv guest (Δ*δ*=0.36 and 0.69, respectively), suggesting a saturated equilibrium with a 1:2 host/guest ratio. This was verified by the HR‐ESI MS spectrum, in which the most prominent peak at *m*/*z* 1004.43 could be assigned to **2G^1^**@**D2** (Figure 3 c). Association constants were calculated to be *K*_1_=(2.08±0.08)×10^3^ and *K*_2_=(2.11±0.28)×10^3^ m ^−1^ for the first and the second binding event, respectively (^1^H NMR integration averaged over three different host‐guest ratios).^[32]^ The cooperativity parameter, *α*=4*K*_2_/*K*_1_=4, indicates positive cooperative binding, which is surprising given the fact that the first bis‐anionic guest reduces the overall positive charge of the complex (Supporting Information, Table S1).^[33]^ In an attempt to grow single crystals of the host–guest complex, we unexpectedly obtained a new heteroleptic prismatic cage [Pd_6_(**L^A2^**)_3_(**L^B^**)_6_]^12+^ (**P2**) instead. **P2** crystallizes in triclinic space group $P\bar{1}\;$ with one whole trimer molecule in the asymmetric unit. The assembly mode with respect to **L^A^** is similar to **D2** and the average Pd⋅⋅⋅Pd distance is 15.3 Å (Supporting Information, Figure S57). Unexpectedly, **G^1^** is not captured by the three cage cavities in the solid‐state structure. Instead, adjacent trimers are bridged by the sulfonate groups of **G^1^** via C−H⋅⋅⋅O−S hydrogen bonds and SO_3_^−^‐to‐Pd^2+^ electrostatic interactions.

Finally, we were interested whether these higher‐order heteroleptic systems can be formed by the rearrangement of homoleptic precursors.^[15]^ Therefore, **C1** and the **R**/**T** mixture in DMSO were combined in a 3:4 ratio. At room temperature, we found that ligand exchange is hampered by a significant kinetic barrier. Heating the mixture at 80 °C for 2 h, however, led to the clean formation of **T1** according to ^1^H NMR spectra (Figures 5 and Supporting Information, Figures S38, S39).

**Figure 5:**
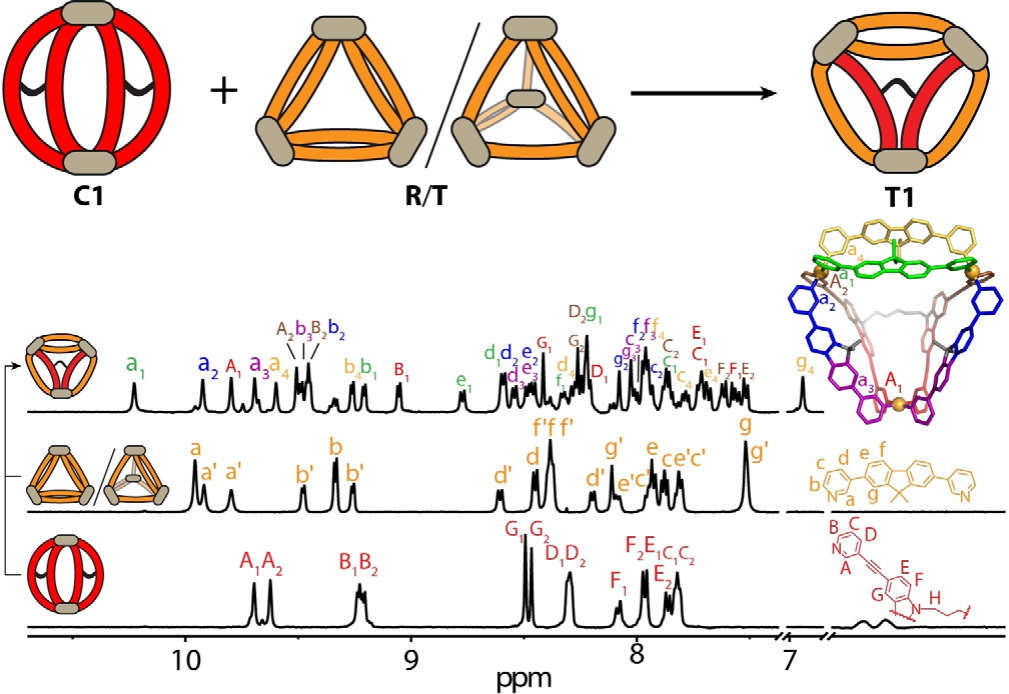
Cage‐to‐cage transformation from homoleptic **C1** and a mixture of **R** and **T** to heteroleptic pseudo‐tetrahedron **T1**.

In summary, we report a novel strategy for the assembly of heteroleptic multi‐cavity cages by fusing ligands via their backbones, allowing to modulate product topology by the choice of linker (rigid or flexible), solvent, and guests. Self‐assembly with flexibly bridged ligand **L^A1^** was found to be a solvent‐dependent process. A heteroleptic pseudo‐tetrahedron **T1**, composed of two different ligands, was obtained in DMSO for the first time, while from CH_3_CN we succeeded to crystallize a heteroleptic cage dimer **D1**. The heteroleptic pseudo‐tetrahedral structure can also be accessed via cage‐to‐cage transformation. Rigid linker‐bridged ligand **L^A2^** readily forms dimer **D2** which can encapsulate up to two bis‐sulfonate guests into its outer two identical cavities in a cooperative fashion, while a cage trimer **P2** was obtained via reorganization during the crystallization process. Our strategy provides the basis for assembling a wider variety of heteroleptic, multi‐cavity cages, allowing to push the complexity of coordination cages, their properties, and application to a higher level.

## Conflict of interest

The authors declare no conflict of interest.

## Supporting information

As a service to our authors and readers, this journal provides supporting information supplied by the authors. Such materials are peer reviewed and may be re‐organized for online delivery, but are not copy‐edited or typeset. Technical support issues arising from supporting information (other than missing files) should be addressed to the authors.

Supplementary
