# Supplementary material for: Backbone‐Bridging Promotes Diversity in Heteroleptic Cages
Source: Angew Chem Int Ed Engl. 2020 Dec 16;60(12):6403–7. doi: 10.1002/anie.202012425 (PMC7986237; doi:10.1002/anie.202012425)
Supplement: Supplementary file 1 — Supplementary [file ANIE-60-6403-s001.pdf]

Angewandte Chemie
Eine Zeitschrift der Gesellschaft Deutscher Chemiker

Supporting Information

Backbone-Bridging Promotes Diversity in Heteroleptic Cages

*Kai Wu, Bo Zhang, Christoph Drechsler, Julian J. Holstein, and Guido H. Clever**

anie_202012425_sm_miscellaneous_information.pdf

Supporting Information

Table of Contents

1. Experimental section 2
1.1. Materials and measurements 2
1.2. Synthesis of ligands 2
1.2.1. Synthesis of 1,6-bis(3,6-bis(pyridin-3-ylethynyl)-9H-carbazol-9-yl)hexane (**L^{A1}**) 2
1.2.2. Synthesis of 1,4-bis(3,6-bis(pyridin-3-ylethynyl)-9H-carbazol-9-yl)benzene (**L^{A2}**) 3
1.3. Self-assembly and characterization of cages 4
1.3.1. Self-assembly of heteroleptic cage **$[Pd_2L^A{}_2L^B{}_2]^{4+}$** (**C**) in CD_3CN 4
1.3.2. Self-assembly of homoleptic cage **$[Pd_2(L^{A1})_2]^{4+}$** (**C1**) in DMSO-d_6 8
1.3.3. Self-assembly of homoleptic ring/tetrahedron mixture **$[Pd_3(L^B)_6]^{6+}$** (**R**)/**$[Pd_4(L^B)_8]^{8+}$** (**T**) in DMSO-d_6 11
1.3.4. Self-assembly with **L^{A1}** and **L^B** in CD_3CN 14
1.3.5. Self-assembly of heteroleptic pseudo-tetrahedron **$[Pd_3(L^{A1})(L^B)_4]^{6+}$** (**T1**) in DMSO-$d_6$ 15
1.3.6. Self-assembly of heteroleptic cage dimer **$[Pd_4(L^{A2})_2(L^B)_4]^{8+}$** (**D2**) in DMSO-$d_6$ 19
1.3.7. Self-assembly of heteroleptic cage dimer **$[Pd_4(L^{A2})_2(L^B)_4]^{8+}$** (**D2**) in DMF-$d_7$ 23
2. Cage-to-cage transformation 24
3. Host-Guest study 27
4. Ion Mobility Mass Spectrometry 28
5. Computational studies 30
6. X-ray Crystallography 32
6.1. Crystal structure of **D1** 33
6.1.1. Specific refinement details of **D1**. 33
6.2. Crystal structure of **D2** 35
6.2.1. Specific refinement details of **D2**. 35
6.3. Crystal structure of **P2** 37
6.3.1. Specific refinement details of **P2**. 37
7. References 39

1. Experimental section

1.1. Materials and measurements

Unless otherwise stated, all chemicals were obtained from commercial sources and used as received.

Compounds **2I-CBZ**,[1] **4I-CBZ**[2] (Scheme S1), **DCB**,[3] **4I-DCB**[4] (Scheme S2) and ligand $\mathbf{L^{A}}$[5], $\mathbf{L^{B}}$[6] were prepared according to literature procedures. Gel permeation chromatography (GPC) purification of ligands was performed on a JAI 9210-II NEXT GPC System with a JAIGEL HH-2/HH-1 column combination running with $CHCl_3$ (HPLC grade). High resolution Electrospray Ionization (ESI) mass spectra and trapped ion mobility data were recorded on Bruker ESI timsTOF and (electrospray ionization-trapped ion mobility-time of flight) Compact mass spectrometers. All samples were diluted with spectrum grade CH_3CN (1:10) prior to measurement. NMR experiments were measured on Bruker AVANCE III and NEO (500 or 600 MHz) spectrometers. Chemical shifts for ^{1}H and ^{13}C are reported in ppm with residual solvent as reference: acetonitrile (1.94 ppm for ^{1}H, 1.32 ppm for ^{13}C), DMSO (2.50 ppm for ^{1}H, 39.52 ppm for ^{13}C), DMF (2.75 ppm for ^{1}H, 29.76 ppm for ^{13}C). Abbreviations for signal multiplicity of ^{1}H NMR spectra are shown as following: s: singlet, d: doublet, t: triplet, dd: doublet of doublets; dt: doublet of triplets; m: multiplet, br: broad.

1.2. Synthesis of ligands

1.2.1. Synthesis of 1,6-bis(3,6-bis(pyridin-3-ylethynyl)-9H-carbazol-9-yl)hexane ($\mathbf{L^{A1}}$)

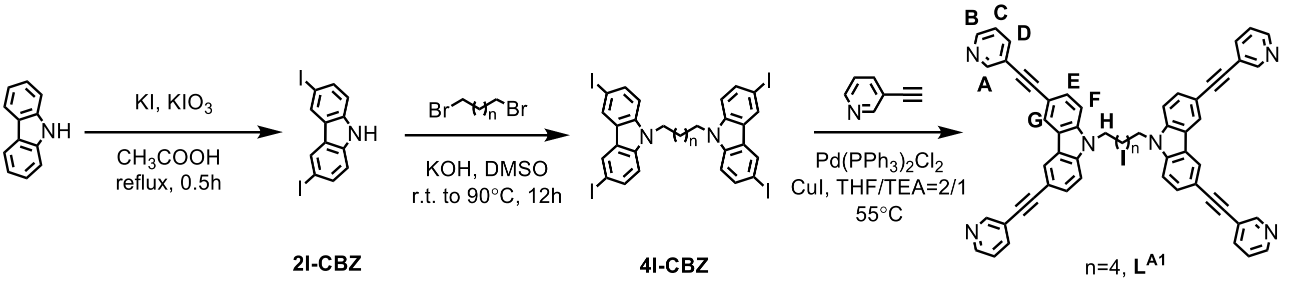

Scheme S1

Compound **4I-CBZ** (276 mg, 0.3 mmol, 1 eq.), 3-ethynylpyridine (247 mg, 2.4 mmol, 8 eq.), CuI (17 mg, 0.09 mmol, 0.03 eq.), triethylamine (11 mL), and anhydrous THF (22 mL) were added in a Schlenk tube. After the suspension was degassed (via freeze-thaw cycles) for three times, $Pd(PPh_3)Cl_2$ (32 mg, 0.045 mmol, 0.015 eq.) was added. The mixture was heated to r.t. and then to 55 °C for 20 h under the protection of a N_2 atmosphere. After the solvent was evaporated under reduced pressure, the crude product was purified by column chromatography (DCM:MeOH = 100:1 to 30:1) and then by GPC to yield the title compound as a pale yellowish brown solid (106 mg, 43%).

^{1}H NMR (500 MHz, 298K, DMSO-d_6) δ 8.76 (d, J = 2.2 Hz, 4H), 8.56 (dd, J = 4.9, 1.7 Hz, 4H), 8.53 (d, J = 1.5 Hz, 4H), 7.96 (dt, J = 7.8, 1.9 Hz, 4H), 7.71 – 7.61 (m, 8H), 7.48 – 7.41 (m, 4H), 4.38 (t, J = 7.1 Hz, 4H), 1.72 (t, J = 6.1 Hz, 4H), 1.32 (t, J = 6.7 Hz, 4H).

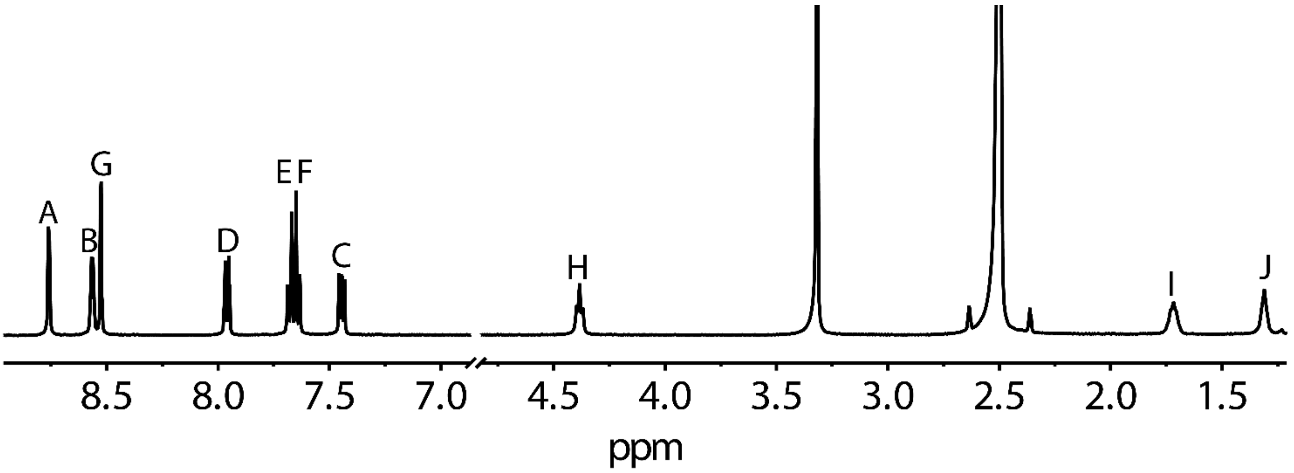

Figure S1. ^{1}H NMR spectrum (500 MHz, 298K, DMSO-d_6) of $\mathbf{L^{A1}}$.

^{13}C NMR (126 MHz, 298K, DMSO-d_6) δ 151.42, 148.56, 140.40, 138.24, 129.75, 124.50, 123.62, 121.80, 119.96, 112.29, 110.20, 93.83, 84.62, 42.37, 28.22, 25.89.

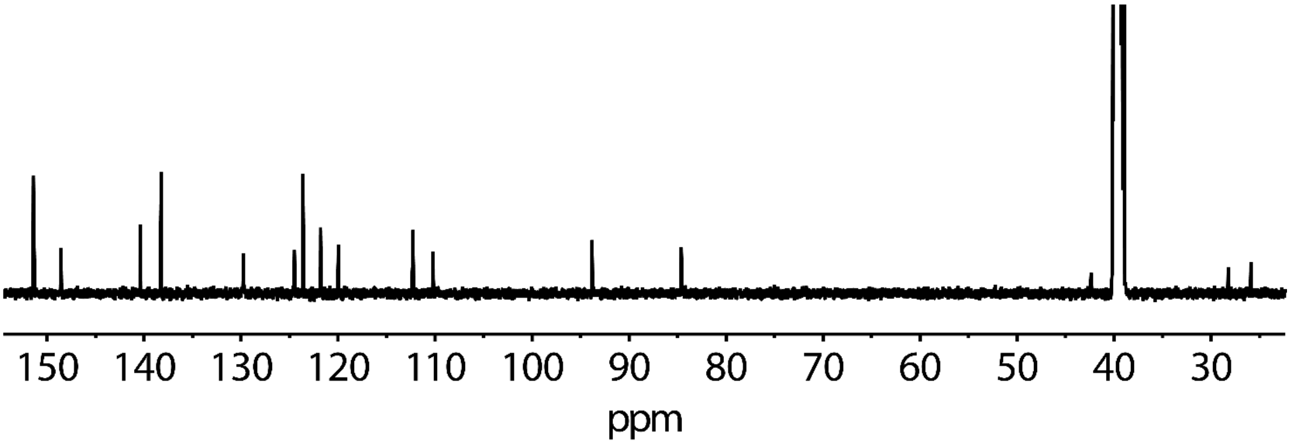

Figure S2. ^{13}C NMR spectrum (126 MHz, 298K, DMSO-d_6) of **L^{A1}**.

ESI-HRMS: *m/z*: calc. for $[M]^+(C_{58}H_{40}N_6)$: 820.3309, found: 820.3343.

*1.2.2. Synthesis of 1,4-bis(3,6-bis(pyridin-3-ylethynyl)-9H-carbazol-9-yl)benzene (**L^{A2}**)*

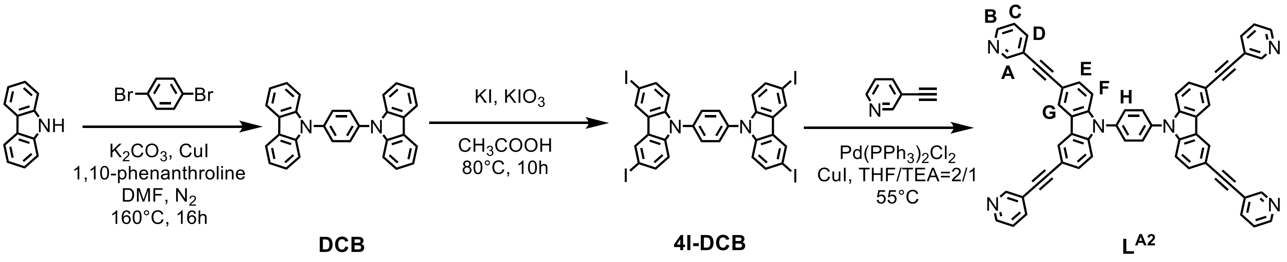

Scheme S2

Compound **4I-DCB** (183 mg, 0.2 mmol, 1 eq.), 3-ethynylpyridine (165 mg, 1.6 mmol, 8 eq.), CuI (12 mg, 0.06 mmol, 0.03 eq.), triethylamine (7 mL), and anhydrous THF (14 mL) were added in a Schlenk tube. After the suspension was degassed (via freeze-thaw cycles) for three times, $Pd(PPh_3)Cl_2$ (21 mg, 0.03 mmol, 0.015 eq.) was added. The mixture was heated to r.t. and then to 55 °C for 20 h under the protection of a N_2 atmosphere. After the solvent was evaporated under reduced pressure, the crude product was purified by column chromatography (DCM:MeOH = 100:1 to 30:1) and recrystallized from hot DMF to yield the title compound as a pale yellowish brown crystalline solid (80 mg, 49%).

^{1}H NMR (500 MHz, 298K, DMSO-d_6) δ 8.82 (d, J = 2.2 Hz, 4H), 8.70 (d, J = 1.6 Hz, 4H), 8.61 (dd, J = 4.8, 1.7 Hz, 4H), 8.07 – 7.98 (m, 8H), 7.78 (dd, J = 8.5, 1.6 Hz, 4H), 7.70 (d, J = 8.5 Hz, 4H), 7.50 (dd, J = 7.9, 4.8 Hz, 4H).

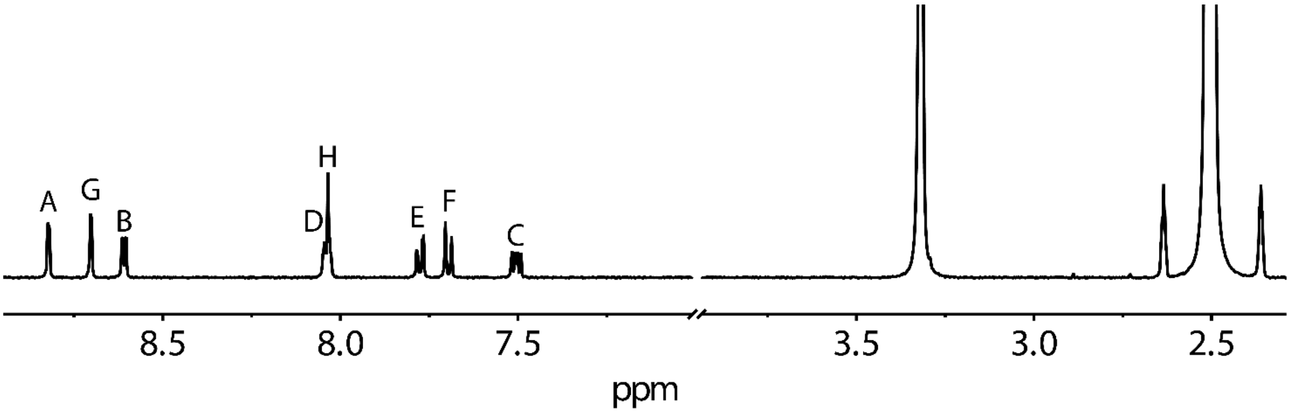

Figure S3. ^{1}H NMR spectrum (500 MHz, 298K, DMSO-d_6) of **L^{A2}**.

^{13}C NMR (151 MHz, 298K, DMSO-d_6) δ 151.51, 148.76, 140.60, 138.40, 130.45, 128.78, 124.78, 123.71, 123.22, 122.63, 119.84, 113.90, 110.82, 93.45, 85.11.

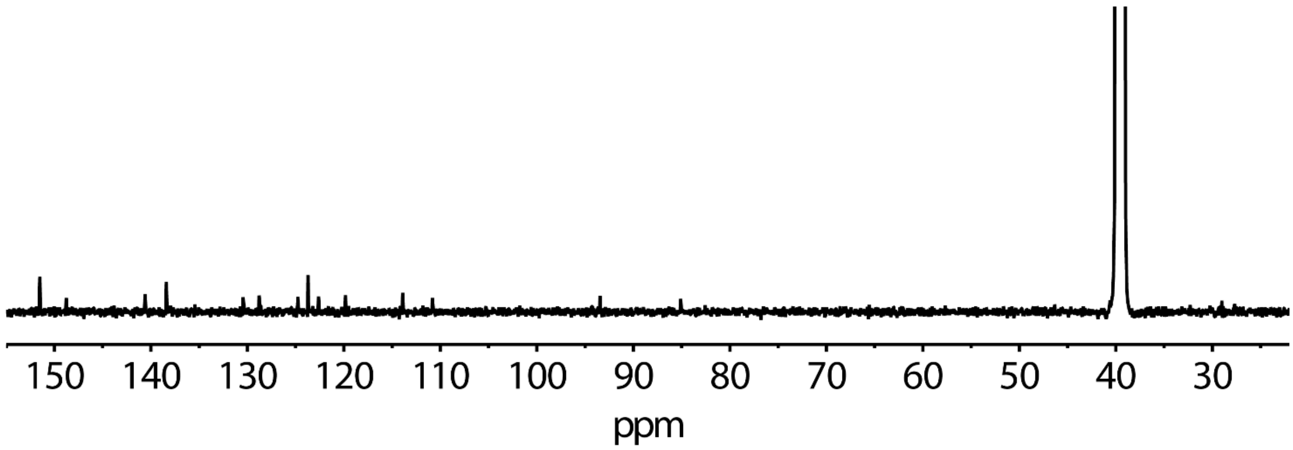

Figure S4. ^{13}C NMR spectrum (126 MHz, 298K, DMSO-d_6) of $\mathbf{L^{A2}}$.

ESI-HRMS: *m/z*: calc. for $[M]^+(C_{58}H_{32}N_6)$: 812.2683, found: 812.2684.

1.3. Self-assembly and characterization of cages

1.3.1. Self-assembly of heteroleptic cage *$[Pd_2L^A{}_2L^B{}_2]^{4+}$ (**C**) in CD_3CN*

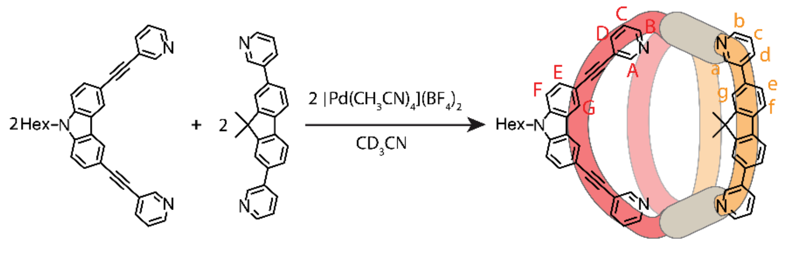

To a solution of $\mathbf{L^B}$ (7 mM, 0.7 µmol) in 100 µL CD_3CN and a suspension of $\mathbf{L^A}$ (0.32 mg, 0.7 µmol) in 353 µL CD_3CN was added a stock solution of $[Pd(CH_3CN)_4](BF_4)_2$ (47 µL, 15 mM/CD_3CN, 0.7 µmol). The mixture was heated at 80 °C for 8 h to give a 0.7 mM cage solution.

^{1}H NMR (500 MHz, CD_3CN) δ 9.59 (d, *J* = 2.0 Hz, 4H), 9.43 (d, *J* = 1.8 Hz, 4H), 9.09 (dd, *J* = 5.8, 1.4 Hz, 4H), 9.03 (dd, *J* = 5.8, 1.3 Hz, 4H), 8.29 (d, *J* = 1.6 Hz, 4H), 8.23 (dt, *J* = 7.9, 1.6 Hz, 4H), 8.13 (dt, *J* = 8.0, 1.6 Hz, 4H), 7.95 (d, *J* = 7.7 Hz, 4H), 7.73 (dd, *J* = 8.6, 1.6 Hz, 4H), 7.66 (ddd, *J* = 8.0, 5.8, 3.7 Hz, 8H), 7.61 (d, *J* = 1.7 Hz, 4H), 7.60 – 7.56 (m, 4H), 7.49 (dd, *J* = 7.7, 1.7 Hz, 4H), 4.34 (t, *J* = 7.2 Hz, 4H), 1.80 – 1.74 (m, 4H), 1.67 (s, 6H), 1.31 (s, 6H), 1.26 – 1.13 (m, 12H), 0.76 (t, *J* = 7.1 Hz, 6H).

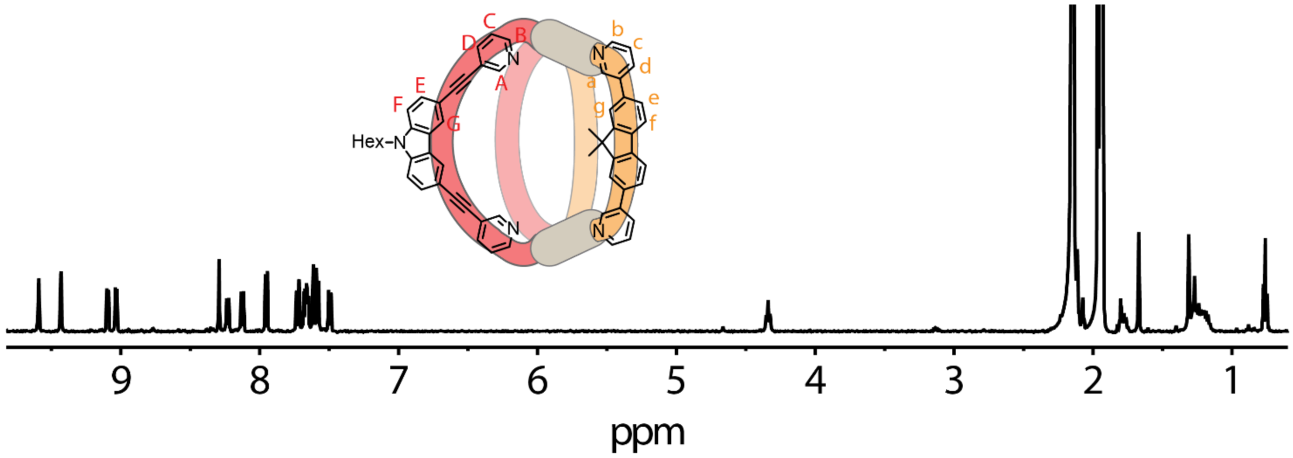

Figure S5. ^{1}H NMR spectrum (500 MHz, CD_3CN) of heteroleptic cage **C**.

^{13}C NMR (151 MHz, CD_3CN) δ 156.78, 153.31, 150.42, 150.20, 149.97, 143.71, 143.12, 142.34, 140.66, 139.73, 136.02, 132.26, 128.99, 128.38, 128.23, 125.41, 124.41, 123.05, 122.84, 122.69, 112.99, 111.50, 98.10, 83.41, 48.30, 44.02, 32.09, 29.97, 29.40, 27.28, 25.72, 23.15, 14.14.

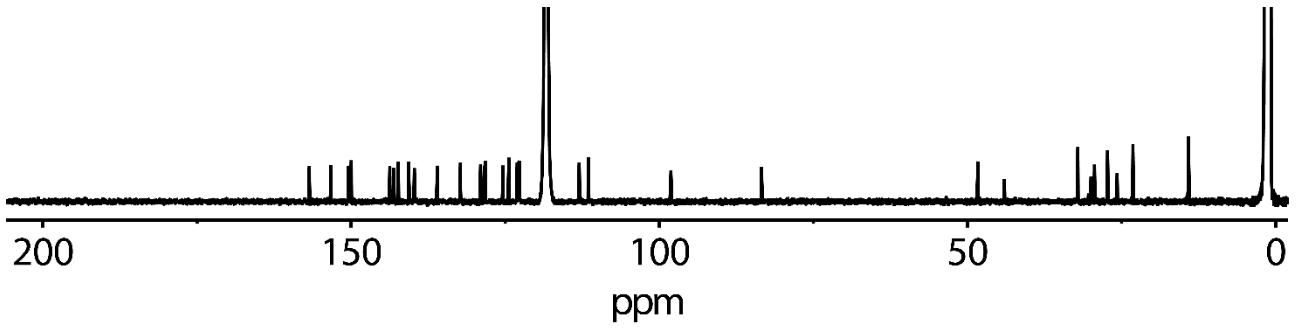

Figure S6. ^{13}C NMR spectrum (151 MHz, CD_3CN) of heteroleptic cage **C**.

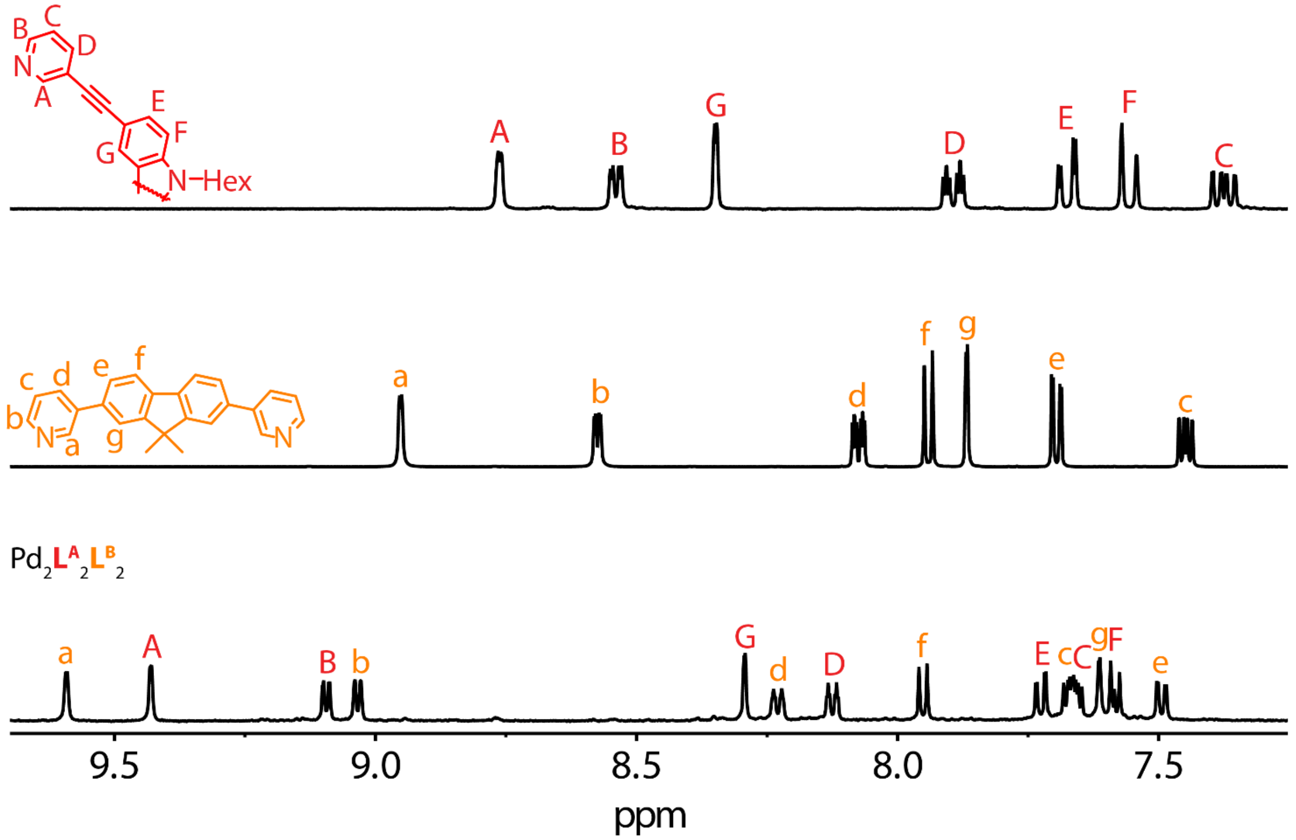

Figure S7. Partial ^{1}H NMR spectrum (500 MHz, CD_3CN) comparison of ligand $\mathbf{L^A}$ (top), ligand $\mathbf{L^B}$ (middle), and heteroleptic cage **C** (bottom).

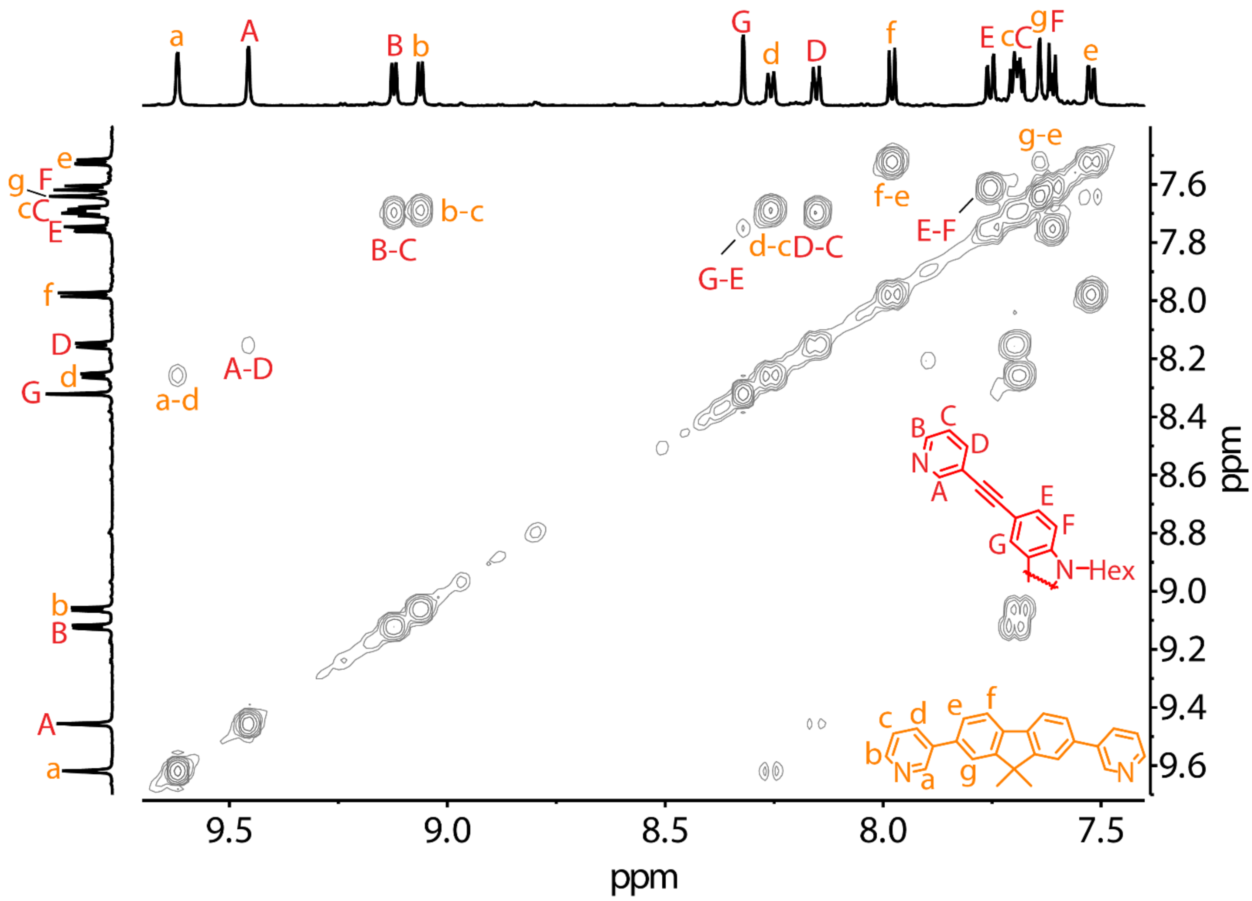

Figure S8. Partial ^{1}H–^{1}H COSY spectrum (500 MHz, CD_3CN) of heteroleptic cage **C**.

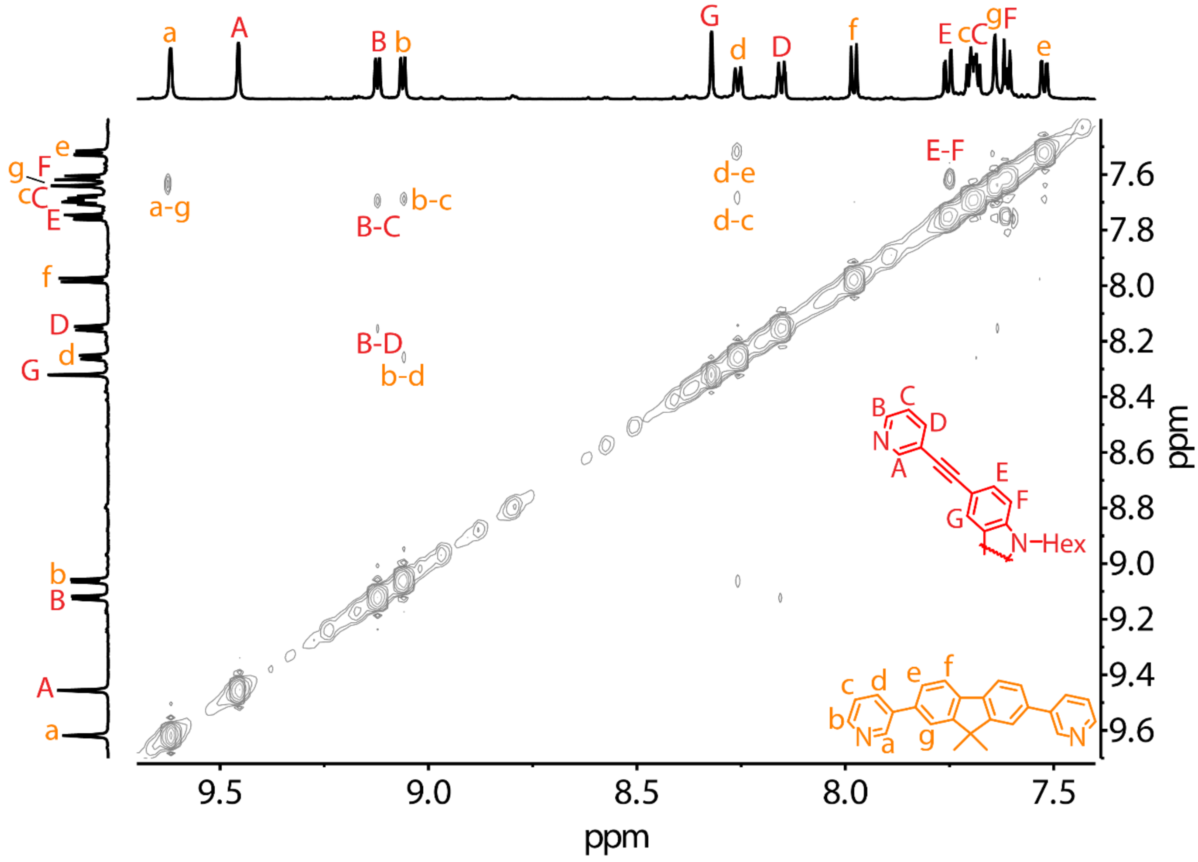

Figure S9. Partial ^{1}H–^{1}H NOESY spectrum (500 MHz, CD_3CN) of heteroleptic cage **C**.

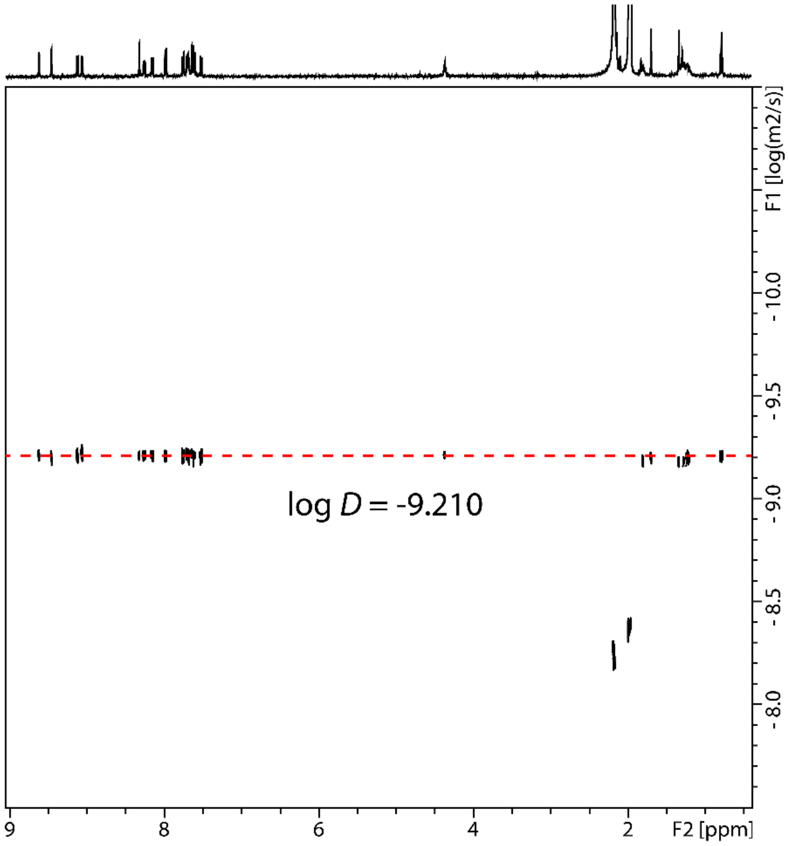

Figure S10. ^{1}H DOSY spectrum (500 MHz, CD_3CN) of heteroleptic cage **C**. Diffusion coefficient for **C**: D = 6.17 x 10^{-10} m^2s^{-1}, log *D* = −9.210, r = 10.6 Å.

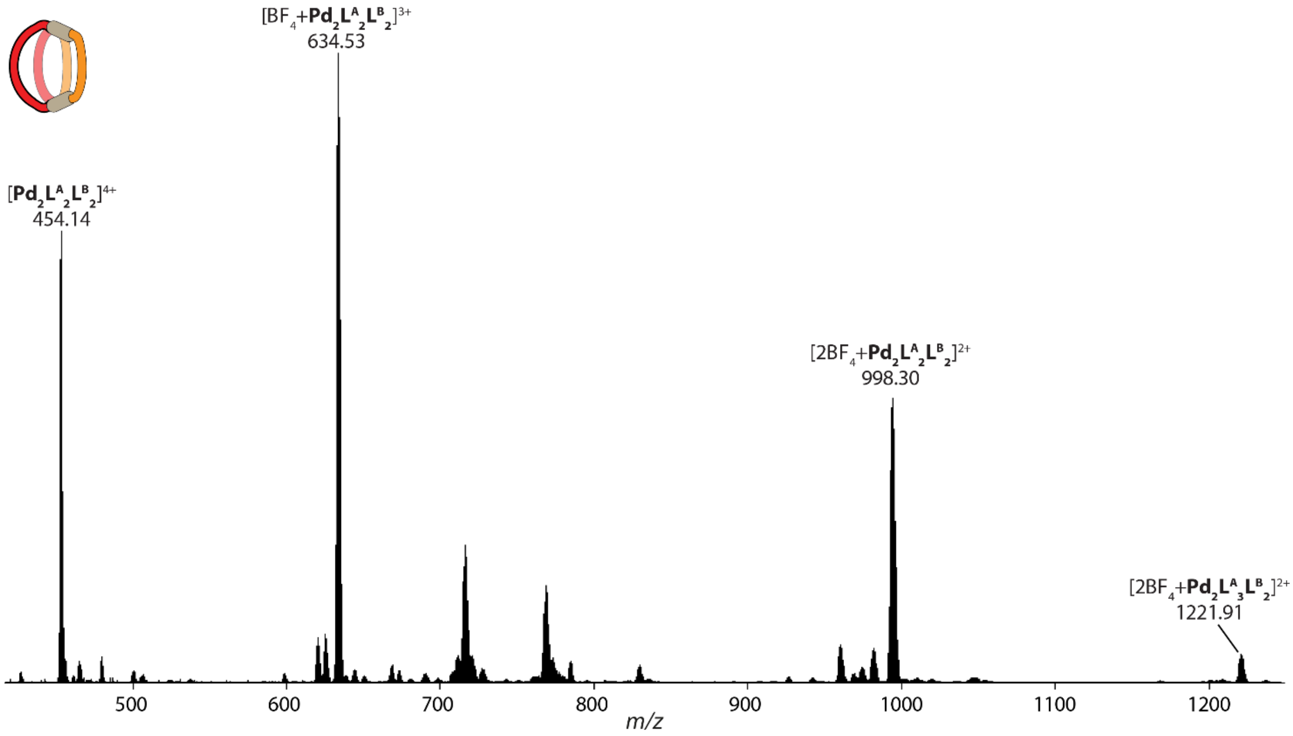

Figure S11. HR-ESI mass spectrum of heteroleptic cage **C**.

1.3.2. Self-assembly of homoleptic cage ***[Pd$_2$(L^{A1})$_2$]$^{4+}$*** *(**C1**) in DMSO-d$_6$*

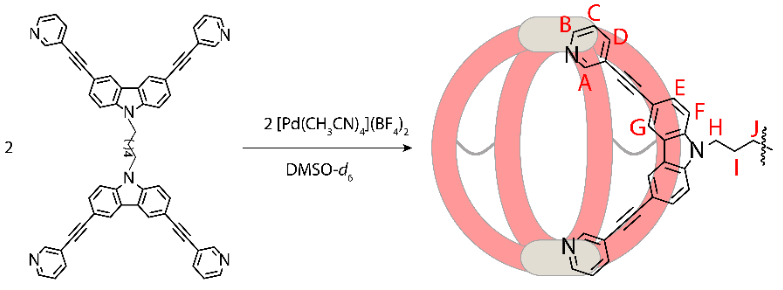

C1

To a solution of **L^{A1}** (0.54 mg, 0.7 µmol) in 465 µL DMSO-d_6 was added a stock solution of [Pd(CH$_3$CN)$_4$](BF$_4$)$_2$ (35 µL, 20 mM/DMSO-d_6, 0.7 µmol). The mixture was kept at r.t. for 3 h to give a 0.7 mM solution of cage **C1**.

^{1}H NMR (500 MHz, 298K, DMSO-d_6) δ 9.70 (s, 4H), 9.62 (s, 4H), 9.22 (dd, J = 11.4, 5.7 Hz, 8H), 8.49 (d, J = 1.5 Hz, 4H), 8.47 (d, J = 1.3 Hz, 4H), 8.30 (d, J = 7.7 Hz, 8H), 8.08 (d, J = 8.7 Hz, 4H), 7.96 (d, J = 8.7 Hz, 8H), 7.86 (d, J = 8.5 Hz, 4H), 7.82 (q, J = 4.7, 2.5 Hz, 8H), 4.69 (s, 4H), 4.60 (s, 4H), 1.70 (s, 8H), 1.62 (d, J = 6.7 Hz, 4H), 1.47 (s, 4H).

The observed twofold splitting of signals can be explained as follows: the lantern-shaped cage adopts an overall helical structure, governed by the [Pd(pyridine)$_4$] propellers. Unlike with untethered homoleptic [Pd$_2$L$_4$] cages, flipping between both helical cage enantiomers seems to be slowed down by the backbone bridges, leading to diastereotopic splitting of all protons coming in pairs (which – owing to the dimeric character of the ligands – is true both for all aromatic and aliphatic positions).

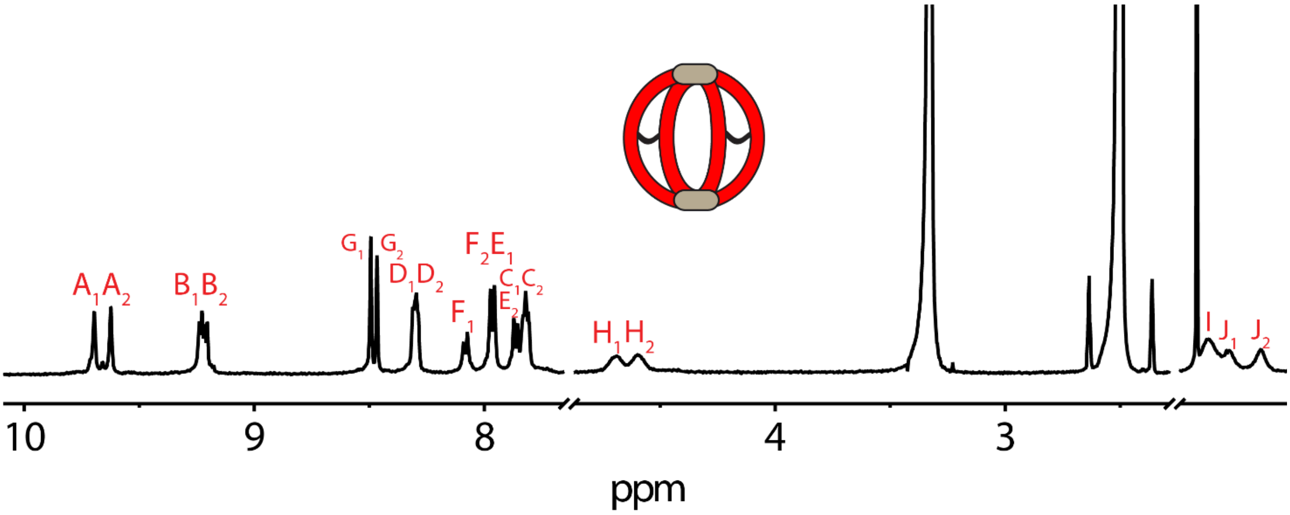

Figure S12. ^{1}H NMR spectrum (500 MHz, 298K, DMSO-d_6) of homoleptic cage **C1**.

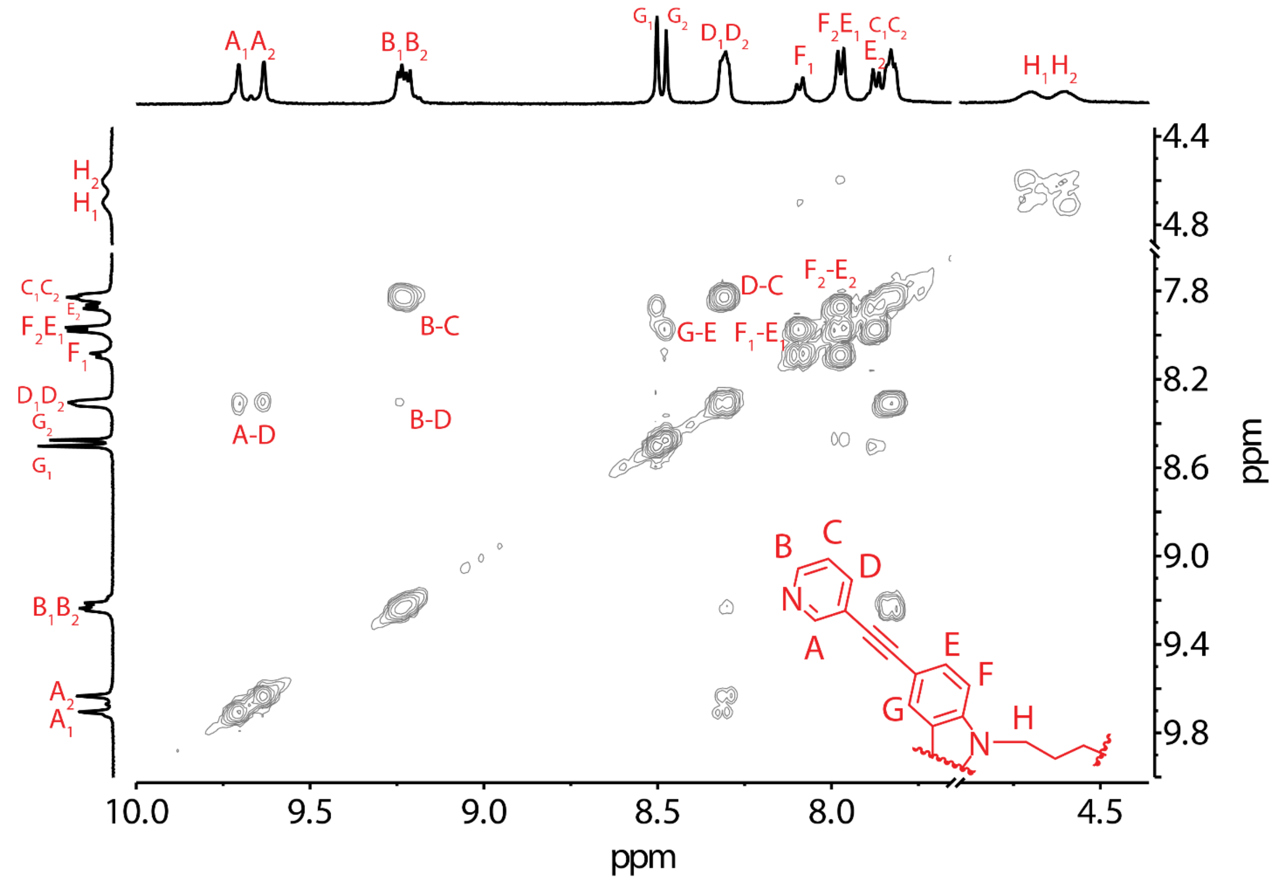

Figure S13. Partial ^1H–^1H COSY spectrum (500 MHz, 298K, DMSO-d_6) of homoleptic cage **C1**.

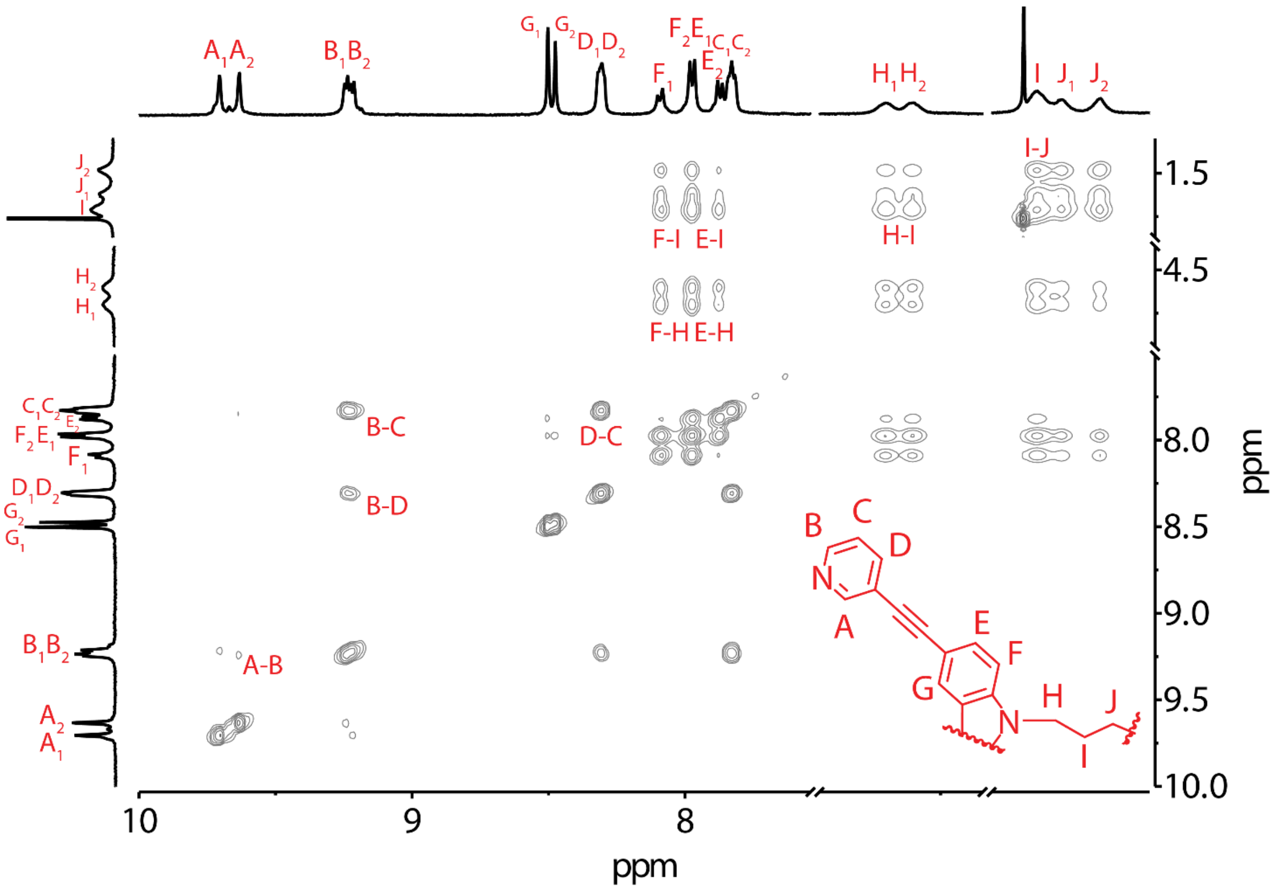

Figure S14. Partial ^1H–^1H NOESY spectrum (500 MHz, 298K, DMSO-d_6) of homoleptic cage **C1**.

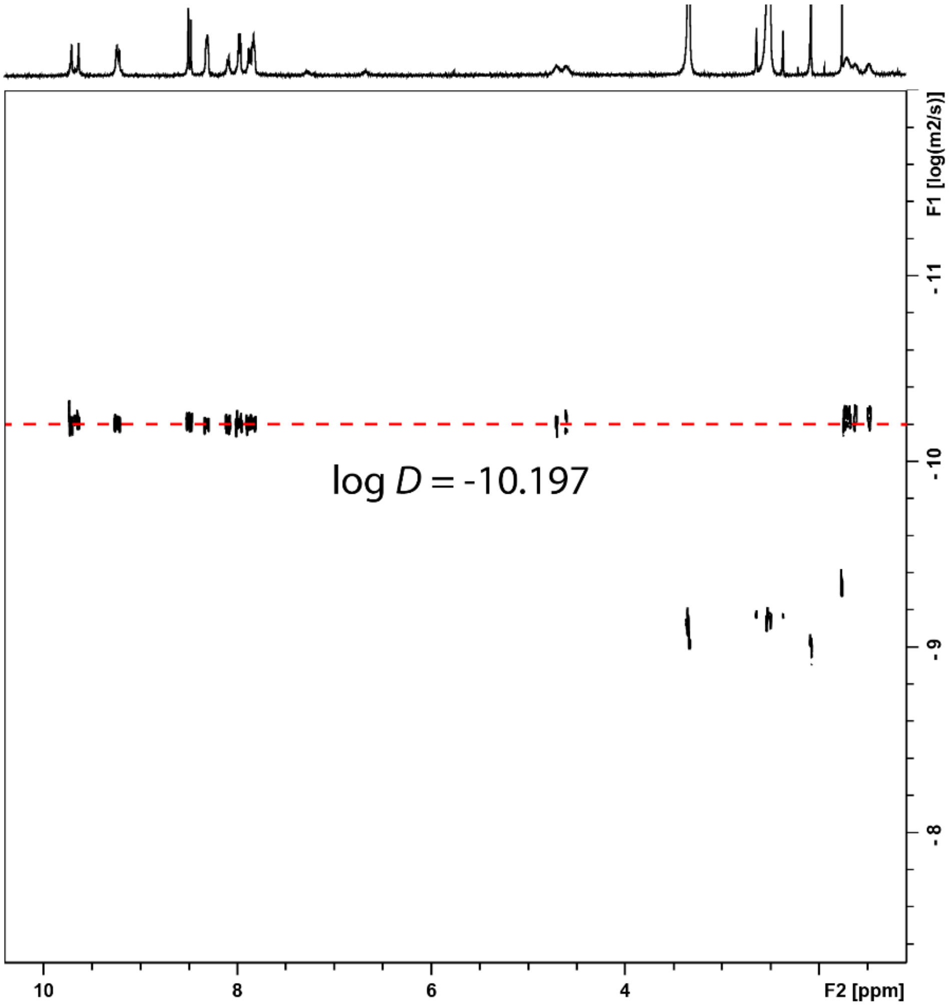

Figure S15. ^{1}H DOSY NMR spectrum (500 MHz, 298K, DMSO-d_6) of homoleptic cage **C1**. Diffusion coefficient D = 6.353 x 10^{-11} m^2s^{-1}, log D = −10.197, r = 17.2 Å.

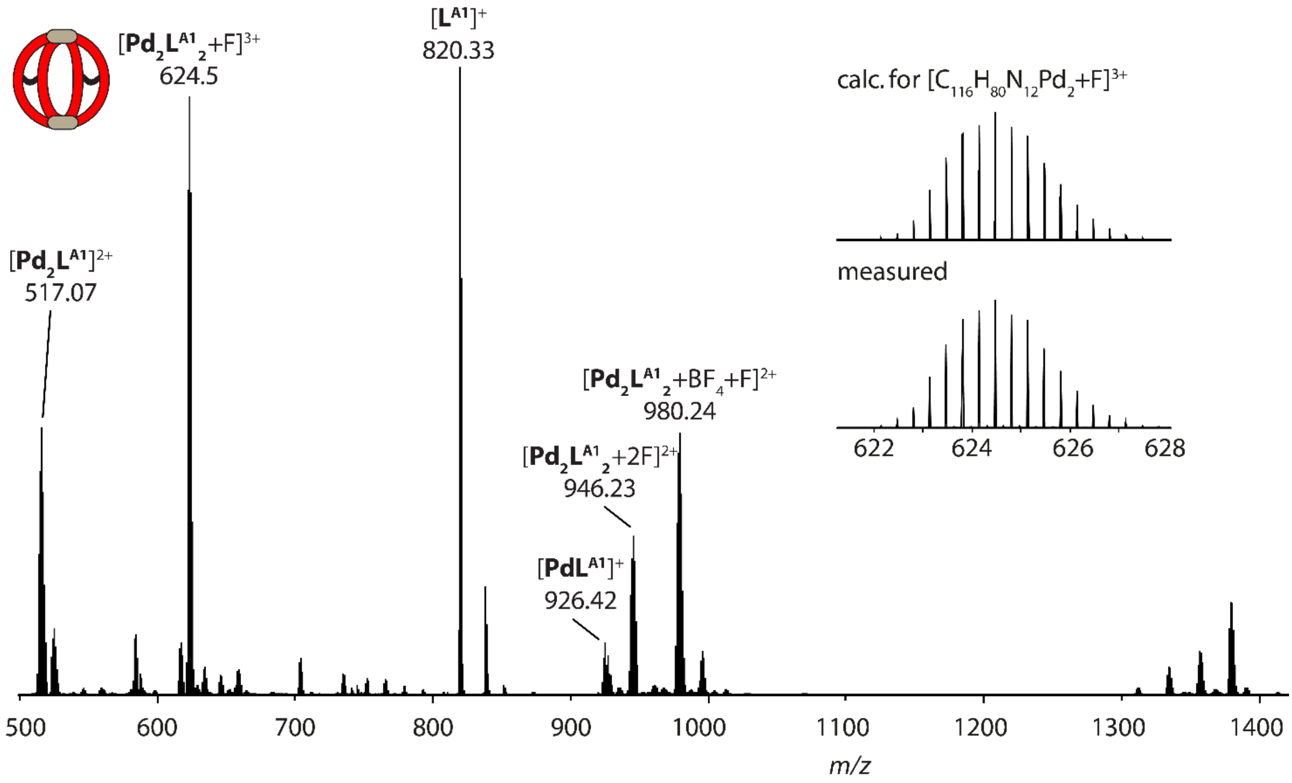

Figure S16. HR-ESI mass spectrum of homoleptic cage **C1**.

1.3.3. Self-assembly of homoleptic ring/tetrahedron mixture ***[Pd$_3$(L^B)$_6$]$^{6+}$*** *(**R**)/**[Pd$_4$(L^B)$_8$]$^{8+}$** (**T**) in DMSO-d_6*

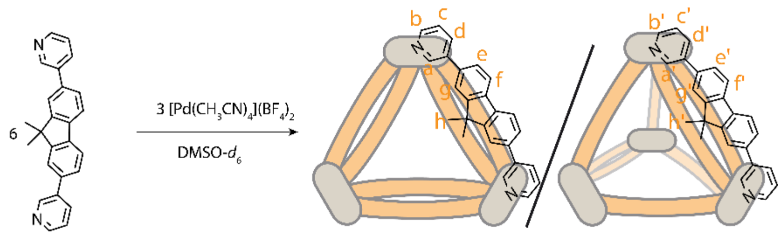

To a solution of **L^B** (7 mM, 2.1 µmol) in 447 µL DMSO-d_6 was added a stock solution of [Pd(CH$_3$CN)$_4$](BF$_4$)$_2$ (53 µL, 20 mM/DMSO-d_6, 1.05 µmol). The mixture was kept at r.t. for 3 h to give a mixture of homoleptic ring/tetrahedron (**R**/**T**).

^{1}H NMR (500 MHz, 298K, DMSO-d_6) δ 9.96 (s, 12H), 9.92 (s, 8H), 9.80 (s, 8H), 9.48 (d, J = 5.6 Hz, 8H), 9.34 (d, J = 5.7 Hz, 12H), 9.26 (d, J = 5.6 Hz, 8H), 8.61 (d, J = 8.3 Hz, 8H), 8.45 (d, J = 8.1 Hz, 12H), 8.38 (d, J = 6.6 Hz, 28H), 8.20 (d, J = 8.0 Hz, 8H), 8.09 (d, J = 17.2 Hz, 16H), 7.98 – 7.90 (m, 20H), 7.88 (dd, J = 8.0, 5.6 Hz, 12H), 7.81 (t, J = 7.4 Hz, 16H), 7.52 (s, 20H), 1.49 (s, 20H), 1.31 (s, 20H), 1.25 (s, 12H), 0.95 (s, 20H), 0.89 (s, 12H).

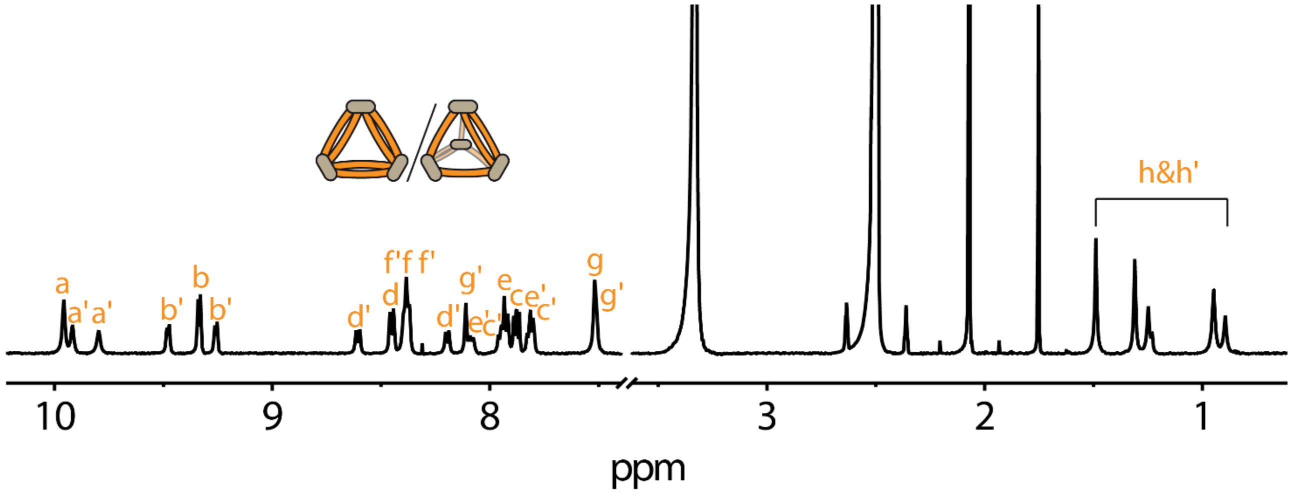

Figure S17. ^{1}H NMR spectrum (500 MHz, 298K, DMSO-d_6) of homoleptic ring/tetrahedron (**R/T**) mixture.

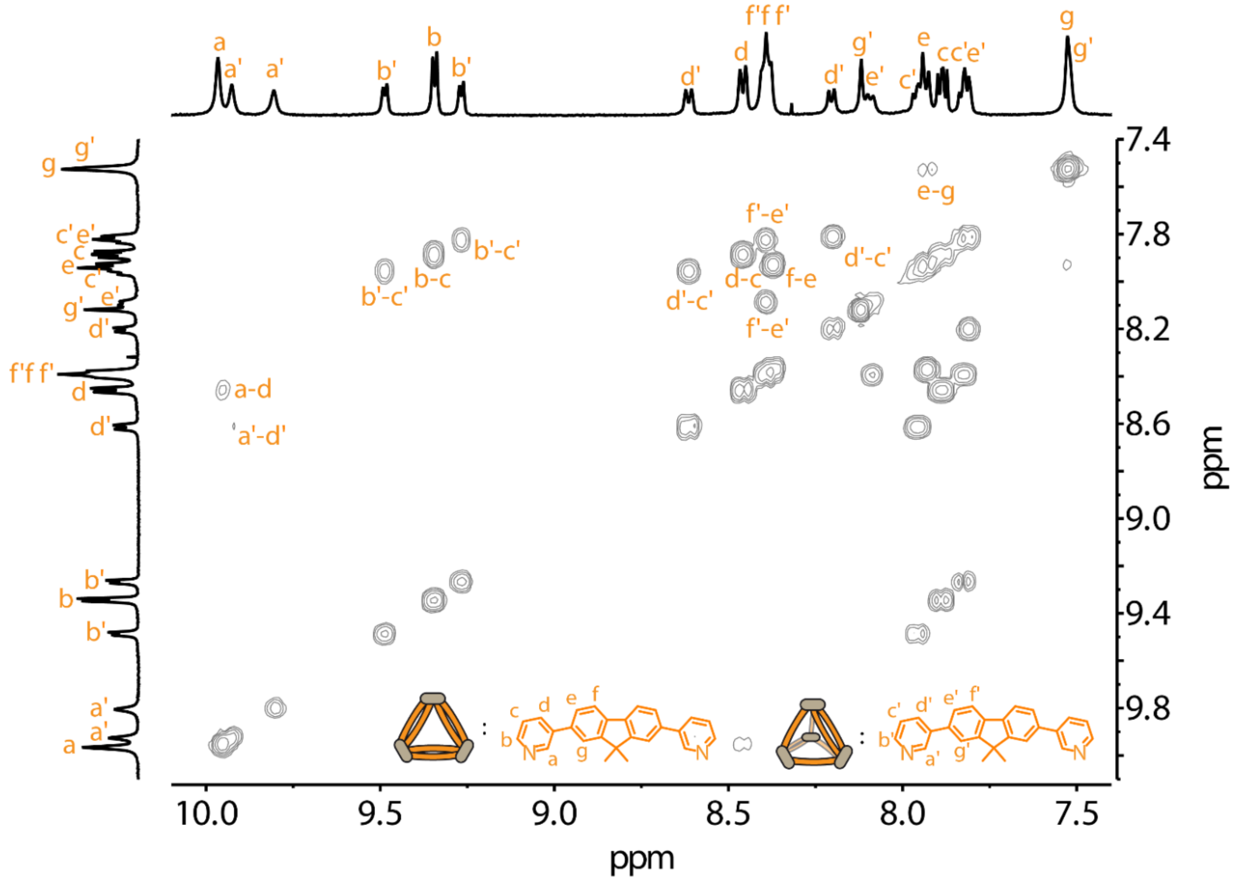

Figure S18. Partial ^{1}H–^{1}H COSY spectrum (500 MHz, 298K, DMSO-d_6) of homoleptic ring/tetrahedron (**R/T**) mixture.

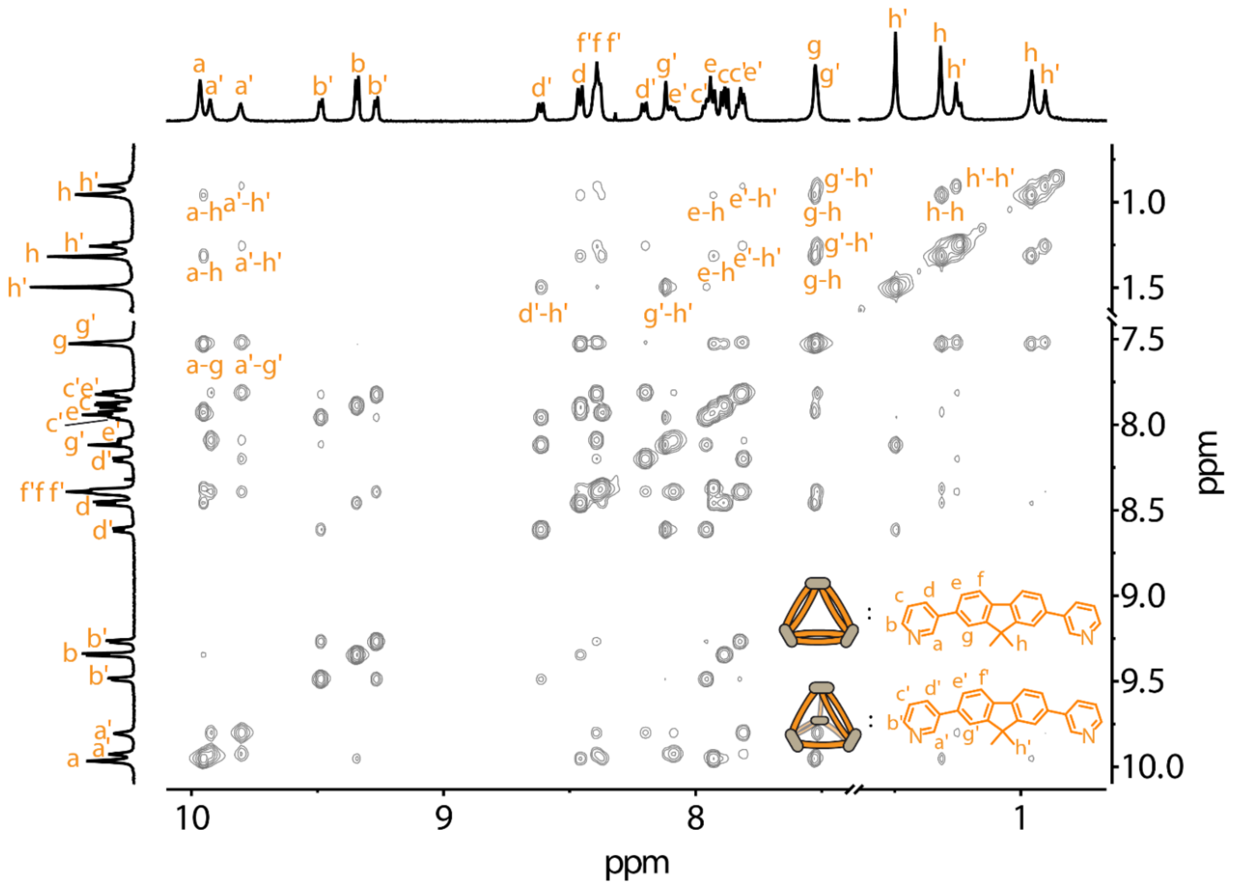

Figure S19. Partial $^1H–^1H$ NOESY spectrum (500 MHz, 298K, DMSO-d_6) of homoleptic ring/tetrahedron (**R/T**) mixture.

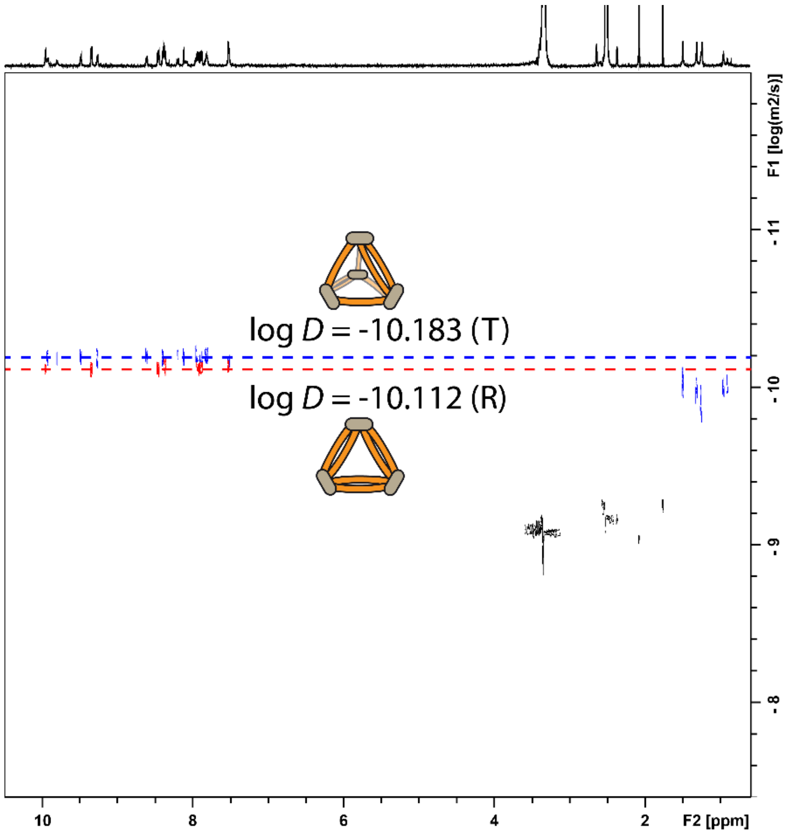

Figure S20. ^1H DOSY spectrum (500 MHz, 298K, DMSO-d_6) of homoleptic ring/tetrahedron (**R/T**) mixture. Diffusion coefficient for ring **R**, D = 7.731 x 10^{-11} m^2s^{-1}, log *D* = −10.112, r = 14.2 Å, for tetrahedron **T** D = 6.561 x 10^{-11} m^2s^{-1}, log *D* = −10.183, r = 16.7 Å.

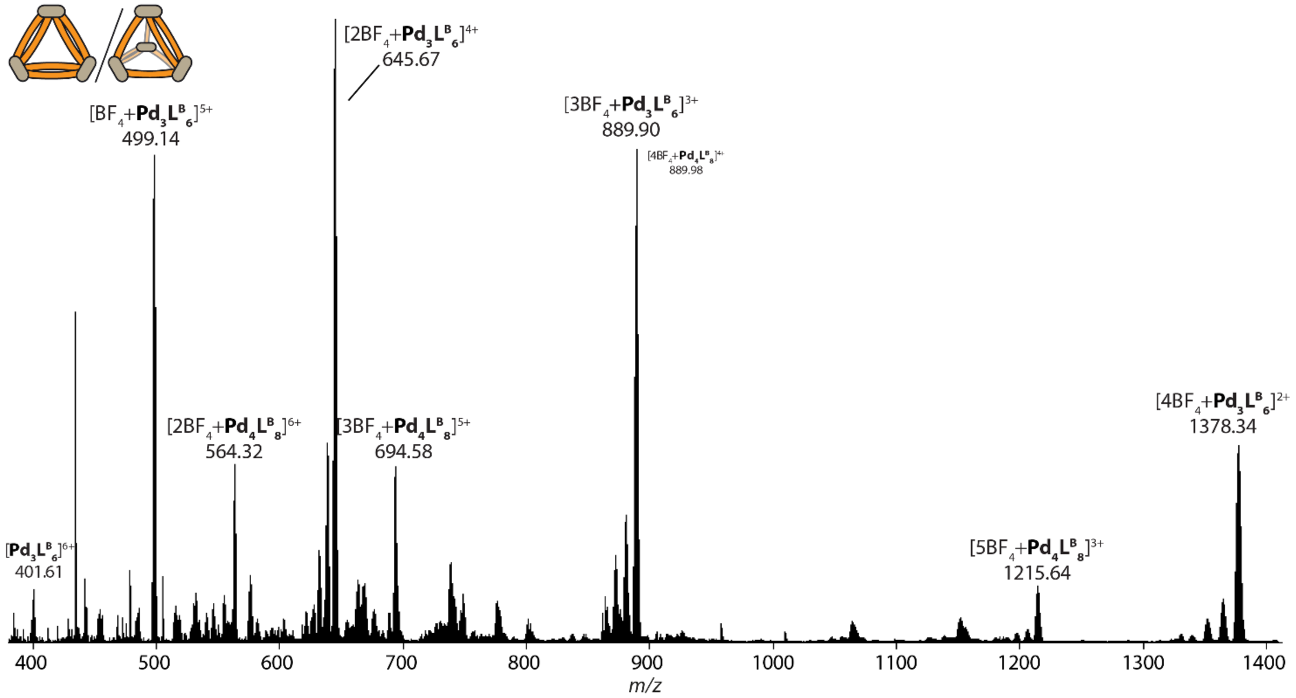

Figure S21. HR-ESI mass spectrum of homoleptic ring/tetrahedron (**R/T**) mixture.

1.3.4. Self-assembly with $\mathbf{L^{A1}}$ *and* $\mathbf{L^{B}}$ *in* CD_3CN

$\mathbf{L^{A1}}$ (0.64 mg, 0.78 µmol) and $\mathbf{L^{B}}$ (1.08 mg, 3.12 µmol) were combined in a small vial, 545 µL CD_3CN and a stock solution of $[Pd(CH_3CN)_4](BF_4)_2$ (155 µL, 15 mM/CD_3CN, 2.34 µmol) were added. The mixture was heated at 80 °C overnight to give a convoluted mixture of five different self-assembled structures.

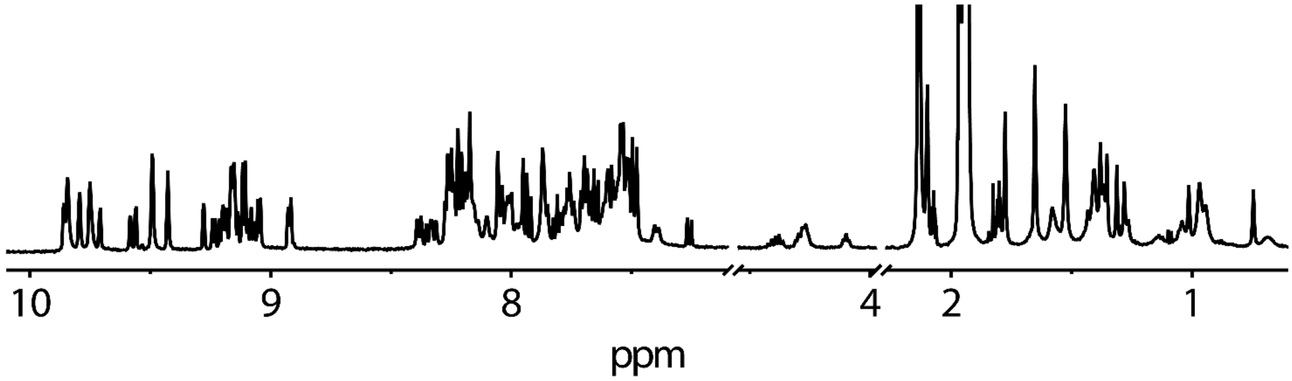

Figure S22. ^{1}H NMR spectrum (500 MHz, 298K, CD_3CN) of the Pd-mediated assembly of $\mathbf{L^{A1}}$ with $\mathbf{L^B}$.

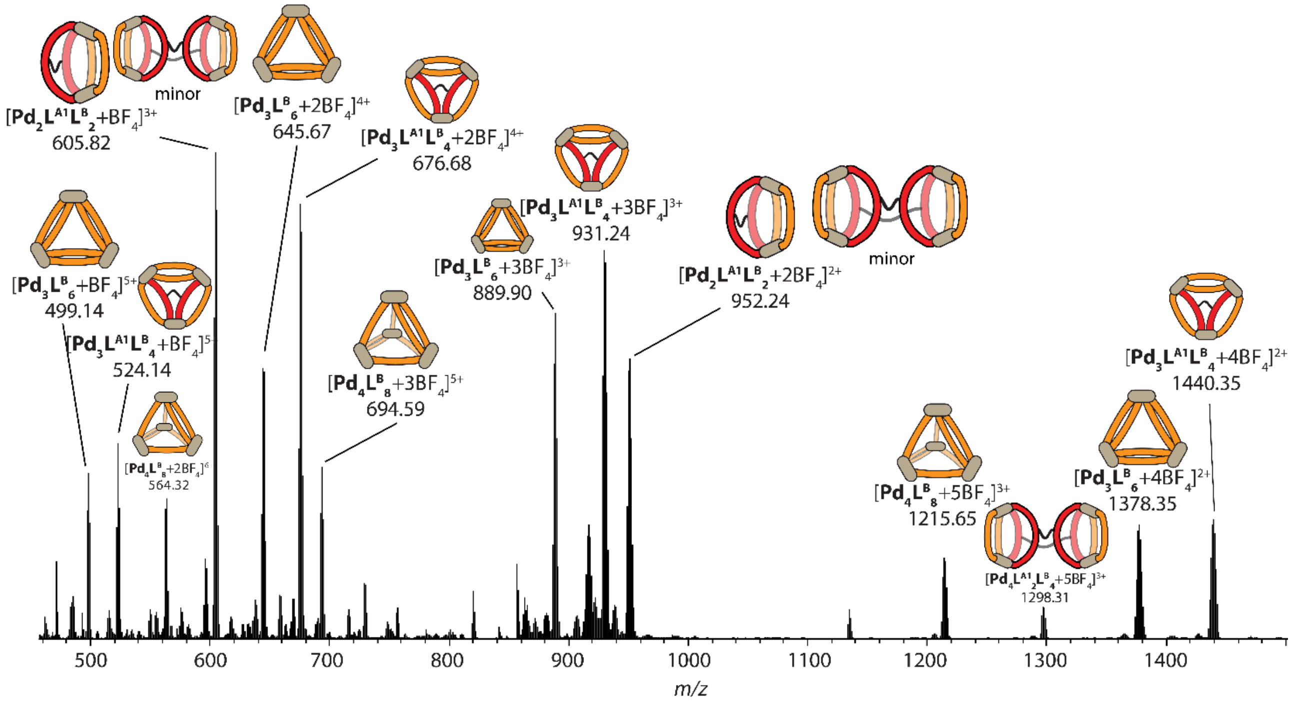

Figure S23. HR-ESI mass spectrum of the Pd-mediated assembly of $\mathbf{L^{A1}}$ with $\mathbf{L^B}$.

1.3.5. Self-assembly of heteroleptic pseudo-tetrahedron $\mathbf{[Pd_3(L^{A1})(L^B)_4]^{6+}}$ *(**T1**) in DMSO-*d_6

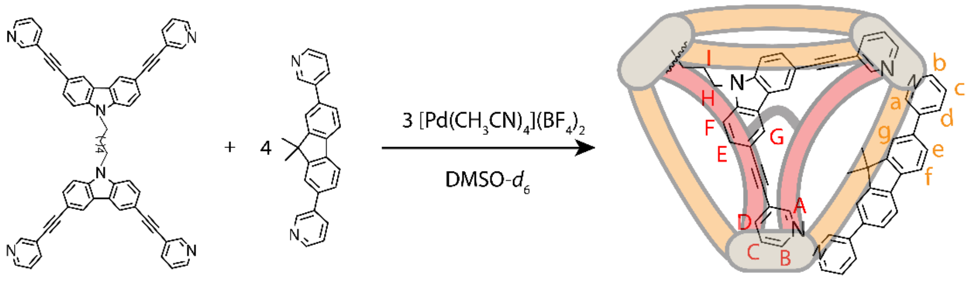

$\mathbf{L^{A1}}$ $\mathbf{L^B}$ **T1**

A solution of $\mathbf{L^{A1}}$ (0.29 mg, 0.35 µmol) in 247 µL DMSO-d_6 and a solution of $\mathbf{L^B}$ (1.4 µmol, 7 mM) in 200 µL DMSO-d_6 was combined. To this solution, a stock solution of $[Pd(CH_3CN)_4](BF_4)_2$ (53 µL, 20 mM/DMSO-d_6, 1.05 µmol) was added. The mixture was heated at 80 °C for 8 h to give a 0.7 mM solution of heteroleptic pseudo-tetrahedron **T1**.

^{1}**H NMR** (500 MHz, 298K, DMSO-d_6) δ 10.23 (s, 2H), 9.93 (s, 2H), 9.80 (s, 2H), 9.71 (s, 2H), 9.60 (s, 2H), 9.51 (s, 2H), 9.50 – 9.43 (m, 6H), 9.26 (d, J = 5.5 Hz, 2H), 9.21 (d, J = 5.6 Hz, 2H), 9.06 (d, J = 5.7 Hz, 2H), 8.78 (d, J = 7.9 Hz, 2H), 8.59 (d, J = 8.0 Hz, 4H), 8.54 (d, J = 8.0 Hz, 2H), 8.51 – 8.45 (dd, J = 12.1, 7.9 Hz, 4H), 8.42 (s, 2H), 8.33 (d, J = 7.8 Hz, 2H), 8.28 (d, J = 8.0 Hz, 2H), 8.27 (s, 2H), 8.26 – 8.19 (m, 6H), 8.09 (s, 2H), 8.03 (s, 2H), 8.01 – 7.99 (m, 2H), 7.99 – 7.92 (m, 8H), 7.86 (td, J = 8.2, 5.8 Hz, 4H), 7.83 – 7.74 (m, 2H), 7.75 – 7.66 (m, 6H), 7.61 (d, J = 8.7 Hz, 2H), 7.58 – 7.46 (m, 4H), 6.94 (s, 2H), 4.40

(t, J = 7.1 Hz, 2H), 4.15 (t, J = 7.1 Hz, 2H), 1.80 (b, 2H)1.72 (s, 6H), 1.63 (s, 6H), 1.53 (b, 2H), 1.47 (s, 3H), 1.43 (s, 3H), 1.21 (b, 4H), 1.13 (s, 3H), 0.17 (s, 3H).

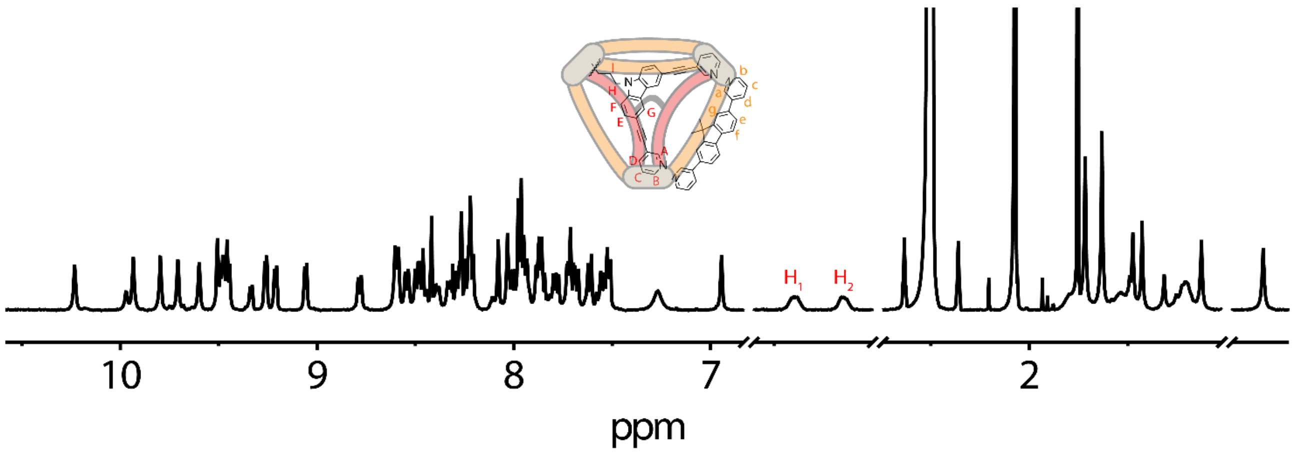

Figure S24. ^{1}H NMR spectrum (500 MHz, 298K, DMSO-d_6) of heteroleptic pseudo-tetrahedron **T1**.

^{13}C NMR (151 MHz, 298K, DMF-d7) δ 172.02, 156.21, 155.92, 155.80, 155.63, 154.84, 153.24, 152.82, 152.01, 151.44, 151.20, 150.60, 150.23, 150.03, 149.88, 149.81, 148.93, 148.31, 148.09, 143.93, 143.66, 141.79, 141.03, 140.89, 140.85, 140.74, 140.39, 140.24, 140.07, 139.82, 139.70, 139.25, 138.77, 138.55, 135.45, 135.14, 134.77, 134.56, 130.72, 130.55, 128.43, 128.25, 128.09, 127.96, 127.80, 127.45, 127.26, 126.93, 126.54, 124.82, 124.26, 123.97, 123.89, 123.21, 122.88, 122.68, 122.55, 122.41, 122.23, 121.95, 121.38, 120.99, 117.91, 111.98, 111.95, 111.15, 110.82, 96.38, 96.26, 81.44, 81.02, 48.18, 47.82, 47.75, 47.25, 42.13, 27.04, 26.73, 26.53, 26.37, 25.90, 24.60, 24.14, 23.53, 22.17.

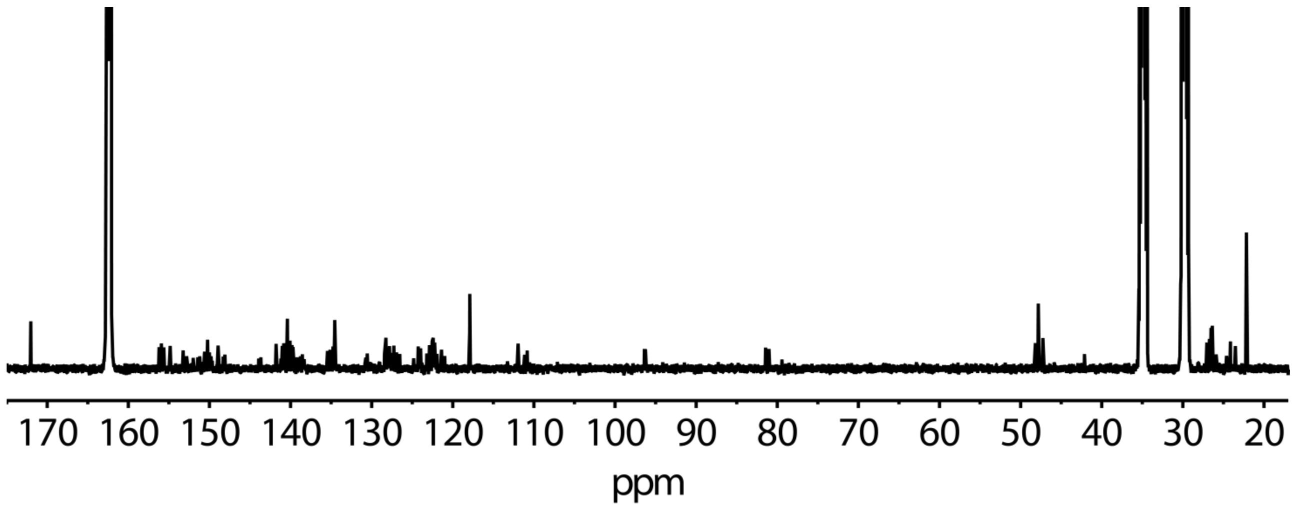

Figure S25. ^{13}C NMR spectrum (151 MHz, 298K, DMF-d_7) of heteroleptic pseudo-tetrahedron **T1**.

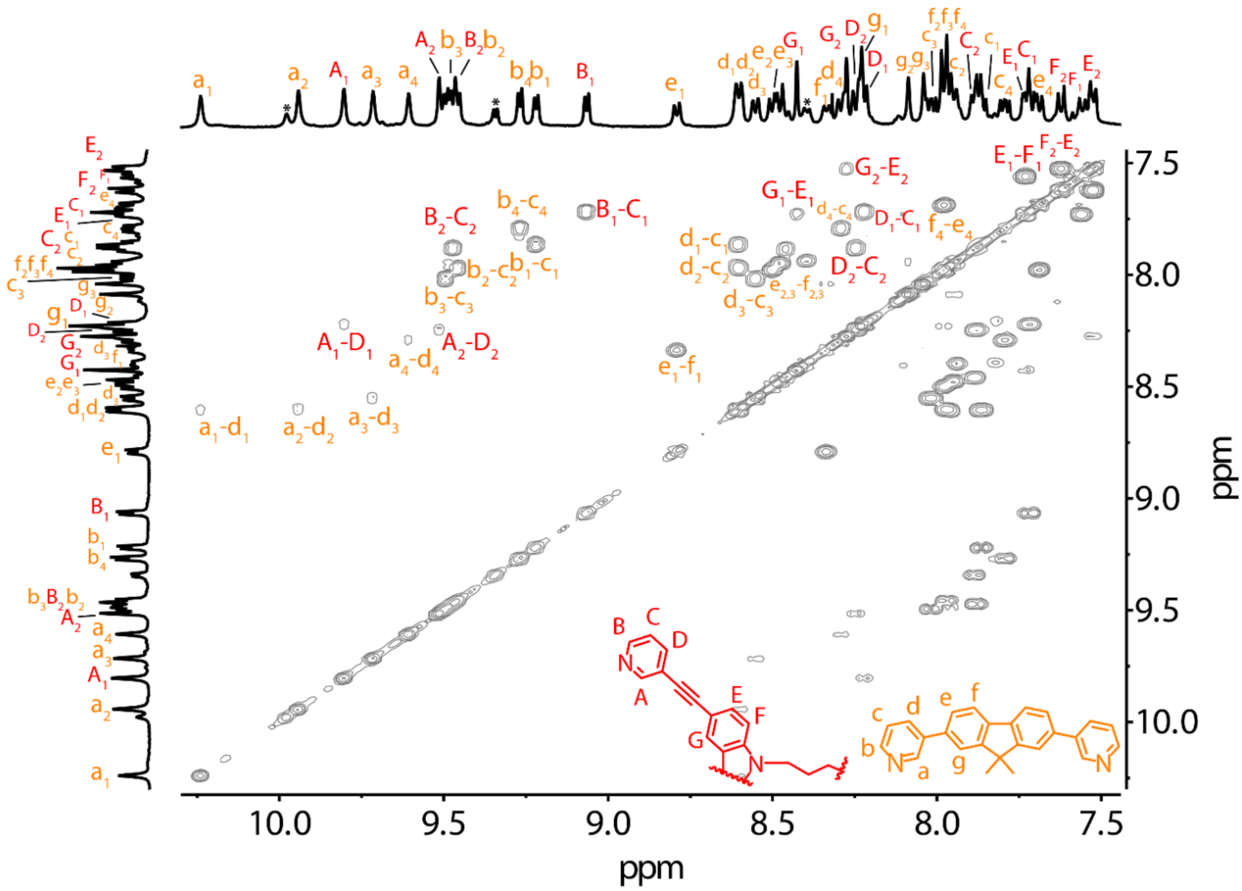

Figure S26. Partial ^1H–^1H COSY spectrum (500 MHz, 298K, DMSO-d_6) of heteroleptic pseudo-tetrahedron **T1**.

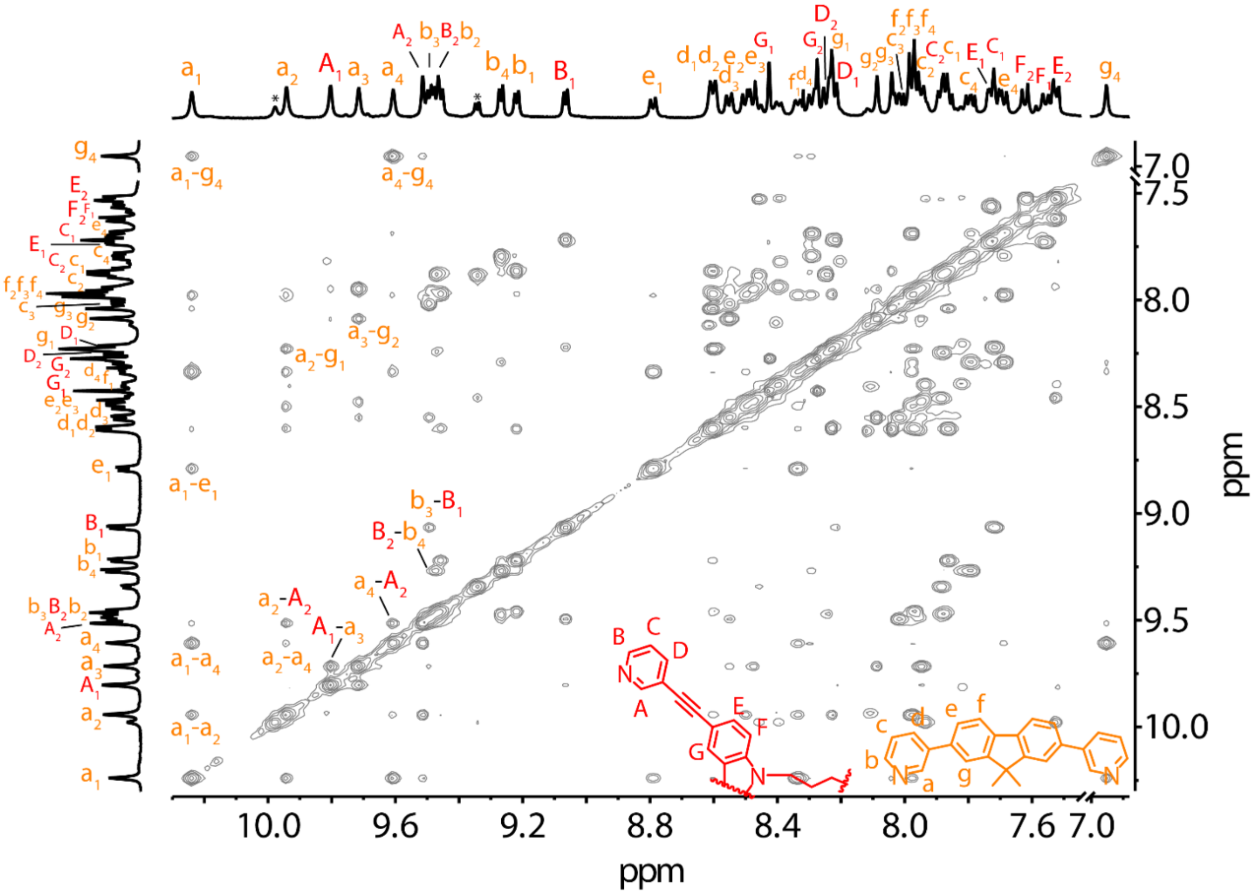

Figure S27. Partial ^1H–^1H NOESY spectrum (500 MHz, 298K, DMSO-d_6) of heteroleptic pseudo-tetrahedron **T1**.

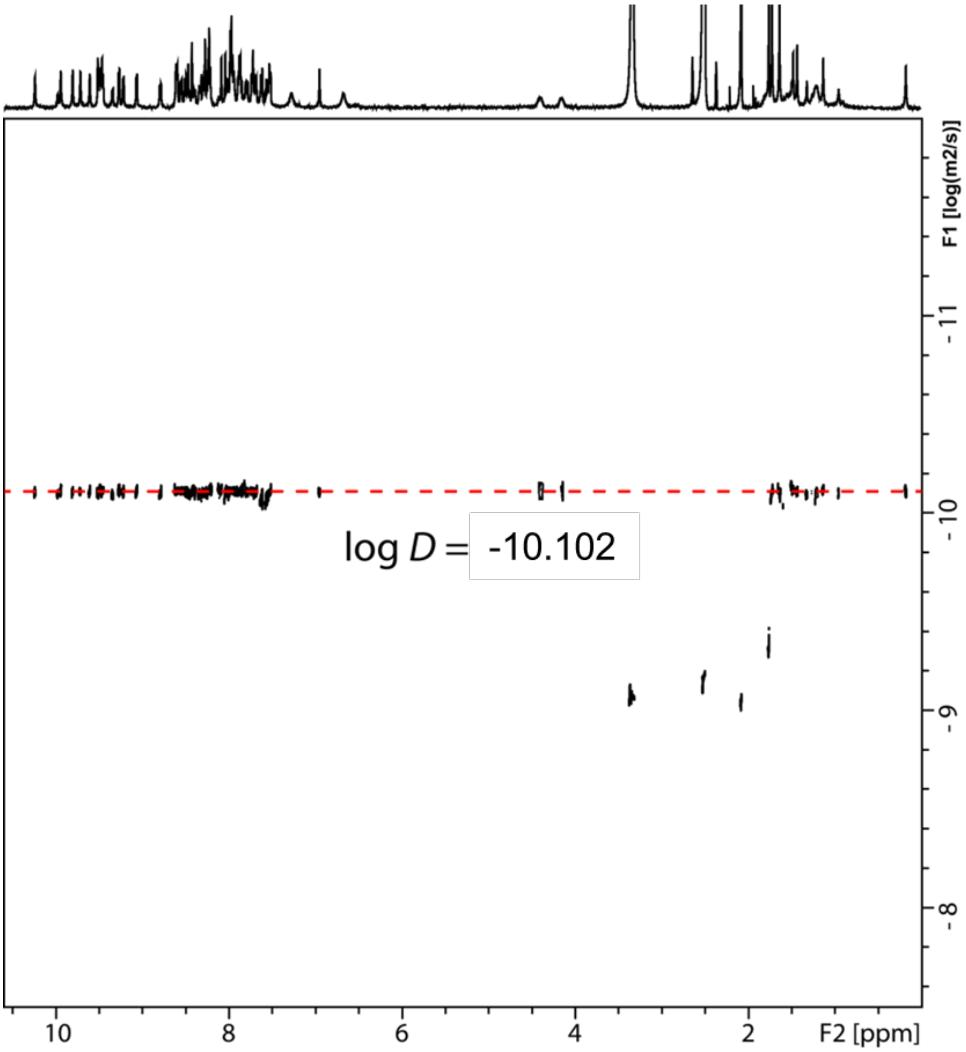

Figure S28. ^{1}H DOSY spectrum (500 MHz, 298K, DMSO-d_6) of heteroleptic pseudo-tetrahedron **T1**. Diffusion coefficient D = 7.908 x 10^{-11} m^2s^{-1}, log *D* = −10.102, r = 13.9 Å.

ESI-HRMS: *m/z*:

calc. for **$[Pd_3(L^{A1})(L^B)_4]^{6+}$** ($C_{158}H_{120}N_{14}Pd_3$): 422.2828, found: 422.2823;

calc. for **$[Pd_3(L^{A1})(L^B)_4+BF_4]^{5+}$** ($C_{158}H_{120}N_{14}BF_4Pd_3$): 524.1404, found: 524.1395;

calc. for **$[Pd_3(L^{A1})(L^B)_4+2BF_4]^{4+}$** ($C_{158}H_{120}N_{14}B_2F_8Pd_3$): 676.6761, found: 676.6751;

calc. for **$[Pd_3(L^{A1})(L^B)_4+3BF_4]^{3+}$** ($C_{158}H_{120}N_{14}$ $B_3F_{12}Pd_3$): 931.2364, found: 931.2344;

calc. for **$[Pd_3(L^{A1})(L^B)_4+4BF_4]^{2+}$** ($C_{158}H_{120}N_{14}B_4F_{16}Pd_3$): 1440.3567, found: 1440.3520.

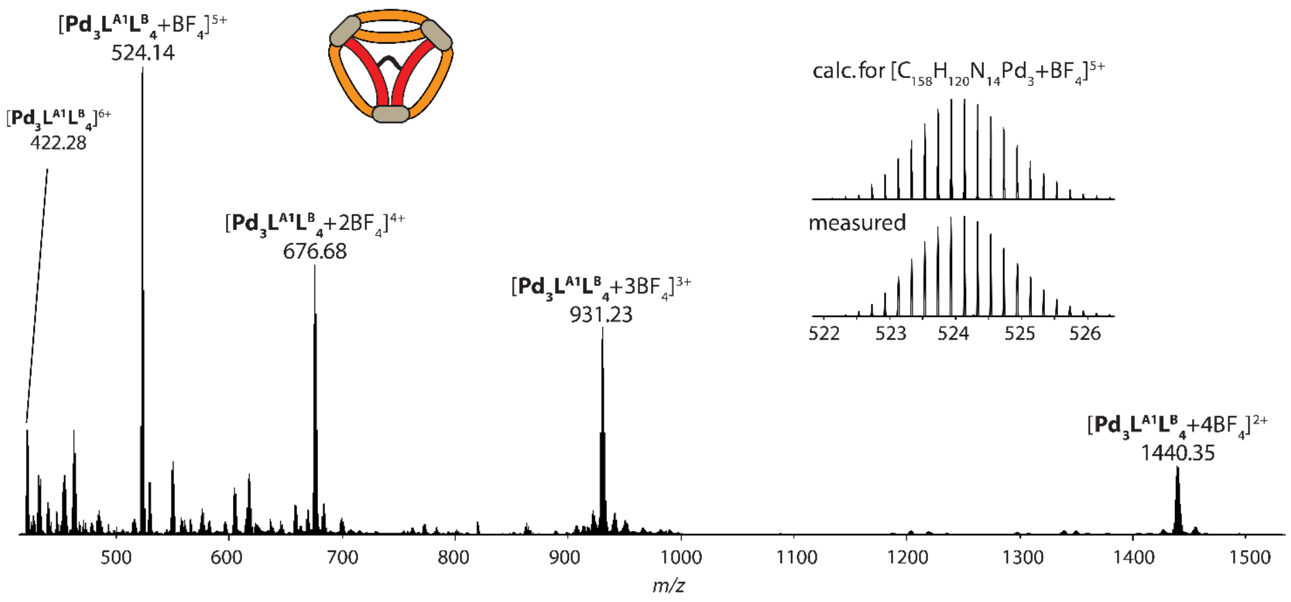

Figure S29. HR-ESI mass spectrum of heteroleptic pseudo-tetrahedron **T1**.

*1.3.6. Self-assembly of heteroleptic cage dimer **$[Pd_4(L^{A2})_2(L^B)_4]^{8+}$** (**D2**) in DMSO-$d_6$*

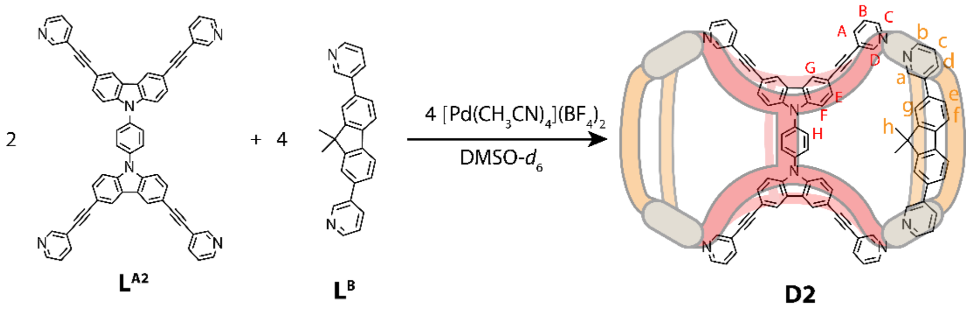

A suspension of $\mathbf{L^{A2}}$ (0.57 mg, 0.7 µmol) in 230 µL DMSO-d_6 and a solution of $\mathbf{L^B}$ (1.4 µmol, 7 mM) in 200 µL DMSO-d_6 was added to an NMR tube and carefully heated to obtain a clear solution. Then, a stock solution of $[Pd(CH_3CN)_4](BF_4)_2$ (70 µL, 20 mM/DMSO-d_6, 1.4 µmol) was added. The mixture was heated at 80 °C for 8 h to give a 0.7 mM solution of cage dimer **D2**.

^{1}H NMR (500 MHz, 298K, DMSO-d_6) δ 10.09 (s, 8H), 9.81 (s, 8H), 9.38 (d, *J* = 5.7 Hz, 8H), 9.26 (d, *J* = 5.9 Hz, 8H), 8.54 (s, 8H), 8.45 (d, *J* = 7.7 Hz, 8H), 8.33 (d, *J* = 7.8 Hz, 8H), 8.25 (d, *J* = 8.0 Hz, 8H), 8.15 (d, *J* = 7.4 Hz, 8H), 8.08 (s, 4H), 7.86 (m, 24H), 7.60 (d, *J* = 8.4 Hz, 8H), 7.53 (b, 12H), 1.40 (s, 12H), 0.82 (s, 12H).

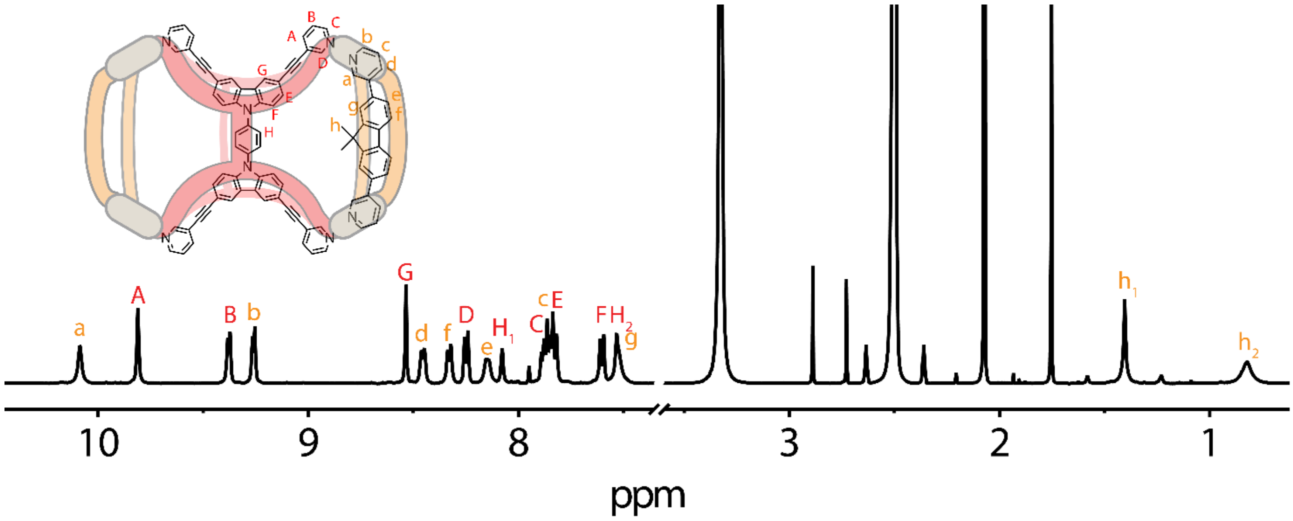

Figure S30. ^{1}H NMR spectrum (500 MHz, 298K, DMSO-d_6) of heteroleptic cage dimer **D2**.

^{13}C NMR (151 MHz, 298K, DMSO-d_6) δ 171.47, 162.33, 154.67, 153.53, 149.98, 149.64, 148.54, 141.62, 141.36, 139.45, 138.72, 135.18, 134.21, 131.12, 129.05, 128.31, 127.49, 124.34, 122.72, 122.60, 122.18, 120.99, 118.11, 112.41, 111.09, 95.77, 83.38, 47.30, 35.80, 30.79, 28.30, 24.25, 22.52.

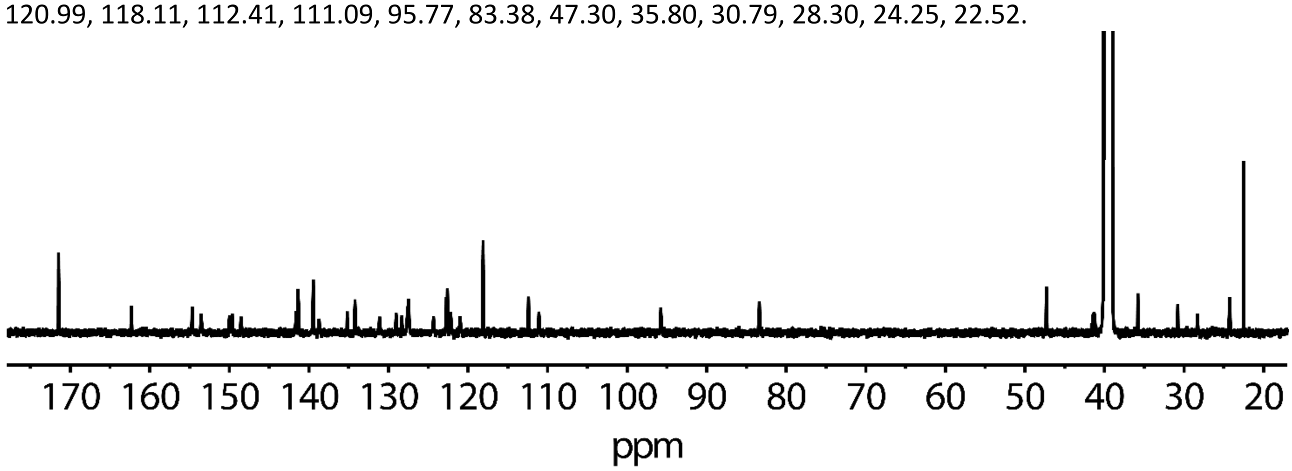

Figure S31. ^{13}C NMR spectrum (151 MHz, 298K, DMSO-d_6) of heteroleptic cage dimer **D2**.

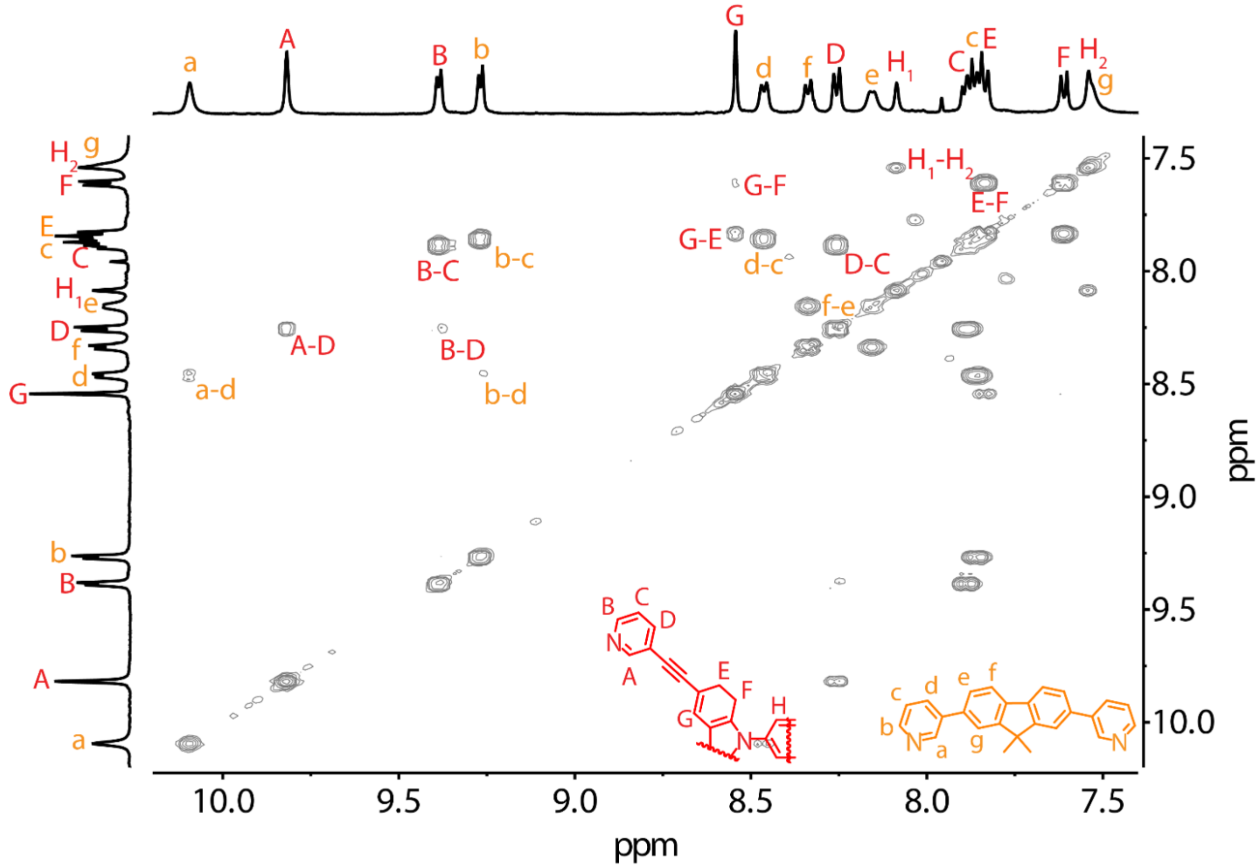

Figure S32. Partial ^{1}H–^{1}H COSY spectrum (500 MHz, 298K, DMSO-d_6) of heteroleptic cage dimer **D2**.

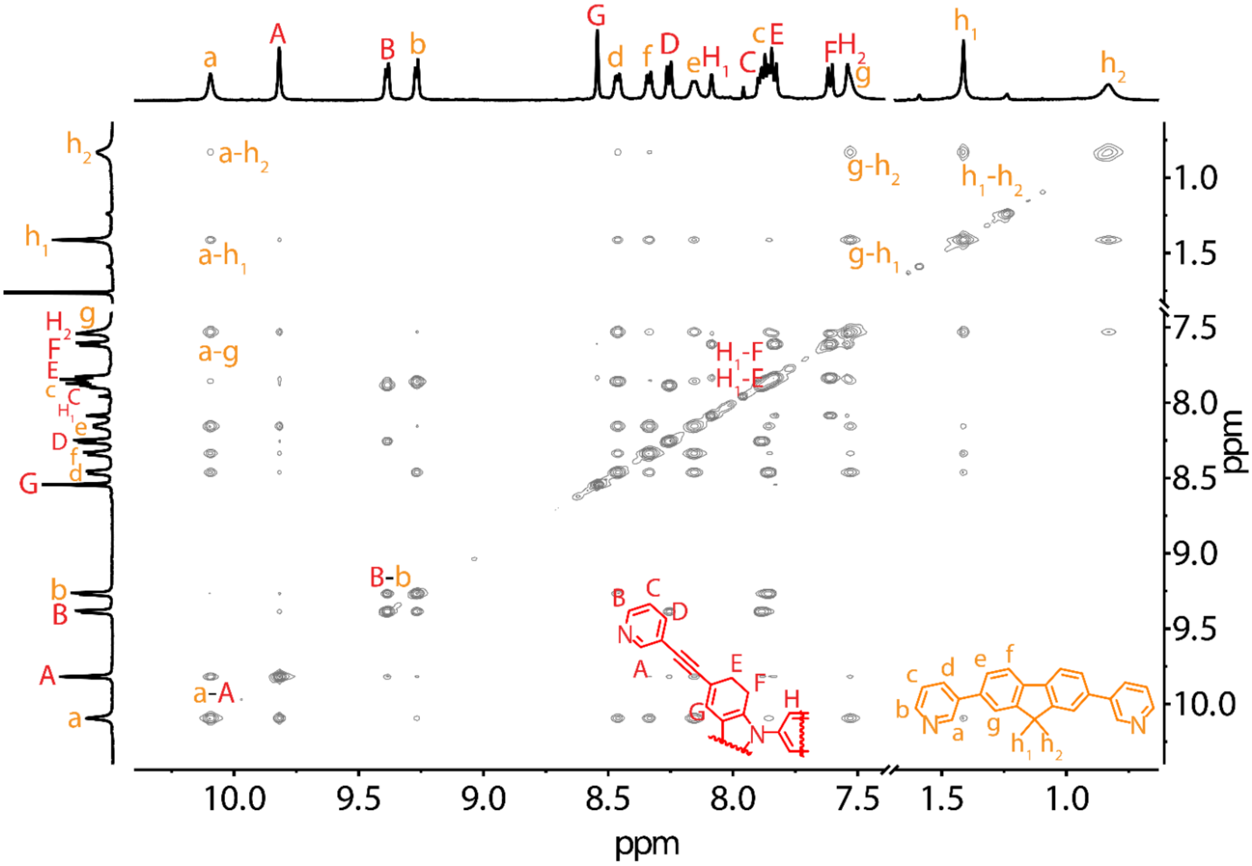

Figure S33. Partial ^{1}H–^{1}H NOESY spectrum (500 MHz, 298K, DMSO-d_6) of heteroleptic cage dimer **D2**.

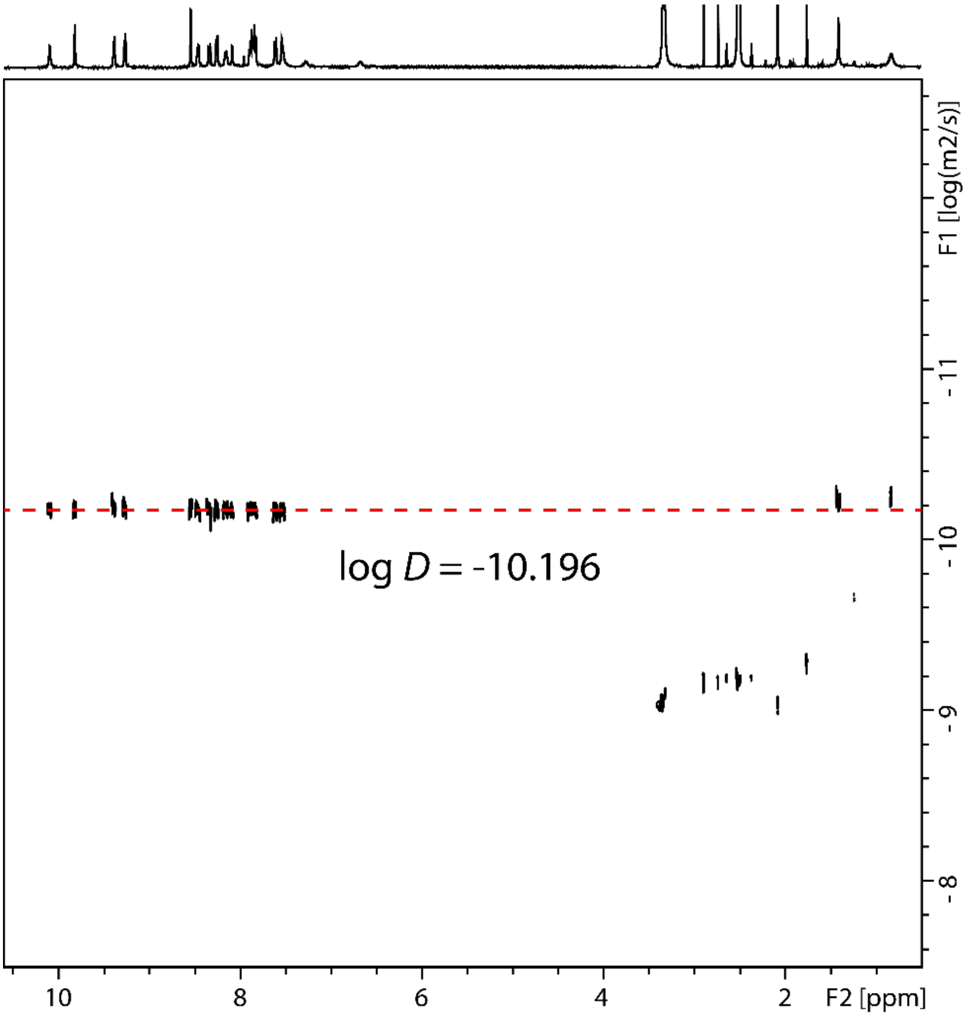

Figure S34. ^{1}H DOSY spectrum (500 MHz, 298K, DMSO-d_6) of heteroleptic cage dimer **D2**. Diffusion coefficient D = 6.361 x 10^{-11} m^2s^{-1}, log D = −10.196, r = 17.2 Å.

ESI-HRMS: *m/z*:

calc. for **$[Pd_4(L^{A2})_2(L^B)_4]^{8+}$**($C_{216}H_{144}N_{20}Pd_4$): 430.6010, found: 430.6004;

calc. for **$[Pd_4(L^{A2})_2(L^B)_4+BF_4]^{7+}$**($C_{216}H_{144}N_{20}BF_4Pd_4$): 504.5446, found: 504.5441;

calc. for **$[Pd_4(L^{A2})_2(L^B)_4+2BF_4]^{6+}$**($C_{216}H_{144}N_{20}B_2F_8Pd_4$): 603.1361, found: 603.1355;

calc. for **$[Pd_4(L^{A2})_2(L^B)_4+3BF_4]^{5+}$**($C_{216}H_{144}N_{20}$ $B_3F_{12}Pd_4$): 740.9641, found: 740.9631;

calc. for **$[Pd_4(L^{A2})_2(L^B)_4+4BF_4]^{4+}$**($C_{216}H_{144}N_{20}$ $B_4F_{16}Pd_4$): 947.9562, found: 947.9548;

calc. for **$[Pd_4(L^{A2})_2(L^B)_4+5BF_4]^{3+}$**($C_{216}H_{144}N_{20}$ $B_5F_{20}Pd_4$): 1292.9403, found: 1292.9430.

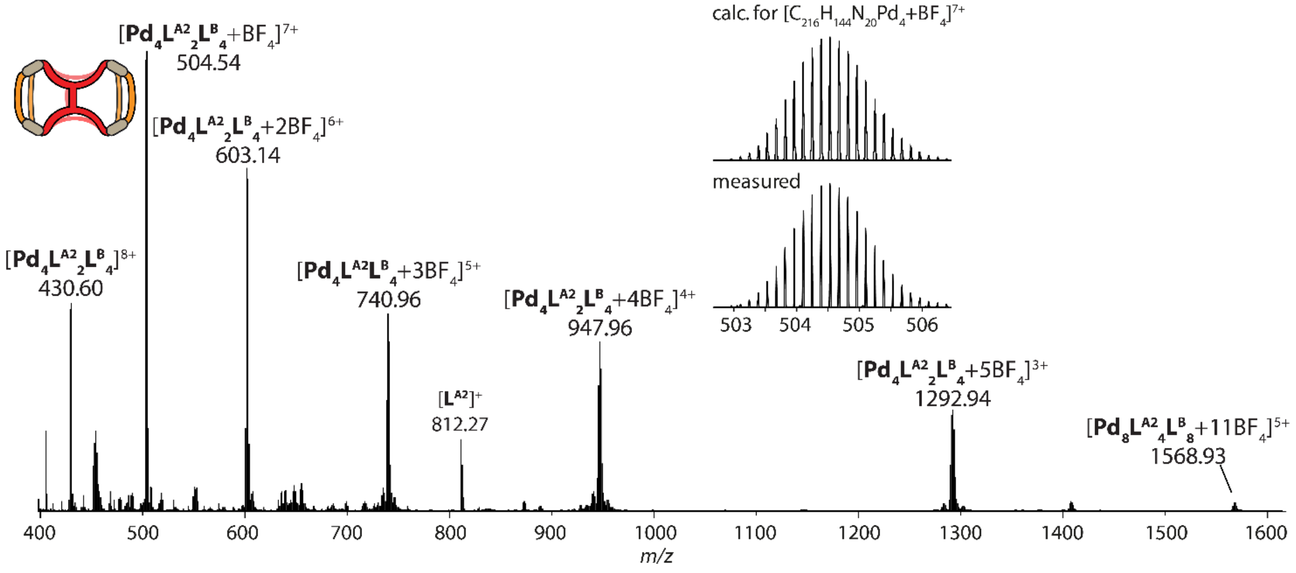

Figure S35. HR-ESI mass spectrum of heteroleptic cage dimer **D2**.

1.3.7. Self-assembly of heteroleptic cage dimer $[Pd_4(L^{A2})_2(L^B)_4]^{8+}$ *(**D2**) in DMF-*d_7

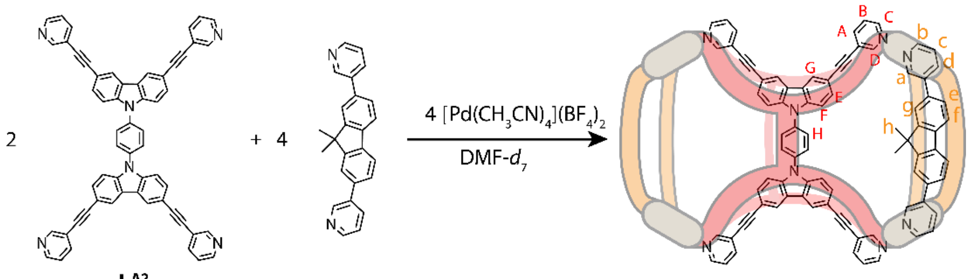

L^{A2} L^B **D2**

A suspension of $\mathbf{L^{A2}}$ (0.57 mg, 0.7 µmol) in 230 µL DMF-d_7 and a solution of $\mathbf{L^B}$ (1.4 µmol, 7 mM) in 200 µL DMF-d_7 was added to an NMR tube and carefully heated to obtain a clear solution. Then, a stock solution of $[Pd(CH_3CN)_4](BF_4)_2$ (70 µL, 20 mM/DMF-d_7, 1.4 µmol) was added. The mixture was heated at 80 °C for 8 h to give a 0.7 mM solution of cage **D2**.

^{1}H NMR (500 MHz, 298K, DMF-d_7) δ 10.40 (s, 8H), 10.07 (d, J = 2.0 Hz, 8H), 9.64 (dd, J = 5.9, 1.5 Hz, 8H), 9.58 – 9.51 (m, 8H), 8.63 (s, 8H), 8.59 (d, J = 8.0 Hz, 8H), 8.50 (d, J = 7.7 Hz, 8H), 8.30 (dt, J = 8.1, 1.6 Hz, 8H), 8.21 (d, J = 7.7 Hz, 8H), 8.08 (s, 4H), 8.03 (s, 4H), 7.96 (m, 16H), 7.89 – 7.80 (m, 16H), 7.68 (s, 8H), 1.55 (s, 12H), 0.93 (s, 12H).

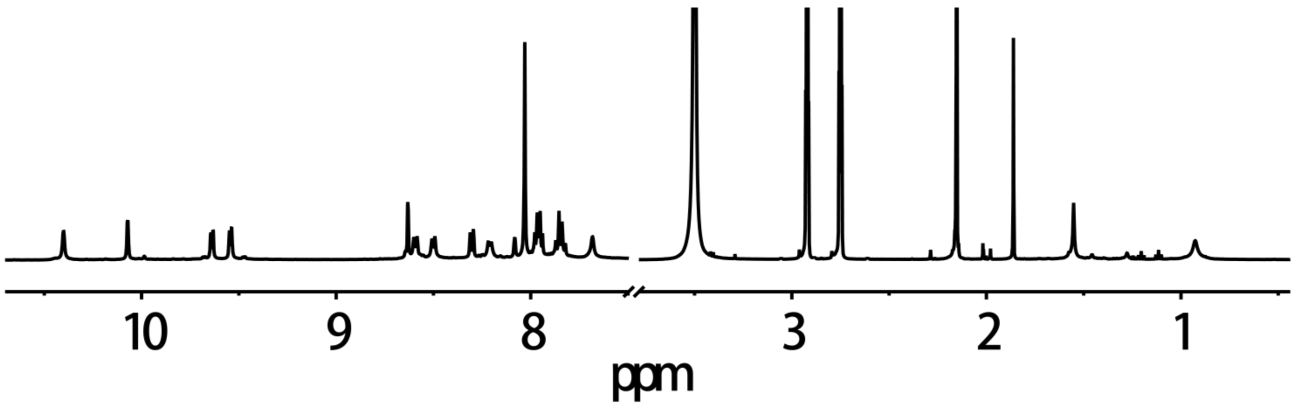

Figure S36. ^{1}H NMR spectrum (500 MHz, 298K, DMF-d_7) of heteroleptic cage dimer **D2**.

^{13}C NMR (151 MHz, 298K, DMF-d_7) δ 171.86, 155.53, 154.36, 150.54, 150.30, 149.25, 141.73, 141.63, 140.73, 140.34, 139.13, 135.70, 134.87, 131.84, 129.59, 128.76, 127.75, 122.98, 122.80, 121.69, 117.90, 113.49, 111.55, 96.56, 83.13, 47.89, 28.02, 24.83, 22.22.

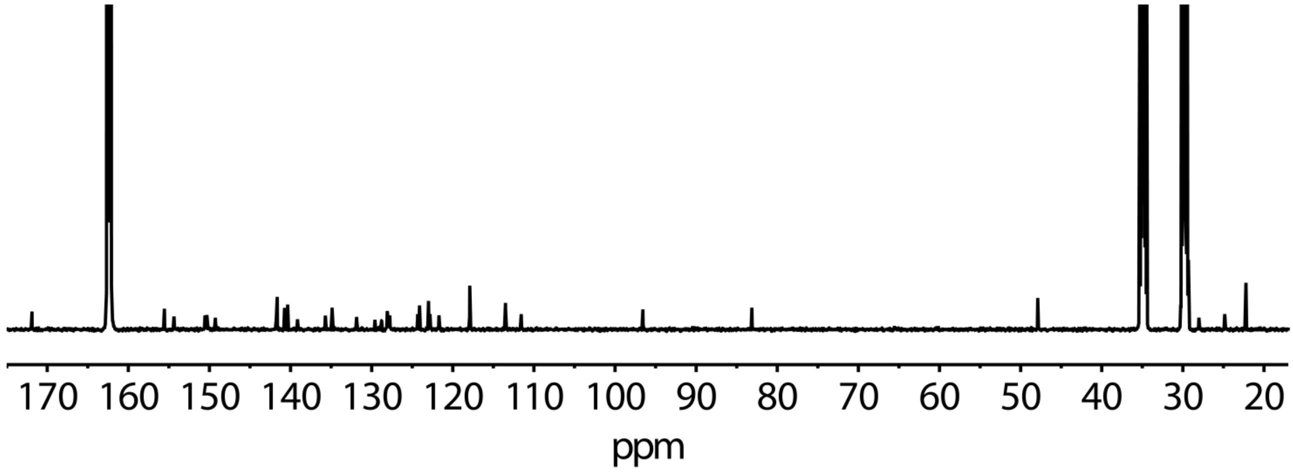

Figure S37. ^{13}C NMR spectrum (151 MHz, 298K, DMF-d_7) of heteroleptic cage dimer **D2**.

2. Cage-to-cage transformation

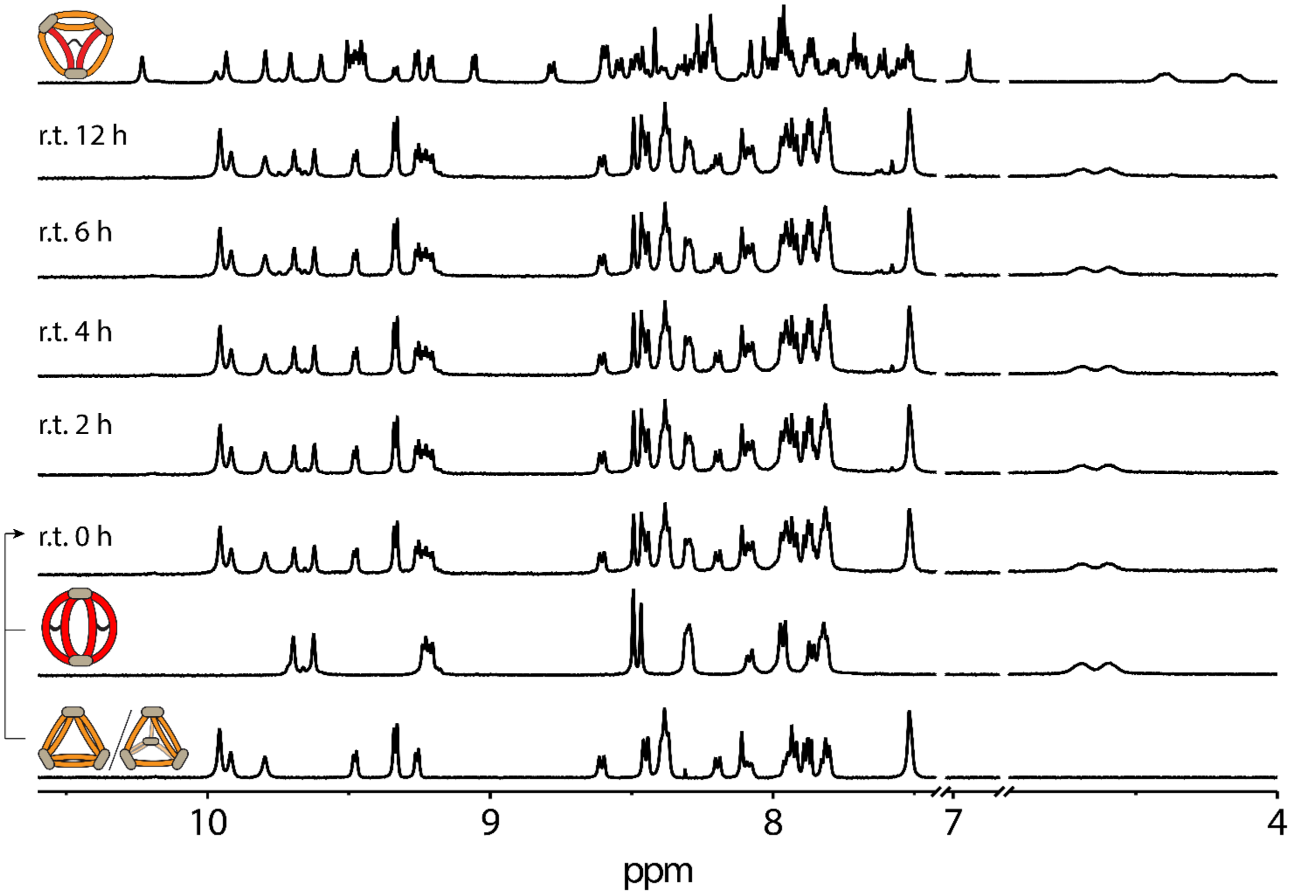

Figure S38. ^{1}H NMR spectra (500 MHz, 298K, DMSO-d_6) of the mixture of homoleptic bridged cage **C1** and a 1:1 mixture of homoleptic ring **R** and tetrahedron **T** at room temperature over the course of 12h. As can be seen, homoleptic species co-exist and no conversion to heteroleptic pseudo-tetrahedron **T1** is achieved at this temperature.

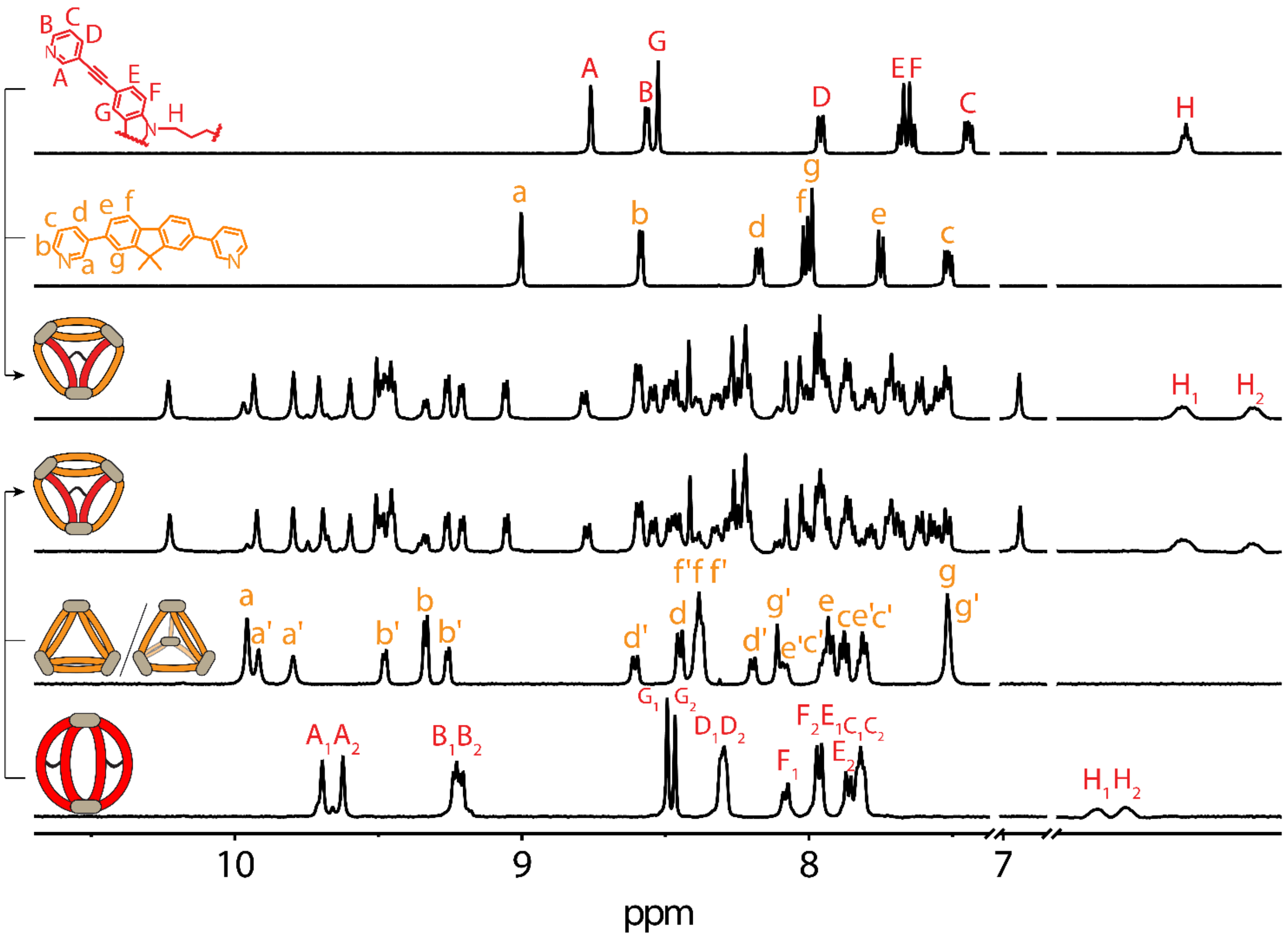

Figure S39. ^{1}H NMR spectra (500 MHz, 298K, DMSO-d_6) of pseudo-tetrahedron **T1** formation from free ligands and Pd(II) cations (top) compared to cage-to-cage transformation from homoleptic bridged cage **C1** and a 1:1 mixture of homoleptic ring **R** and tetrahedron **T** to the same heteroleptic pseudo-tetrahedron **T1** after heating at 80 °C for 2 h.

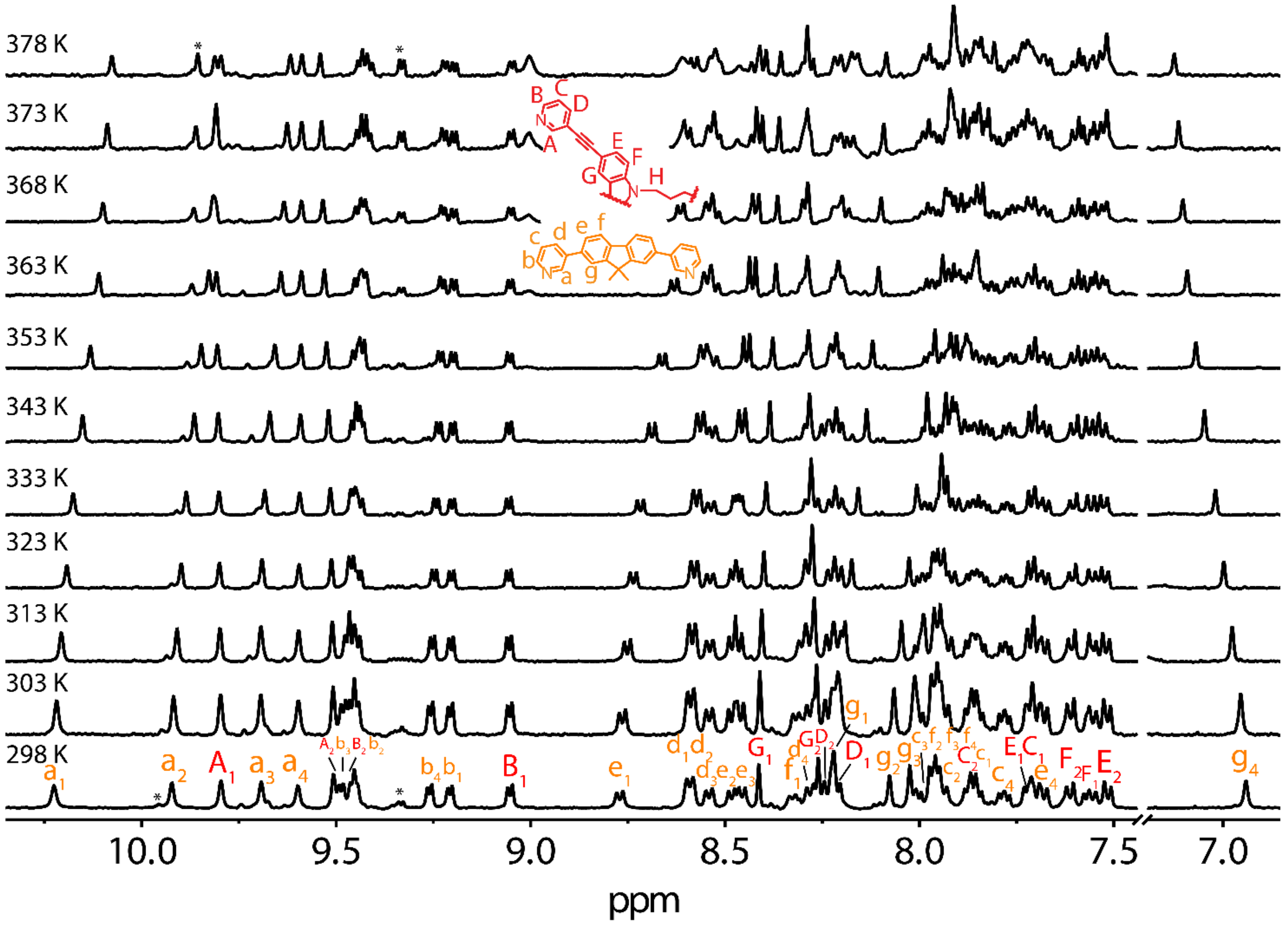

Figure S40.^{1}H NMR spectra (500 MHz, DMSO-d_6) to compare the behaviour of heteroleptic pseudo-tetrahedron **T1** at different temperatures. (* = homoleptic ring **R**)

This experiment shows that the heteroleptic pseudo-tetrahedron **T1** is stable in solution up to 378 K. Moreover, the split proton signals do not coalesce even at elevated temperatures, indicating that the signals splitting is caused by an inherently low symmetry of the topology (not by conformational locking effects), further supporting the assignment to species **T1** instead of **R1**.

3. Host-Guest study

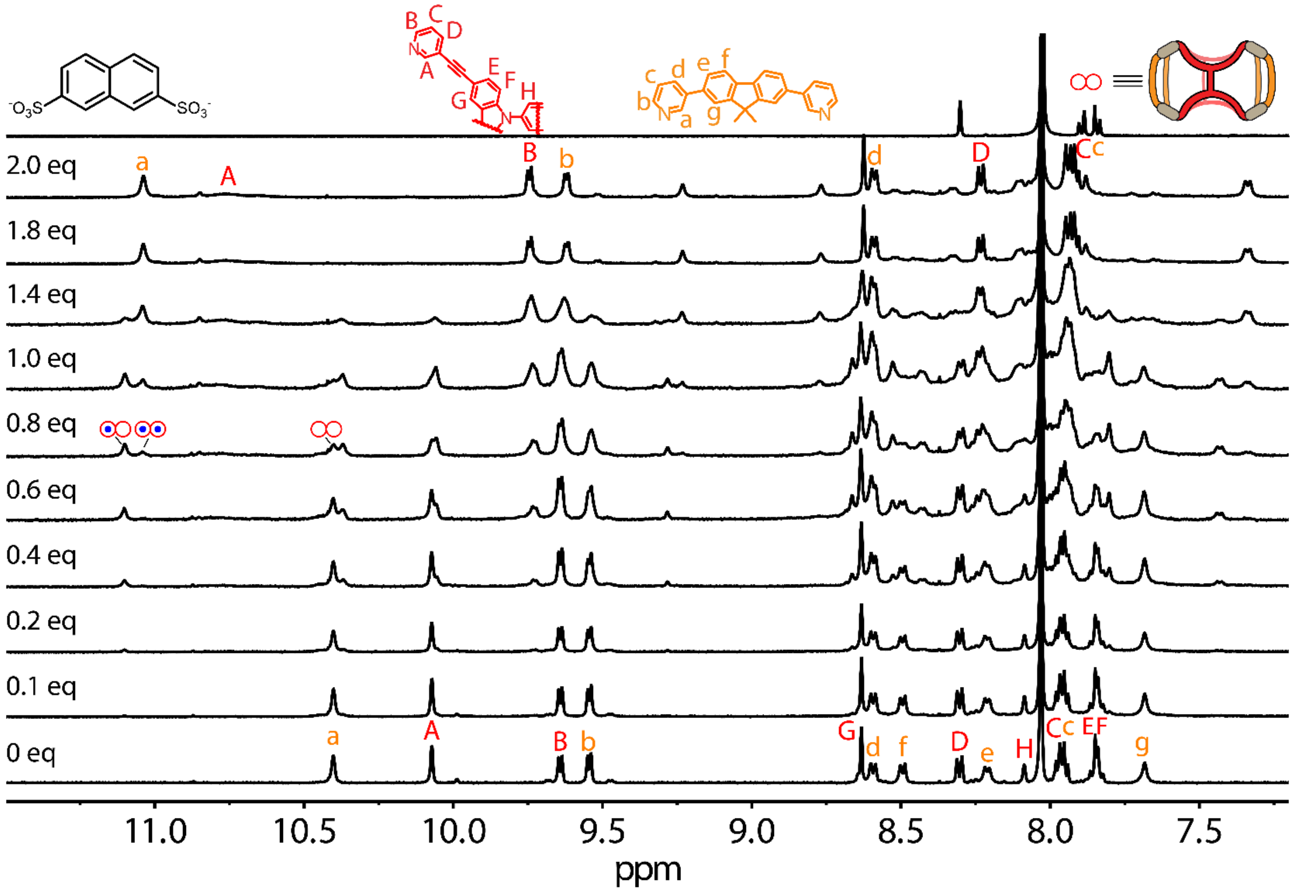

Figure S41. ^{1}H NMR titration (500 MHz, 298K, DMF-d_7) of heteroleptic cage dimer **D2** with G^1 in DMF-d_7 (The red double circle represents signal from empty cage dimer **D2**, while the one with one blue and two blue dots inside represent G^1**@D2** and $2G^1$**@D2**, respectively).

As the host-guest interactions between cage dimer **D1** and G^1 were observed to follow slow exchange kinetics, the concentrations of G^1**@D2**, $2G^1$**@D2** and **D2** could all be estimated from the ^{1}H NMR spectroscopic results. The integrals of protons **a** and **b** of ligand L^B were used to approximate K_1 and K_2 by using the following equations:

$$H + G \rightleftarrows HG \quad and \quad HG + G \rightleftarrows HG_2$$

$$K_1 = \frac{[HG]}{[H][G]} \quad and \quad K_2 = \frac{[HG_2]}{[HG][G]}$$

where H and G represent host cage dimer **D2** and guest G^1, respectively. Concentrations obtained from three distinct ^{1}H NMR spectra (0.6, 0.8 - 1.0 eq) are tabulated in **Table S1**. Further, from this data, the cooperativity parameter $\alpha = 4K_2/K_1$ was calculated.[7]

Table S1. Data extracted from the ^{1}H NMR spectra of G^1 added to **D2** to quantify the cooperativity of guest binding.

Spectrum	0.6 eq. / mM	0.8 eq. / mM	1.0 eq. / mM	Average
[G]	0.210	0.202	0.270	
[H]	0.490	0.430	0.378	
[HG]	0.210	0.182	0.216	
[HG_2]	0	0.088	0.107	
K_1	2.04×10^3 M^{-1}	2.09×10^3 M^{-1}	2.12×10^3 M^{-1}	$(2.08 \pm 0.04) \times 10^3$ M^{-1}
K_2	-	2.39×10^3 M^{-1}	1.83×10^3 M^{-1}	$(2.11 \pm 0.28) \times 10^3$ M^{-1}
α	-	4.57	3.45	4.01

4. Ion Mobility Mass Spectrometry

Ion mobility measurements were performed on a Bruker timsTOF instrument combining a trapped ion mobility (TIMS) with a time-of-flight (TOF) mass spectrometer in one instrument. In contrast to the conventional drift tube method to determine mobility data, where ions are carried by an electric field through a stationary drift gas, the TIMS method is based on an electric field ramp to hold ions in place against a carrier gas pushing them in the direction of the analyzer. Consequently, larger sized ions that experience more carrier gas impacts leave the TIMS units first and smaller ions elute later. This method offers a much higher mobility resolution despite a smaller device size.

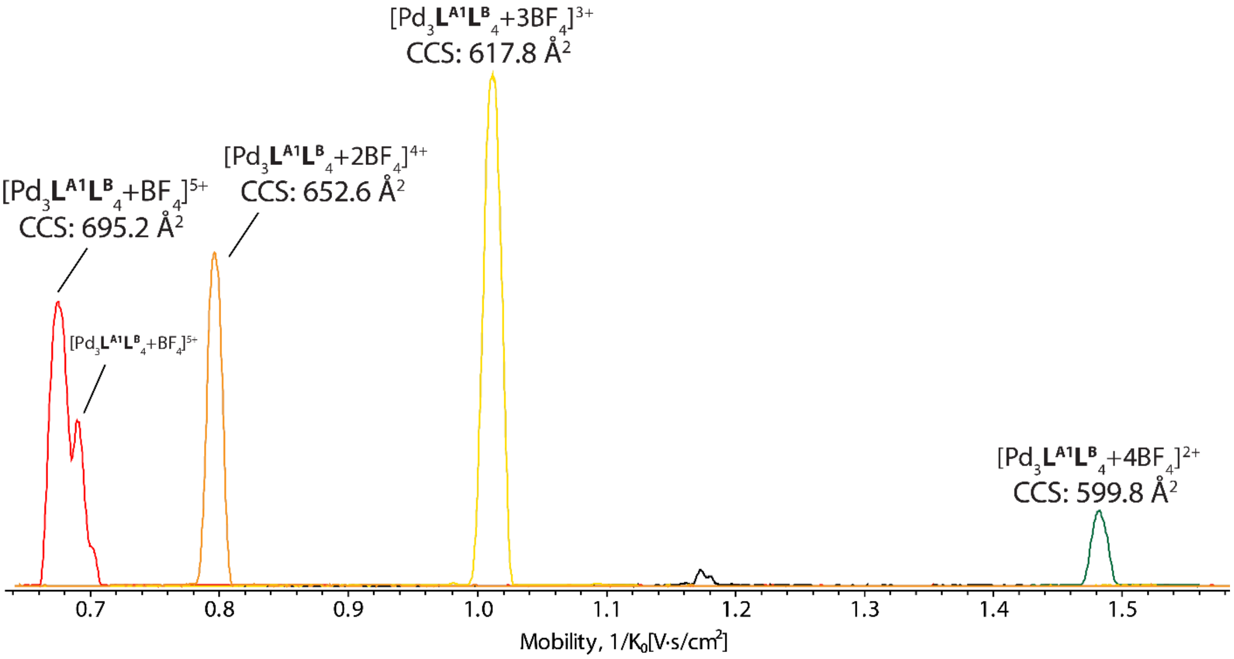

Figure S42. Mobilograms obtained by trapped ion mobility ESI-TOF mass spectrometry for heteroleptic pseudo-tetrahedron **T1**: **$[Pd_3(L^{A1})(L^B)_4+BF_4]^{5+}$** (CCS: 695.2 Å^2 and 711.0 Å^2 at *m/z* 523.94), **$[Pd_3(L^{A1})(L^B)_4+2BF_4]^{4+}$** (CCS: 652.6 Å^2 at *m/z* 676.68), **$[Pd_3(L^{A1})(L^B)_4+3BF_4]^{3+}$** (CCS: 617.8 Å^2 at *m/z* 931.23), **$[Pd_3(L^{A1})(L^B)_4+4BF_4]^{2+}$** (CCS: 599.8 Å^2 at *m/z* 1440.85).

Table S2. Comparison of experimental collisional cross section (eCCS) values of heteroleptic pseudo-tetrahedron with results derived from Collidoscope software[8] (tCCS) based on the CREST[9] (GFN2-xTB) generated models with corresponding number of encapsulated BF_4^- counter anions.

Species	eCCS [Å^2]	tCCS (T1) [Å^2]	$\Delta_{\%}$ (T1)	tCCS (R1) [Å^2]	$\Delta_{\%}$ (R1)
$[Pd_3\mathbf{L}^{A1}\mathbf{L}^B{}_4+BF_4]^{5+}$	695.2	713.8	2.7%	732.5	5.4%
$[Pd_3\mathbf{L}^{A1}\mathbf{L}^B{}_4+2BF_4]^{4+}$	652.6	671.9	3.0%	691.7	6.0%
$[Pd_3\mathbf{L}^{A1}\mathbf{L}^B{}_4+3BF_4]^{3+}$	617.8	644.9	4.4%	658.6	6.6%
$[Pd_3\mathbf{L}^{A1}\mathbf{L}^B{}_4+4BF_4]^{2+}$	599.8	643.3	7.3%	652.5	8.8%

Experimental and theoretical data show in accordance that the CCS decreases with increasing number of encapsulated BF_4^- counter anions, leading to a stepwise decrease of overall charge. This common phenomenon can be explained by weaker ion–induced dipole and ion–quadrupole interactions with the carrier gas molecules (N_2),[10] and this trend is reproducible by the theoretical calculations. The same observation was also made for the heteroleptic dimer (Table S3).

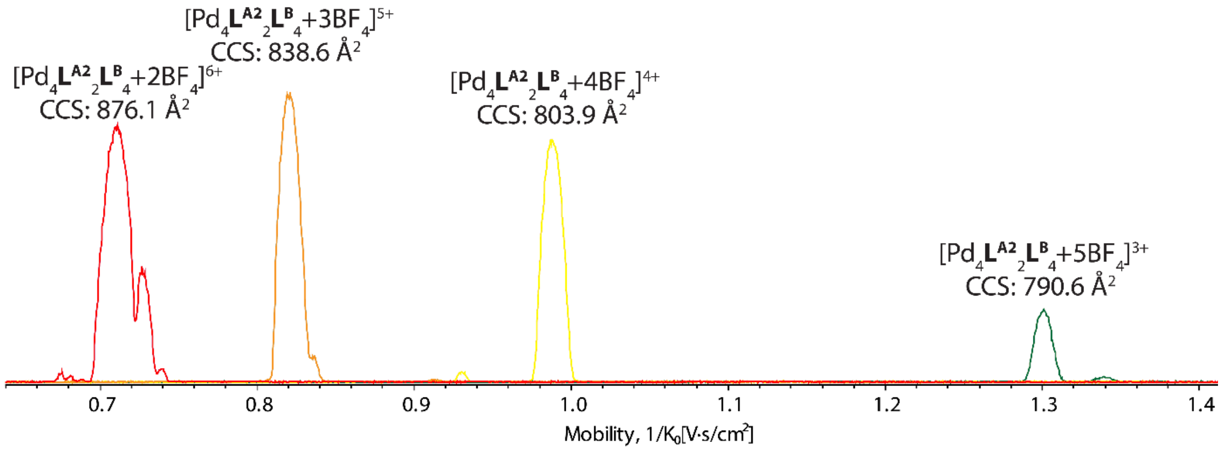

Figure S43. Mobilograms obtained by trapped ion mobility ESI-TOF mass spectrometry for heteroleptic cage dimer **D2**: **[Pd$_4$(L^{A2})$_2$(L^B)$_4$+2BF$_4$]$^{6+}$** (CCS: 876.1 Å^2 and 895.3 Å^2 at *m/z* 603.14), **[Pd$_4$(L^{A2})$_2$(L^B)$_4$+3BF$_4$]$^{5+}$** (CCS: 838.6 Å^2 at *m/z* 740.96), **[Pd$_4$(L^{A2})$_2$(L^B)$_4$+4BF$_4$]$^{4+}$** (CCS: 803.9 Å^2 at *m/z* 947.96), **[Pd$_4$(L^{A2})$_2$(L^B)$_4$+5BF$_4$]$^{3+}$** (CCS: 790.6 Å^2 at *m/z* 1292.94).

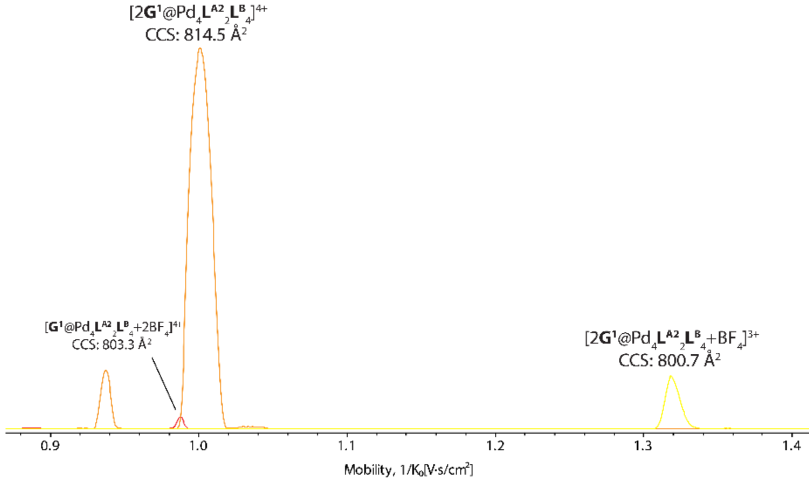

Figure S44. Mobilograms obtained by trapped ion mobility ESI-TOF mass spectrometry for heteroleptic cage dimer **D2** with **G^1**: **[G^1@Pd$_4$(L^{A2})$_2$(L^B)$_4$+2BF$_4$]$^{4+}$** (CCS: 803.3 Å^2 at *m/z* 976.19), **[2G^1@Pd$_4$(L^{A2})$_2$(L^B)$_4$]$^{4+}$** (CCS: 814.5 Å^2 at *m/z* 1004.43), **[2G^1@Pd$_4$(L^{A2})$_2$(L^B)$_4$+BF$_4$]$^{3+}$** (CCS: 800.7 Å^2 at *m/z* 1367.91).

Table S3. Comparison of experimental collisional cross section (eCCS) values of heteroleptic dimer **D2** and 2G^1@**D2** with results derived from Collidoscope software[8] (tCCS) based models, which were optimized using B97-3c (ORCA, ver. 4.2.1)[11], with corresponding encapsulated BF$_4^-$ counter anions.

**Species**	**eCCS [Å^2]**	**tCCS [Å^2]**	**tCCS Δ$_{\%}$**
[Pd$_4$**L**^{A2}$_2$**L**^{B}$_4$+2BF$_4$]$^{6+}$	876.1	872.5	-0.4%
[Pd$_4$**L**^{A2}$_2$**L**^{B}$_4$+3BF$_4$]$^{5+}$	838.6	844.7	0.7%
[Pd$_4$**L**^{A2}$_2$**L**^{B}$_4$+4BF$_4$]$^{4+}$	803.9	821.7	2.2%
[Pd$_4$**L**^{A2}$_2$**L**^{B}$_4$+5BF$_4$]$^{3+}$	790.6	825.1	4.4%
[G^1@Pd$_4$**L**^{A2}$_2$**L**^{B}$_4$+2BF$_4$]$^{4+}$	803.3	850.7	6.3%
[2G^1@Pd$_4$**L**^{A2}$_2$**L**^{B}$_4$]$^{4+}$	814.5	830.0	1.9%
[2G^1@Pd$_4$**L**^{A2}$_2$**L**^{B}$_4$+BF$_4$]$^{3+}$	800.7	834.2	4.2%

The data reveals that host-guest complex [2G^1@Pd$_4$**L**^{A2}$_2$**L**^{B}$_4$]$^{4+}$ features a slightly higher CCS value than "empty" host [Pd$_4$**L**^{A2}$_2$**L**^{B}$_4$+4BF$_4$]$^{4+}$ (peak with four counter anions chosen to allow direct comparison), which in turn has a similar CCS value compared to the species with only one guest bound.

5. Computational studies

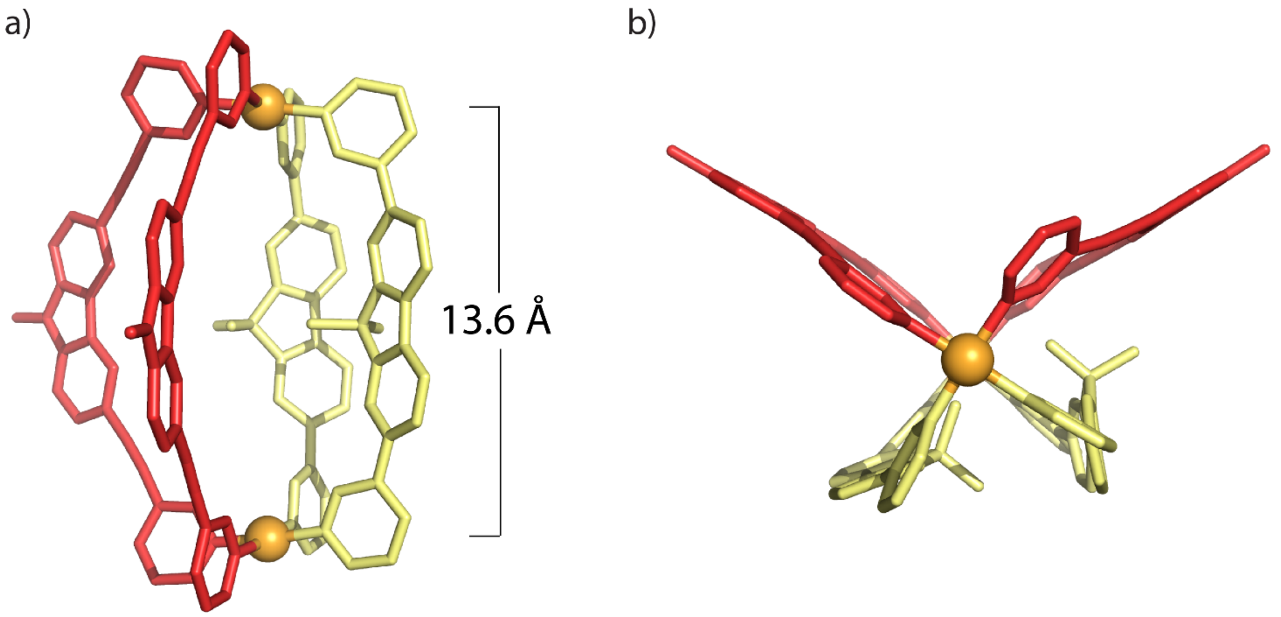

Figure S45. Model of heteroleptic cage **C** optimized with B3LYP/def2-SVP (ORCA 4.2.1)[11] in different views, a) side view, b) top view (a methyl group was used for ligand $\mathbf{L^A}$ instead of its hexyl group to reduce calculation time. Two BF_4^- counter anions inside the cavity are omitted for clarity).

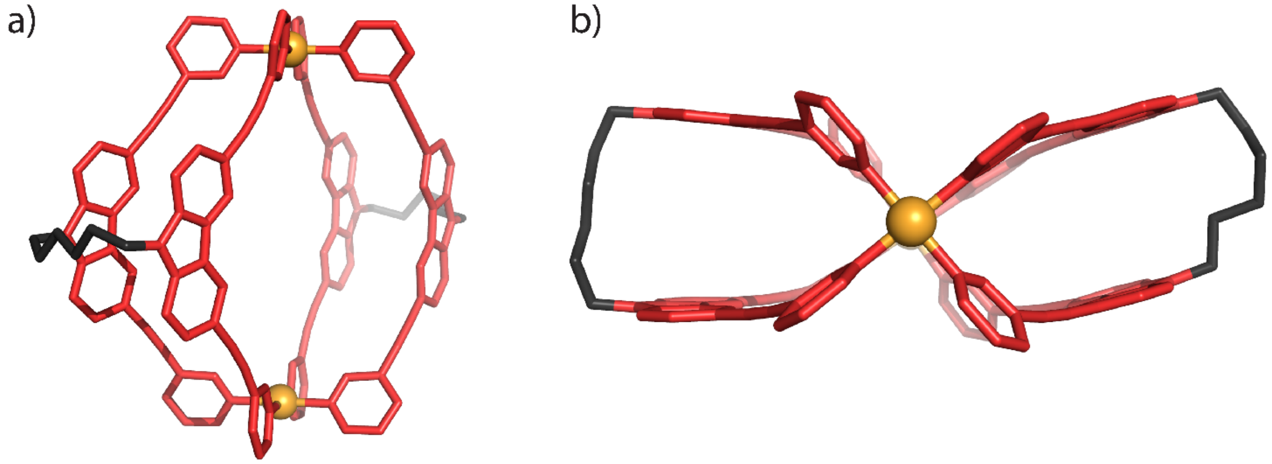

Figure S46. Model of bridged homoleptic cage C1, optimized with B3LYP/def2-SVP (ORCA 4.2.1)[11] in different views, a) side view, b) top view (two BF_4^- counter anions inside the cavity are omitted for clarity).

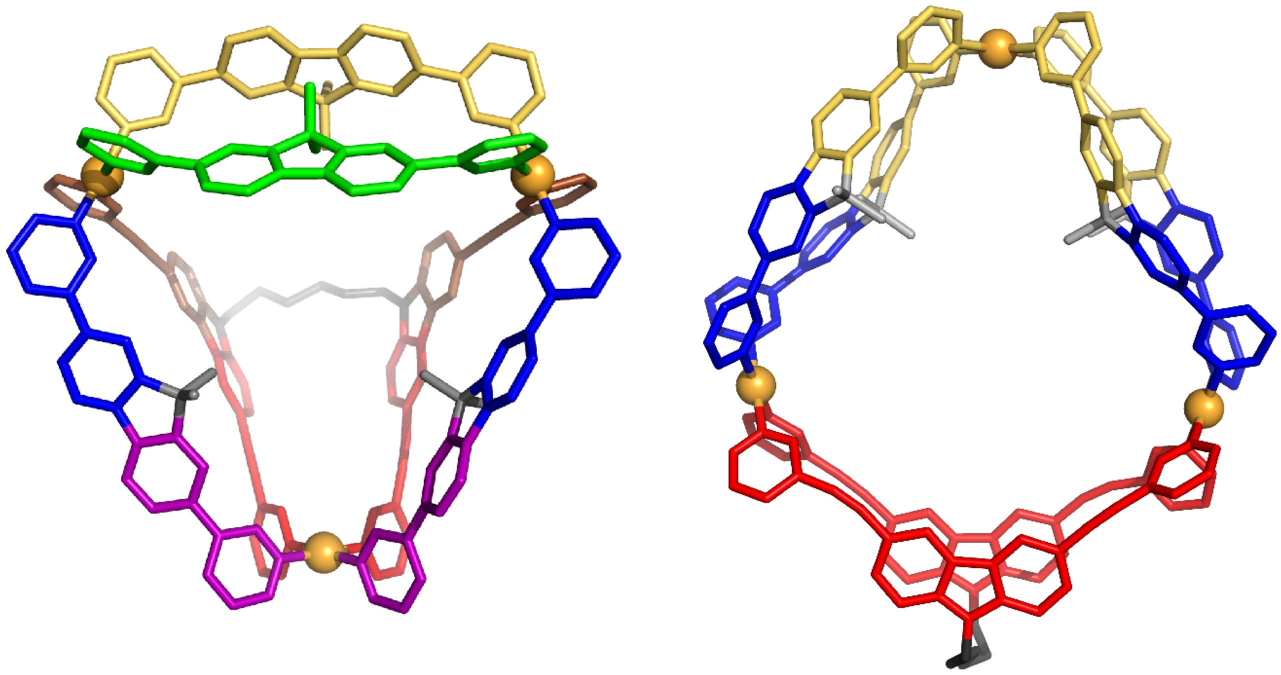

T1 **R1**

Figure S47. Models of two isomeric assemblies of sum formula $[Pd_3(\mathbf{L^{A1}})(\mathbf{L^{B}})_4]$. Left: heteroleptic pseudo-tetrahedron **T1** and right: heteroleptic three-ring **R1**, both showing different topologies (optimized with B97-3c (ORCA 4.2.1)[11]. A subsequent single point energy calculation shows that **T1** is energetically more favourable than **R1** with a difference of 24 kJ/mol. Colours indicate different chemical environments for substructures of ligands $\mathbf{L^{A1}}$ and $\mathbf{L^{B}}$.

6. X-ray Crystallography

Table S4. Crystallographic data of $[Pd_4(\mathbf{L^{A1}})_2(\mathbf{L^{B}})_4](BF_4)_8$ (**D1**), $[Pd_4(\mathbf{L^{A2}})_2(\mathbf{L^{B}})_4](BF_4)_8$ (**D2**), and $[2\mathbf{G^1}+Pd_6(\mathbf{L^{A2}})_3(\mathbf{L^{B}})_6](BF_4)_8$ (**P2**).

Compound	**D1**	**D2**	**P2**
Identification code	kw8i_sq	kw7j_sq	kw25i_sq
CCDC number	2015325	2015326	2015327
Empirical formula	$C_{252}H_{230}B_8F_{32}N_{30}O_4Pd_4$	$C_{240}H_{200}B_6F_{24}N_{28}O_8Pd_4$	$C_{352}H_{264}B_4F_{16}N_{36}O_{12}Pd_6S_2$
Formula weight	4862.73	4550.73	6239.74
Temperature (K)	100(2)	100(2)	100(2)
Crystal system	monoclinic	monoclinic	triclinic
Space group	$C2/c$	$C2/m$	$P\bar{1}$
a (Å)	37.7368(13)	33.2915(16)	24.9670(9)
b (Å)	21.2625(7)	47.548(2)	33.9028(9)
c (Å)	33.7991(11)	11.0729(4)	34.7518(11)
α (º)	90	90	107.529(2)
β (º)	112.9490(10)	95.136(2)	102.468(2)
γ (º)	90	90	105.612(2)
Volume (Å^3)	24973.2(15)	17457.2(13)	25563.7(15)
Z	4	2	2
Density (calc.) (Mg/m^3)	1.293	0.866	0.811
Absorption coefficient (mm^{-1})	2.984	2.085	2.120
F(000)	9984	4660	6392
Crystal size (mm^3)	0.400×0.100×0.100	0.150×0.050×0.050	0.100×0.050×0.050
Crystal colour	yellow	colourless	colourless
Crystal shape	block	needle	needle
Radiation	CuK_α (λ=1.54178 Å)	CuK_α (λ=1.54178 Å)	CuK_α (λ=1.54178 Å)
2θ range for data collection (º)	4.87 to 149.59 (0.80 Å)	3.25 to 108.46 (0.95 Å)	2.82 to 76.15 (1.25 Å)
Reflections collected	213510	77240	82967
Independent reflections [R(int)]	25527 [0.0609]	10798 [0.1163]	27072 [0.0994]
Data / restraints / parameters	25527/552/1624	10798/1449/776	27072/7592/3724
Goodness-of-fit on F^2	1.052	1.334	1.177
R_1 [I>2σ(I)]	0.0504	0.1360	0.0992
wR_2 (all data)	0.1443	0.3858	0.3434
Largest diff. peak/hole (eÅ^3)	1.65/-1.03	1.25/-0.51	1.03/-0.53

6.1. Crystal structure of D1

Yellow block-shaped crystals of $[Pd_4(\mathbf{L^{A1}})_2(\mathbf{L^{B}})_4](BF_4)_8$ (**D1**) were grown by slow vapor diffusion of Et_2O into a solution of the assembly product of **L**$^{\mathbf{A1}}$ and **L**$^{\mathbf{B}}$ in CD_3CN. Data was collected in-house on a Bruker D8 venture diffractometer equipped with an INCOATEC microfocus sealed tube (Iµs 3.0) using CuK_α radiation at 100 K. The data was integrated with APEX3 and the structure was solved by intrinsic phasing/direct methods using SHELXT[12] and refined with SHELXL[13] for full-matrix least-squares routines on F^2 and ShelXle[14] as a graphical user interface and the DSR[15] program plugin was employed for modeling.

6.1.1. Specific refinement details of ***D1****.*

Stereochemical restraints for the ligands (**L**$^{\mathbf{A1}}$ and **L**$^{\mathbf{B}}$) were generated by the GRADE program using the GRADE Web Server (http://grade.globalphasing.org) and applied in the refinement. A GRADE dictionary for SHELXL contains target values and standard deviations for 1,2-distances (DFIX) and 1,3-distances (DANG), as well as restraints for planar groups (FLAT). All displacements for non-hydrogen atoms were refined anisotropically. The refinement of ADP's for carbon, nitrogen and oxygen atoms was enabled by a combination of similarity restraints (SIMU) and rigid bond restraints (RIGU). The contribution of the electron density from disordered counterions and solvent molecules, which could not be modeled with discrete atomic positions were handled using the SQUEEZE routine in PLATON. The solvent mask file (.fab) computed by PLATON was included in the SHELXL refinement via the ABIN instruction leaving the measured intensities untouched.

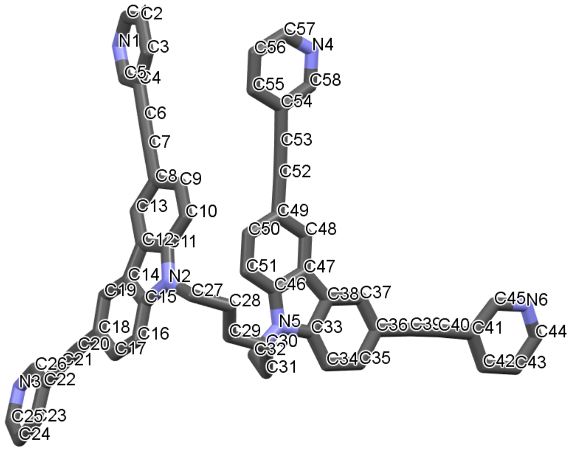

Figure S48. Atomic numbering scheme of residue CHC (ligand **L**$^{\mathbf{A1}}$).

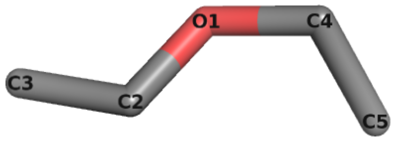

Figure S49. Atomic numbering scheme of residue ETO (diethyl ether solvent molecule).

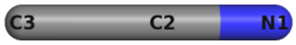

Figure S50. Atomic numbering scheme of residue ACN (CH_3CN solvent molecule).

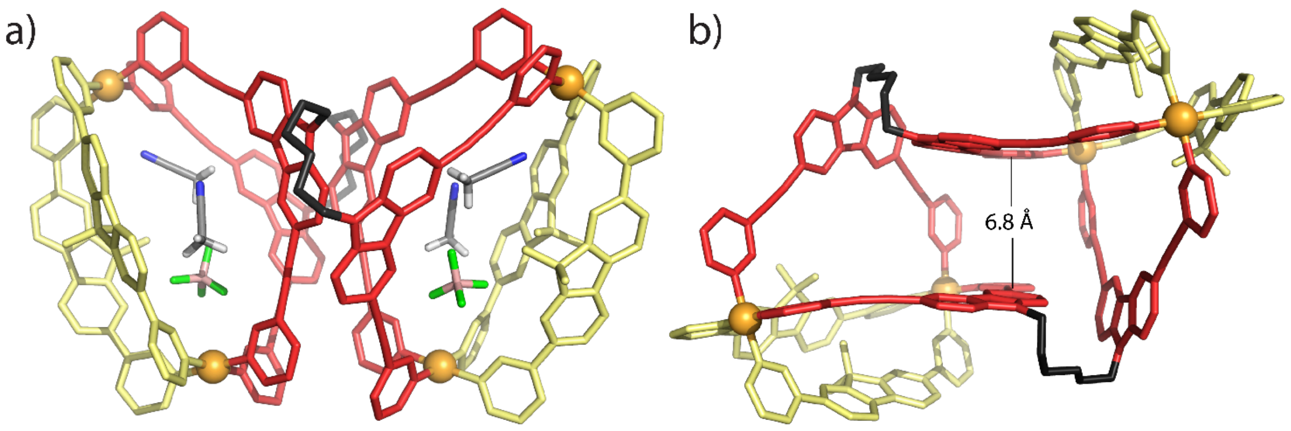

Figure S51. View of cage dimer **D1** crystal structures: a) showing encapsulated two CH_3CN molecules and one BF_4^- anion in each of the outer two cavities, b) showing the distance of coplanar aromatic ligand panels of the carbazole ligand of the wedge-shaped central cavity (distance is given in Ångström; hydrogens, BF_4^- anions and other solvent molecules are omitted for clarity).

Table S 5. Structural details of cage dimer **D1**.

Atoms	Distance [Å]	Esd [Å]
Pd2_1 Pd1_1	13.5967	0.0005
Pd2_1 Pd2_1**$1**	9.8691	0.0005
Pd1_1 Pd1_1**$1**	20.6544	0.0007
Pd1_1 Pd2_1**$1**	19.3550	0.0005
Symmetry code: **$1**=1-x, +y, 3/2-z		

6.2. Crystal structure of D2

Colorless needle-shaped crystals of $[Pd_4(\mathbf{L^{A2}})_2(\mathbf{L^{B}})_4](BF_4)_8$ (**D2**) were grown by slow vapor diffusion of isopropyl ether into a solution of **D2** in DMF. Data was collected in-house on a Bruker D8 venture diffractometer equipped with an INCOATEC microfocus sealed tube (Iµs 3.0) using CuK_α radiation at 100 K. The data was integrated with APEX3 and the structure was solved by intrinsic phasing/direct methods using SHELXT[12] and refined with SHELXL[13] for full-matrix least-squares routines on F^2 and ShelXle[14] as a graphical user interface and the DSR[15] program plugin was employed for modeling.

*6.2.1. Specific refinement details of **D2**.*

Stereochemical restraints for the ligands (**L^A2^** and **L^B^**) were generated by the GRADE program using the GRADE Web Server (http://grade.globalphasing.org) and applied in the refinement. A GRADE dictionary for SHELXL contains target values and standard deviations for 1,2-distances (DFIX) and 1,3-distances (DANG), as well as restraints for planar groups (FLAT). All displacements for non-hydrogen atoms were refined anisotropically. The refinement of ADP's for carbon, nitrogen and oxygen atoms was enabled by a combination of similarity restraints (SIMU) and rigid bond restraints (RIGU). The contribution of the electron density from disordered counterions and solvent molecules, which could not be modeled with discrete atomic positions were handled using the SQUEEZE routine in PLATON. The solvent mask file (.fab) computed by PLATON was included in the SHELXL refinement via the ABIN instruction leaving the measured intensities untouched.

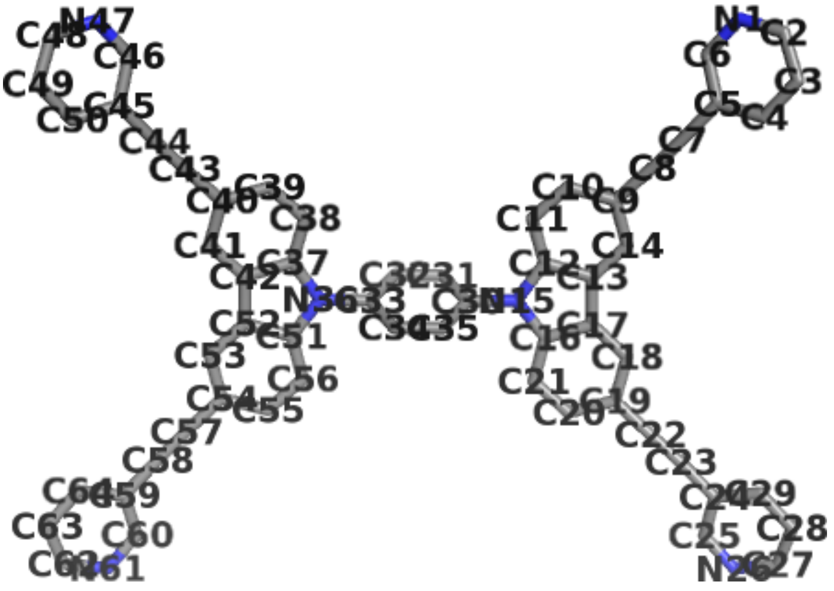

Figure S52. Atomic numbering scheme of residue CPC (ligand $\mathbf{L^{A2}}$).

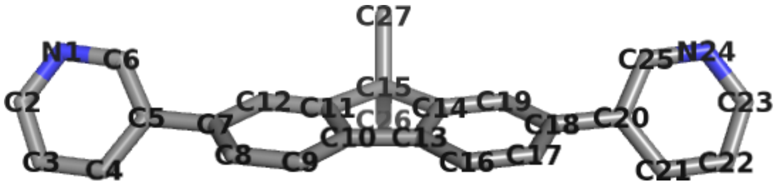

Figure S53. Atomic numbering scheme of residue LFP (ligand $\mathbf{L^{B}}$).

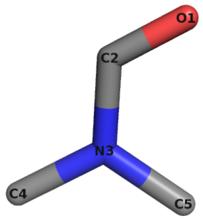

Figure S54. Atomic numbering scheme of residue DMF (DMF solvent molecule).

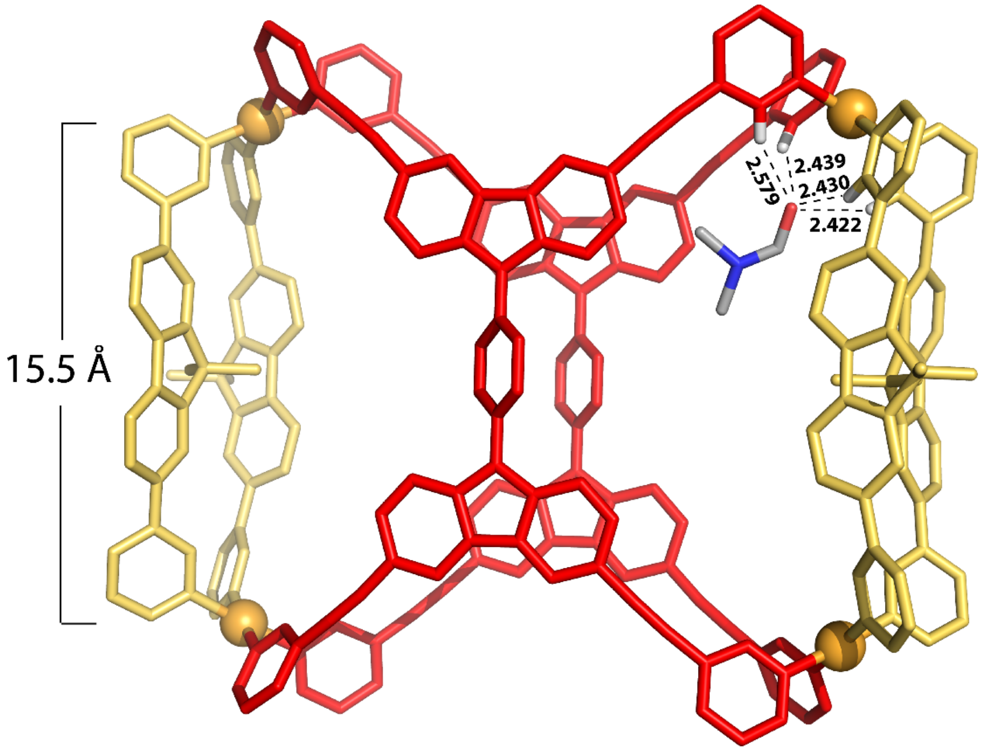

Figure S55. View of cage dimer **D2** crystal structure showing one encapsulated DMF molecule and the hydrogen-bonding environment around its carbonyl group (O···H separations are given in Ångström). Hydrogens, BF_4^- anions and other solvent molecules are omitted for clarity.

Table S6. Structural details of cage dimer **D2**.

Atoms	Distance [Å]	Esd [Å]
Pd1_1 Pd1_1**$2**	15.4528	0.0017
Pd1_1 Pd1_1**$3**	18.3921	0.0021
Pd1_1 Pd1_1**$4**	24.0220	0.0018
Symmetry code: **$2**=+x, 1-y, +z **$3**=1-x, +y, -z **$4**=1-x, 1-y, -z		

6.3. Crystal structure of P2

Colorless needle-shaped crystals of [2G[1]+Pd_6(**L^{A2}**)$_3$(**L^B**)$_6$](BF_4)$_8$ (**P2**) were grown by slow vapor diffusion of Et_2O into a solution of **2G^1@D2** in DMF. Data was collected in-house on a Bruker D8 venture diffractometer equipped with an INCOATEC microfocus sealed tube (Iµs 3.0) using CuK_α radiation at 100 K. The data was integrated with APEX3 and the structure was solved by intrinsic phasing/direct methods using SHELXT[12] and refined with SHELXL[13] for full-matrix least-squares routines on F^2 and ShelXle[14] as a graphical user interface and the DSR[15] program plugin was employed for modeling

*6.3.1. Specific refinement details of **P2**.*

Stereochemical restraints for the ligands (**L^{A2}** and **L^B**) were generated by the GRADE program using the GRADE Web Server (http://grade.globalphasing.org) and applied in the refinement. A GRADE dictionary for SHELXL contains target values and standard deviations for 1,2-distances (DFIX) and 1,3-distances (DANG), as well as restraints for planar groups (FLAT). All displacements for non-hydrogen atoms were refined anisotropically. The refinement of ADP's for carbon, nitrogen and oxygen atoms was enabled by a combination of similarity restraints (SIMU) and rigid bond restraints (RIGU). The contribution of the electron density from disordered counterions and solvent molecules, which could not be modeled with discrete atomic positions were handled using the SQUEEZE routine in PLATON. The solvent mask file (.fab) computed by PLATON was included in the SHELXL refinement via the ABIN instruction leaving the measured intensities untouched.

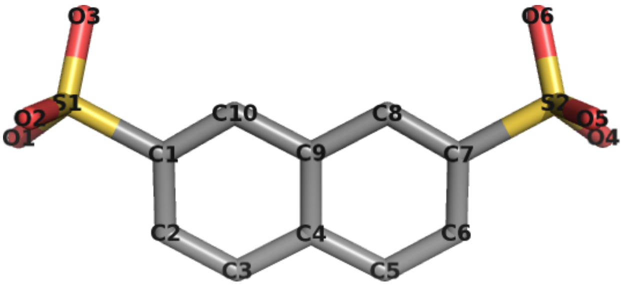

Figure S56. Atomic numbering scheme of residue N7S (guest molecule **G^1**).

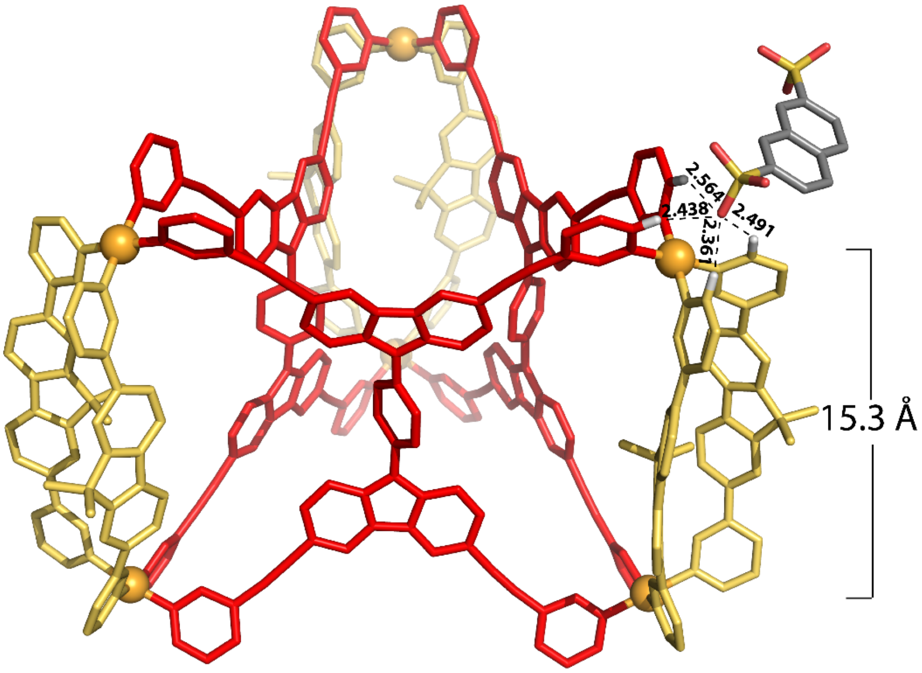

Figure S57. Crystal structure of cage trimer **P2** showing one **G^1** positioned outside the cage boundaries and its hydrogen-bonding environment (O···H separations are given in Ångström). Hydrogens, BF_4^- anions and other solvent molecules are omitted for clarity.

Table S7. Structural details of cage trimer **P2**.

Atoms	Distance [Å]	Esd (Å)	Average
**Pd – Pd Axis**			**15.26**
Pd1_1 Pd2_1	15.2910	0.0046	
Pd3_1 Pd4_1	15.2392	0.0047	
Pd5_1 Pd6_1	15.2628	0.0052	
**Pd – Pd surfaces**			**20.36**
Pd1_1 Pd3_1	20.3740	0.0048	
Pd1_1 Pd5_1	20.2480	0.0051	
Pd3_1 Pd5_1	20.4911	0.0048	
Pd2_1 Pd4_1	20.2469	0.0041	
Pd2_1 Pd6_1	20.4490	0.0051	
Pd4_1 Pd6_1	20.3588	0.0051	
**Pd-Pd Diagonal**			**25.45**
Pd1_1 Pd4_1	25.4188	0.0026	
Pd1_1 Pd6_1	25.4350	0.0031	
Pd3_1 Pd2_1	25.3922	0.0024	
Pd3_1 Pd6_1	25.5741	0.0030	
Pd5_1 Pd2_1	25.4487	0.0022	
Pd5_1 Pd4_1	25.4129	0.0023	

The authors gratefully acknowledge the computing time provided on the Linux HPC cluster at Technical University Dortmund (LiDO3), partially funded in the course of the Large-Scale Equipment Initiative by the German Research Foundation (DFG) as project 271512359.

7. References

[1] C.-H. Chen, T.-H. Hu, T.-C. Huang, Y.-L. Chen, Y.-R. Chen, C.-C. Cheng, C.-T. Chen, *Chem. Eur. J.* **2015**, *21*, 17379-17390.
[2] S. Özgün, E. Asker, O. Zeybek, *J. Mol. Struct.* **2017**, *1127*, 31-42.
[3] J. Luo, X. Zhang, J. Lu, J. Zhang, *Acs Catal.* **2017**, *7*, 5062-5070.
[4] A. Colin-Molina, S. Pérez-Estrada, A. E. Roa, A. Villagrana-Garcia, S. Hernández-Ortega, M. Rodríguez, S. E. Brown, B. Rodríguez-Molina, *Chem. Commun.* **2016**, *52*, 12833-12836.
[5] R. Zhu, J. Lübben, B. Dittrich, G. H. Clever, *Angew. Chem. Int. Ed.* **2015**, *54*, 2796-2800.
[6] G. L. C. Moura, A. M. Simas, *J. Phys. Chem. C* **2010**, *114*, 6106-6116.
[7] P. Thordarson, *Chem. Soc. Rev.* **2011**, *40*, 1305-1323.
[8] S. A. Ewing, M. T. Donor, J. W. Wilson, J. S. Prell, *J. Am. Soc. Mass Spectrom.* **2017**, *28*, 587-596.
[9] P. Pracht, F. Bohle, S. Grimme, *Phys. Chem. Chem. Phys.* **2020**, *22*, 7169-7192.
[10] M. F. Mesleh, J. M. Hunter, A. A. Shvartsburg, G. C. Schatz, M. F. Jarrold, *J. Phys. Chem.* **1996**, *100*, 16082-16086.
[11] F. Neese, *Wiley Interdiscip. Rev. Comput. Mol. Sci.* **2017**, *8*, e1327.
[12] G. Sheldrick, *Acta Crystallogr. Sect. A* **2015**, *71*, 3-8.
[13] G. Sheldrick, *Acta Crystallogr. Sect. C* **2015**, *71*, 3-8.
[14] C. B. Hübschle, G. M. Sheldrick, B. Dittrich, *J. Appl. Crystallogr.* **2011**, *44*, 1281-1284.
[15] a) D. Kratzert, J. J. Holstein, I. Krossing, *J. Appl. Crystallogr.* **2015**, *48*, 933-938; b) D. Kratzert, I. Krossing, *J. Appl. Crystallogr.* **2018**, *51*, 928-934.
